# Targeting CLDN6 in germ cell tumors by an antibody-drug-conjugate and studying therapy resistance of yolk-sac tumors to identify and screen specific therapeutic options

**DOI:** 10.1186/s10020-023-00636-3

**Published:** 2023-03-29

**Authors:** Margaretha A. Skowron, Mara Kotthoff, Felix Bremmer, Katja Ruhnke, Fatma Parmaksiz, Annika Richter, Stefan Küffer, Kirsten Reuter-Jessen, Stella Pauls, Anja Stefanski, Philipp Ströbel, Kai Stühler, Daniel Nettersheim

**Affiliations:** 1 Department of Urology, Urological Research Laboratory, Translational UroOncology, Medical Faculty and University Hospital Düsseldorf, Heinrich Heine University Düsseldorf, Moorenstraße 5, 40225 Düsseldorf, Germany; 2 Institute of Pathology, University Medical Center Göttingen, Göttingen, Germany; 3 Molecular Proteomics Laboratory, Heinrich Heine University Düsseldorf, Düsseldorf, Germany

**Keywords:** Germ cell tumors, Yolk-sac tumor, Therapy, Resistance, CLDN6, Antibody-drug-conjugate

## Abstract

**Background:**

Being the standard-of-care for four decades, cisplatin-based chemotherapy is highly efficient in treating germ cell tumors (GCT). However, often refractory patients present with a remaining (resistant) yolk-sac tumor (YST(-R)) component, resulting in poor prognosis due to lack of novel treatment options besides chemotherapy and surgery. The aim of this study was to identify novel targets for the treatment of YST by deciphering the molecular mechanisms of therapy resistance. Additionally, we screened the cytotoxic efficacy of a novel antibody-drug-conjugate targeting CLDN6 (CLDN6-ADC), as well as pharmacological inhibitors to target specifically YST.

**Methods:**

Protein and mRNA levels of putative targets were measured by flow cytometry, immunohistochemical stainings, mass spectrometry of formalin-fixed paraffin-embedded tissues, phospho-kinase arrays, or qRT-PCR. Cell viability, apoptosis and cell cycle assays of GCT and non-cancerous cells were performed using XTT cell viability assays or Annexin V / propidium iodide flow cytometry, respectively. Druggable genomic alterations of YST(-R) tissues were identified by the TrueSight Oncology 500 assay.

**Results:**

We demonstrated that treatment with a CLDN6-ADC enhanced apoptosis induction specifically in CLDN6^+^ GCT cells in comparison with non-cancerous controls. In a cell line-dependent manner, either an accumulation in the G2 / M cell cycle phase or a mitotic catastrophe was observed. Based on mutational and proteome profiling, this study identified drugs targeting the FGF, VGF, PDGF, mTOR, CHEK1, AURKA, or PARP signaling pathways as promising approaches to target YST. Further, we identified factors relevant for MAPK signaling, translational initiation and RNA binding, extracellular matrix-related processes as well as oxidative stress and immune response to be involved in therapy resistance.

**Conclusions:**

In summary, this study offers a novel CLDN6-ADC to target GCT. Additionally, this study presents novel pharmacological inhibitors blocking FGF, VGF, PDGF, mTOR, CHEK1, AURKA, or PARP signaling for the treatment of (refractory) YST patients. Finally, this study shed light on the mechanisms of therapy resistance in YST.

**Supplementary Information:**

The online version contains supplementary material available at 10.1186/s10020-023-00636-3.

## Background

Germ cell tumors (GCT) are the most common solid tumors among young men aged between 17 and 45 years (Cheng et al. 2018; Park et al. 2018). GCT can be stratified into seminomas (SEM) and non-seminomas (NS), both arising from a misguided or defective primordial germ cell (PGC), resulting in a germ cell neoplasia in situ (GCNIS) (Cheng et al. 2018). The non-seminomatous stem cell-like embryonal carcinomas (EC) have the potential to differentiate into all three germ layers (teratoma) or extra-embryonic tissues, i. e. choriocarcinomas (CC) and yolk-sac tumors (YST) (Cheng et al. 2018). For four decades, cisplatin-based chemotherapy has remained the standard treatment approach for metastatic GCT. Overall, most of the GCT cases can successfully be cured by this strategy, but especially patients with YST receive a poor prognosis due to a high risk of developing therapy resistance, suggesting that the formation of YST represents a therapy escape mechanism of GCT cells (Che et al. 2021).

In the last years, mechanisms and targets were identified that could lead to an altered cisplatin response and subsequent resistance due to decreased uptake, increased efflux or detoxification, and / or modified DNA repair or apoptosis induction (Galluzzi et al. 2012; Skowron et al. 2021a). Cisplatin resistant GCT mainly show deficits in DNA damage repair mechanisms (Skowron et al. 2021a), such as microsatellite instability (MSI), the downregulation of *OCT4*, absent expression of the pro-apoptotic factors *PUMA* and *NOXA*, changes in the microRNA (miR) expression profiles (*miR-17 */ -*106b*, *miR-302*, *miR371*- to *373*), high MDM2 levels, and phosphorylation-dependent translocation of p21 from the nucleus to the cytoplasm (Skowron et al. 2021a; Jacobsen and Honecker 2015; Lobo et al. 2020; Koster et al. 2010; Mayer et al. 2002; Kitayama et al. 2022).

Generally, while pre-clinical trials showed promising results when combing cisplatin-based chemotherapy with drugs specifically targeting tumor cells, there is a need to further investigate on druggable targets linked especially to (resistant) YST to further increase therapy efficacy.

The tetraspanin membrane protein CLDN6 has been identified as a cancer-associated cell surface biomarker, which is rarely expressed in healthy adult tissues (Reinhard et al. 2020; Zhang et al. 2021; Günzel and Fromm 2012; Micke et al. 2014). Also in GCT, based on immunohistochemical stainings, CLDN6 has been described as a potential novel diagnostic marker for SEM, EC, and YST (Ushiku et al. 2012). Specifically, all of the tested SEM (n = 14), EC (n = 10) and YST tissues (n = 12) demonstrated moderate to strong CLDN6 levels (Ushiku et al. 2012). Recently, Mackensen et al. published their first results from a phase I / IIa study using CLDN6 CAR-T-cells (BNT211, BioNTech SE) with or without a CLDN6-encoding CAR-T cell amplifying RNA vaccine (CARVac) for the treatment of seven pre-treated patients suffering from testicular-, ovarian-, or endometrial cancer, as well as soft-tissue sarcoma (Mackensen et al. 2021). Updated data from this trial (NCT04503278) indicated tumor shrinkage as well as CAR T-cell persistence in five GCT patients after six weeks of treatment (Mackensen et al. 2022).

An approach targeting CLDN6 to treat GCT is using related antibodies coupled with a potent cytotoxin, e. g. monomethyl auristatin E (MMAE) (Johansson et al. 2017). Antibody-drug-conjugates (ADC) are notably specific, effective, and well-tolerated therapeutics, since the protein-of-interest-associated antibody domain allows the direction of the conjugated cytotoxin specifically to the tumor cells (Johansson et al. 2017).

In this study, we decided to design a novel ADC (coupled to MMAE) against CLDN6 to examine the cytotoxicity and specificity to target GCT.

Beyond that, since specifically YST cells represent the remaining non-responsive component in refractory GCT, we further aimed at deciphering the molecular mechanisms resulting in the formation of cisplatin-resistant YST. This approach was chosen for the identification of putative multikinase inhibitor-based therapeutic options, which are already in clinical trials and / or approved for the treatment of other tumor entities.

With regard to molecular profiling of YST, we previously observed high molecular similarities between YST and hepatocytes as well as hepatocellular carcinomas (HCC) regarding expression of endodermal factors like *FOXA2*, *SOX17*, *APOA1 / A2 / B*, *ALB, FGA / B / G,* and *GATA3 / 4 / 6* (Ang et al. 2018; D’Amour et al. 2005; Wruck et al. 2021*).* Additionally, we found similarities in signaling pathway activities when comparing YST and HCC (enhanced WNT and BMP signalling) (Ang et al. 2018; D’Amour et al. 2005; Wruck et al. 2021). Based on these observations, we hypothesized that drugs used to treat HCC might also be suitable for the treatment of YST and, thus, screened drugs already in use or in clinical trials in the field of HCC therapy. Additionally, this study deciphered mechanisms of cisplatin therapy resistance in primary and resistant (-R) YST on mutational and proteome level, thereby offering novel therapeutic strategies for the treatment of YST(-R).

## Methods

### Cell culture and standard laboratory techniques

The used (tumor) cell lines were cultured as described previously and summarized in Additional file 5: Table S1A (Skowron et al. 2021b; Burmeister et al. 2022). Polarization of THP-1 cells was described earlier and was based on Genin et al. (2015), Skowron et al. (2022). XTT cell viability assays upon treatment with inhibitors (Additional file 5: Table S1B) were performed as described previously (Skowron et al. 2022). The human phospho-kinase arrays (R&D Systems via Bio-Techne, Wiesbaden, Germany) were carried out according to the manufacturer’s protocol and evaluated using the ‘Protein Array Analyzer’-Plugin for ‘Image J’ (https://imagej.nih.gov/ij/) (Schneider et al. 2012; Carpentier 2022). Further standard laboratory techniques, such as cDNA synthesis, qRT-PCR, flow cytometry-based measurement of apoptosis rates and the cell cycle phase distribution, as well as immunohistochemistry have been described elsewhere (Wruck et al. 2021; Skowron et al. 2021b; Burmeister et al. 2022). See Additional file 5: Table S1B–D for detailed information on the utilized drugs, oligos, and antibodies, respectively.

### Development of antibody-drug-conjugates

Anti-human CLDN6 monoclonal mouse IgG_2B_ antibody (Clone #342927, R&D Systems via Bio-Techne) has been conjugated to MMAE via the drug-linker OSu-Glu-vc-PAB (CLDN6-Glu-vc-PAB-MMAE) by Levena Biopharma (San Diego, CA, USA), resulting in an antibody-drug-ratio of at least 1 : 3.

### Nucleic acid extraction and quality assessment

DNA and RNA were extracted from tumor enriched 2 × 5 µm formalin-fixed, paraffin-embedded (FFPE) slices using the ˋInnuPREP FFPE DNA Kit´ on the ˋInnuPure C16 System´ (Jena Analytika, Jena, Germany) or the ˋMaxwell RNA extraction kit´ (Promega, Walldorf, Germany) according to manufacturer’s recommendations, respectively.

### Library preparation, sequencing and analysis

DNA libraries were prepared using the hybrid capture-based ˋTruSight Oncology 500 Library Preparation Kit´ (Illumina, San Diego, CA, USA) following ˋIllumina’s TruSight Oncology 500 Reference Guide´ (document #1000000067621 v00, Illumina Cambridge, UK) and sequenced on an ˋIllumina NextSeq 500´ instrument. FastQ files were analyzed using ‘CLC Genomics Workbench’ (Qiagen, Hilden, Germany). The reads were mapped on hg19 followed by an initial variant calling.

### Liquid chromatography coupled to mass spectrometry (LC–MS)

For sample preparation, a modified FFPE tissue lysis protocol of Ikeda et al. was applied (Ikeda et al. 1998). Briefly, after deparaffinization by shaking in Xylene for 5 minutes (min), tissues were resuspended in lysis buffer (300 mM TRIS / HCl, 2 % SDS, pH 8.0), shock-frozen in liquid nitrogen and heated at 99 °C for 25 min. Subsequently, tissues were ultrasonicated twice on ice for 20 min with 30 seconds (s) on / off cycles and then shaken for 2 hours (h) at 80 °C and 500 rounds per minute (rpm). After centrifugation, protein concentration of supernatants was determined by the ˋPierce 660 nm Protein Assay´ (Thermo Fisher Scientific, Idstein, Germany). The LC-MS analysis was performed using a modified magnetic bead-based sample preparation protocol as described previously (Hughes et al. 2014). Here, a total of 20 µg protein was reduced using 300 mM DTT and shaking at 56 °C and 1000 rpm for 20 min, followed by alkylation and the addition of 200 µg beads (Sera-Mag SpeedBeads Sigma Aldrich, Taufkirchen, Germany) per sample. Subsequently, 80 % ethanol was added for protein aggregation capture, followed by thrice rinsing steps using 80 % ethanol and once using 100 % ACN. Beads were resuspended in 50 mM TEAB buffer and trypsinized at 37 °C at 1000 rpm. For the LC-MS on the ˋOrbitrap Fusion Lumos Tribrid Mass Spectrometer´ equipped with an ˋAcclaim PepMap 100 C18´ column (75 µm inner diameter, 25 cm length, 2 mm particle size) as a separation column and an ˋAcclaim PepMap 100 C18´ column (75 µm inner diameter, 2 cm length, 2 mm particle size) as a trap column (all equipment from Thermo Fisher Scientific), 500 ng of each sample were used. Data analysis was performed using the ˋProteome Discoverer´ (version 2.4.1.15, Thermo Fisher Scientific), while the RAW files were matched against the human ˋSwissprot´ database (Download: 23.01.2020) and the ˋMaxquant Contaminant´ database (Download: 20.02.2021), using ˋSequestHT´ integrated in the ˋLFQ Tribrid´ processing workflow (Thermo Fisher Scientific).

### Online analysis tools

The TCGA (‘The Cancer Genome Atlas’) GCT cohort was analyzed using cBioportal (https://www.cbioportal.org/) (Gao et al. 2013; Cerami et al. 2012). LC-MS data were analyzed by ‘PCAGO’ (https://pcago.bioinf.uni-jena.de/) for principal component analyses (PCA) (Gerst and Hölzer 2019), while the ‘pandas’, ‘seaborn’, and ‘matplotlib’ libraries were used in ‘Python’ for Pearson’s correlation analyses and visualization via volcano plots (Hunter 2007; Waskom 2012; Mckinney 2010; Reback, et al. 2021; Flyamer 2017). The ‘DAVID Functional Annotation Tool’ using ‘GOTERM_BP_DIRECT’ and ‘GOTERM_MF_DIRECT’ (https://david.ncifcrf.gov) (Dennis et al. 2003) and ‘STRING’ analyses (https://string-db.org/) (Szklarczyk et al. 2019) predicted the molecular functions and protein interactions of deregulated proteins, respectively. The ‘SIGNAL’ web-based analysis platform was used for the identification of signaling cascades (https://signal.niaid.nih.gov/) (Katz et al. 2021). The online platform ‘ImageGP’ (https://www.bic.ac.cn/ImageGP) was used to generate dot plots (Chen et al. 2022). The 'ADMETlab 2.0’ web platform (https://admet.scbdd.com) has been used to screen for the absorption, distribution, metabolism, excretion and toxicity features of tested drugs (Dong et al. 2018; Xiong et al. 2021).

## Results

### CLDN6 as a therapeutic option to target YST

In this study, CLDN6 was chosen to be evaluated as therapeutic targets by using ADC to treat GCT cells.

**Fig. 1:**
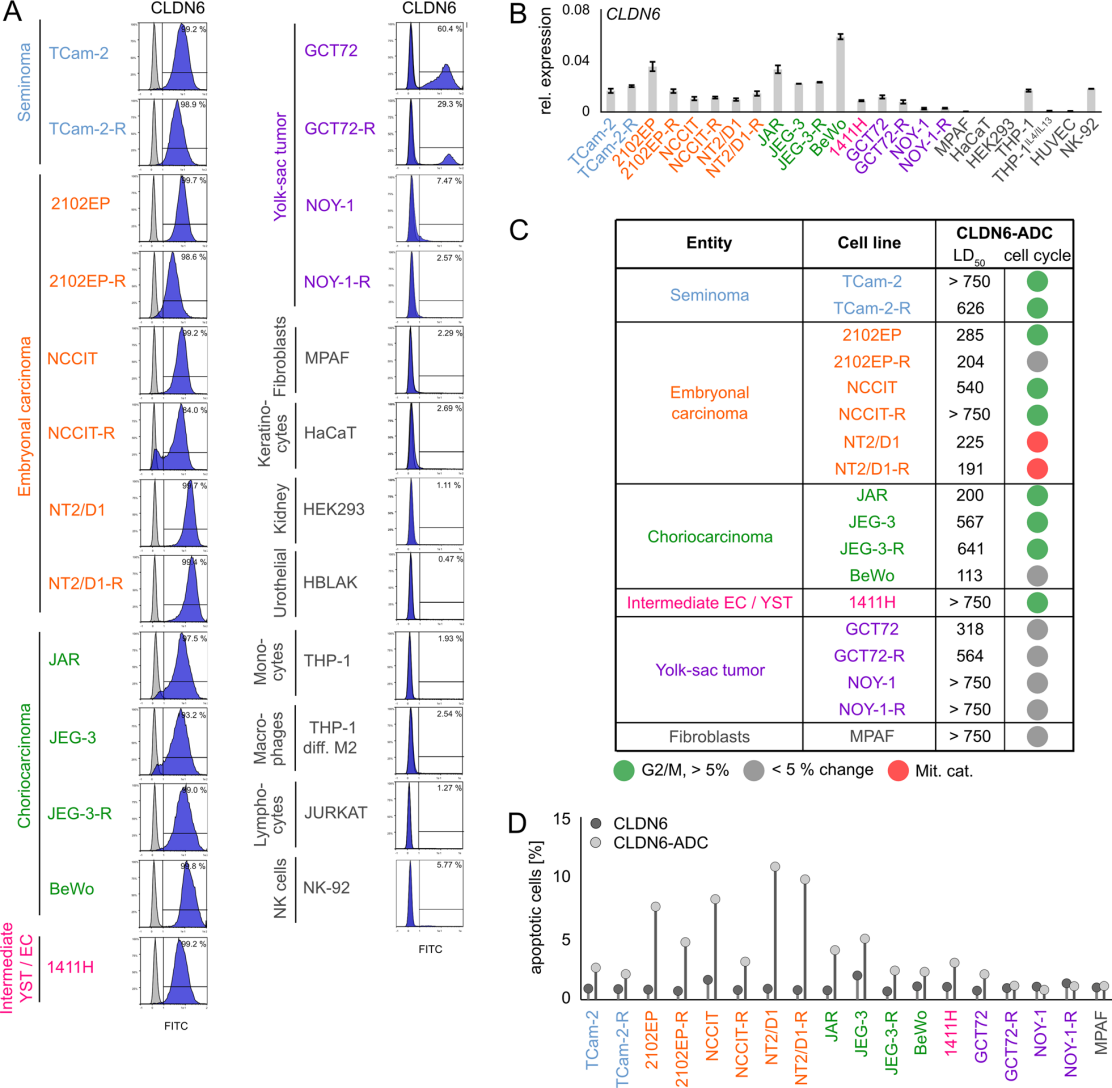
CLDN6-ADC as a novel therapeutic option to target GCTs. **A** Raw flow cytometry data of CLDN6-FITC stained (blue) GCT cell lines, including their cisplatin-resistant sublines, and non-cancerous control cells compared with unstained controls (grey). **B** Relative *CLDN6* expression in GCT cell lines and non-cancerous control cells. *ACTB* and *GAPDH* were used as housekeeping genes. **C** LD_50_ values (ng / ml) acquired by XTT cell viability assays 72 h after treatment with CLDN6-ADC and color-coded changes in cell cycle distribution (G2 / M = green, mitotic catastrophe = red, changes < 5 % = grey) upon treatment with CLDN6-ADC as compared to treatment with the CLDN6 antibody alone in GCT cell lines, including their cisplatin-resistant sublines, as well as fibroblast control cells (MPAF). **D** Lollipop graph summarizing relative number of apoptotic cells in GCT cell lines and fibroblast control cells after treatment with either CLDN6-ADC or CLDN6 antibody alone

While genomic alterations in *CLDN6* were merely observed in the TCGA GCT cohort, we could further show *CLDN6* / CLDN6 being detectable on mRNA and protein level in GCT cell lines including cisplatin-resistant subclones (-R) derived from SEM (TCam-2), EC (2102EP, NCCIT, NT2/D1), CC (JAR, JEG-3, BeWo), and an EC-YST-intermediate (1411H) (Additional file 1: Fig. S1A; Fig. 1A, B). In male YST cells (GCT72(-R)), a predominant CLDN6^−^ and a small CLDN6^+^ population was found, while only low levels of *CLDN6* / CLDN6 were observed in the female YST cell line NOY-1(-R), which were comparable to those of non-cancerous control cells (i. e. fibroblasts, immune cells, keratinocytes) (Fig. 1A, B). Next, the effects on cell viability, apoptosis rates, and the cell cycle distribution of the novel CLDN6-ADC were evaluated by XTT assays and flow cytometry, respectively (Fig. 1C, D). Treatment with the CLDN6-ADC reduced cell viability (LD_50 72 h_ 191 - 641 ng / ml) and induced apoptosis in most CLDN6^+^ GCT(-R) cells (i. e. SEM, EC, CC, and YST cell lines) in comparison to the monoclonal antibody alone (Fig. 1D; Additional file 1: Fig. S1B). After 48 h, EC(-R) cell lines showed the strongest increase in apoptosis rates, while male YST cell lines (GCT(-R)) showed only a mild increase in apoptosis and no alterations in the cell cycle phase distribution (Fig. 1C, D). Female CLDN6^−^ NOY-1 as well as non-cancerous control cells did not respond to CLDN6-ADC treatment (Fig. 1D), while MMAE alone expectedly reduced cell viability in GCT cells at low concentrations (LD_50 72 h_ 0.19 - 10.7 nM) (Additional file 1: Fig. S1B). Moreover, the CLDN6-ADC caused mainly accumulation in the G2 / M cell cycle phase in CLDN6^+^ cells (TCam-2(-R), 2102EP, NCCIT(-R), JAR, JEG-3(-R), 1411H), but not in CLDN6^−^ cells (Fig. 1C, Additional file 1: Fig. S1C). Similar to the treatment with MMAE alone, also mitotic catastrophes were observed upon treatment with the CLDN6-ADC (NT2/D1(-R)) (Fig. 1C, Additional file 1: Fig. S1C). Therefore, the CLDN6-ADC is suitable for the treatment of the GCT subtypes SEM, EC, and CC. In male YST cells, the CLDN6-ADC is less efficient compared to the other GCT entities, while the ADC is not suitable to target female YST cells. In fact, this observation was further validated via immunohistochemical stainings of CLDN6 in YST-R tissues (n = 10), where only 40 % of the investigated cases presented as CLDN6^+^ (Additional file 1: Fig. S1D).

### Identification of novel therapeutic options for YST

Since CLDN6 levels were rather low in YST cells, representing the most aggressive and persistent GCT subtype, eventually, the CLDN6-ADC showed only a moderate efficiency in YST cells. Hence, we characterized therapy-resistant YST to identify putative therapeutic targets, which can be attacked by multikinase inhibitors.

**Fig. 2:**
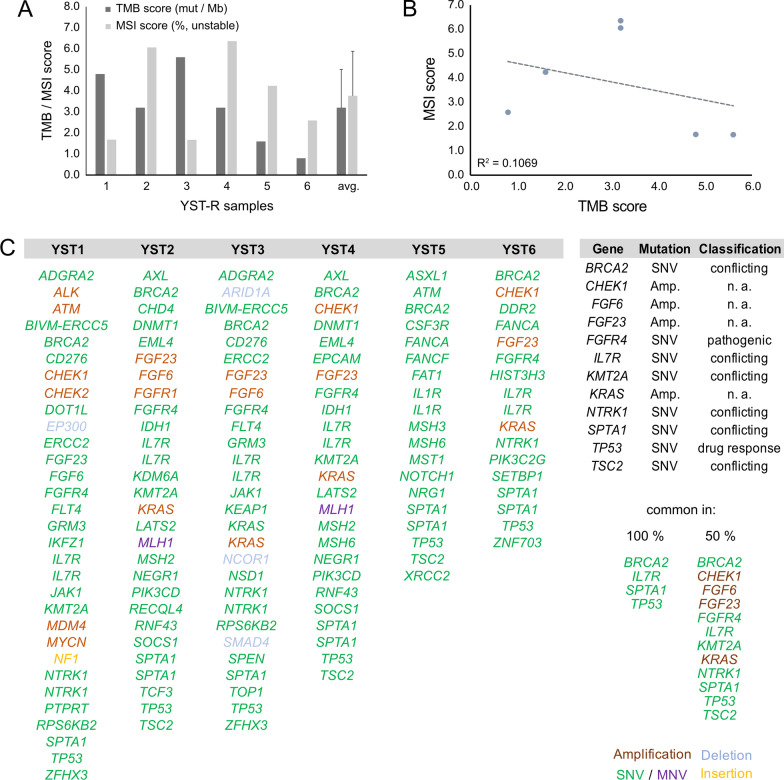
Mutational profiling of YST-R. **A** Tumor mutational burden score (TMB) and microsatellite instability score (MSI) found in six cisplatin-resistant YST samples. **B** Pearson’s correlation plot of TMB and MSI. **C** List of identified individual and common genomic alterations found in six cisplatin-resistant YST tissues

TSO analyses of refractory YST (YST-R) tissues (n = 6) were performed to identify druggable genomic alterations. We detected a mean of 3.2 mut / Mb (0.8 - 5.6 mut / Mb) in the YST-R samples, though, the tumor mutational burden (TMB) did not correlate to the microsatellite instability score (MSI; 3.7 % (1.67 - 6.36 %)) (Fig. 2A, B). Single nucleotide variants (SNV) in *TP53 (c.215C* > *G)*, *BRCA2 (c.7397T* > *C)*, *IL7R (c.197T* > *C, c.412G* > *A)*, and *SPTA1 (c.5077A* > *C)* were observed in all YST-R samples. Furthermore, *CHEK1*, *FGF6*, *FGF23*, and *KRAS* were amplified, but with a low fold change (max 2.2), and SNVs were detected in *FGFR4 (c.1162G* > *A)*, *KMT2A (c.10841T* > *C)*, *NTRK1 (c.53G* > *A, c.1810C* > *T, c.1838G* > *T)*, and *TSC2 (1747G* > *A, c.4285G* > *T)* in at least 50 % of the evaluated samples (Fig. 2C; Additional file 6: Data S1A). Additionally, besides further SNV, amplifications of *ALK*, *ATM*, *CDK4*, *CHEK2*, *FGFR1*, *MDM4*, and *MYCN* were observed in individual samples (Fig. 2C; Additional file 6: Data S1B). Hence, tumor suppressors and DNA repair key players, as well as factors related to the cell cycle, actin skeleton, or the MAPK and FGF signaling pathways were frequently altered in YST-R. It has to be noted that most found mutations were SNV classified as ‘conflicting_interpretations_of_pathogenicity’, suggesting that further work is necessary to narrow down the consequences of these mutations, with the exception of *TP53* and *FGFR4*, whose SNV were classified as affecting ‘drug response’ and ‘pathogenic’, respectively (Additional file 6: Data S1A).

**Fig. 3:**
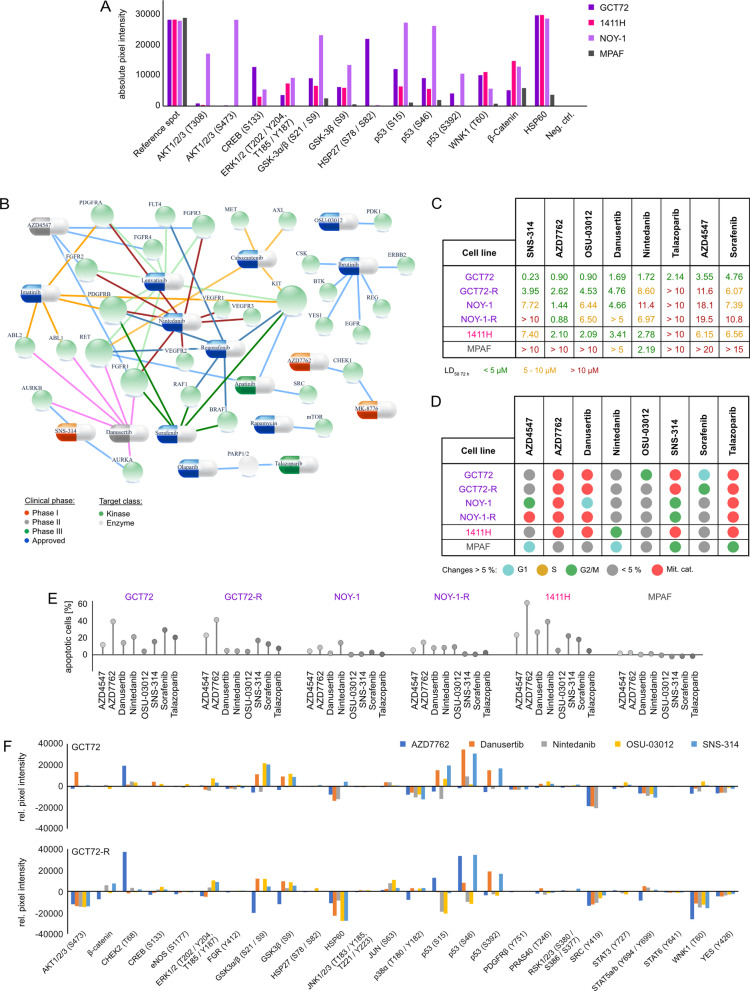
Identification of novel targets for the treatment of YST. **A** Densitometric evaluation of absolute pixel intensities of the 13 most prominent phosphorylation sites in cell lysates from GCT72, 1411H, NOY-1, and MPAF, as measured by the human phospho-kinase array. **B** Graphical illustration of potential pharmacological inhibitors based on genomic alterations found in at least 50 % of YST-R samples and changes on protein level. **C** LD_50_ values (72 h) of GCT72(-R), 1411H, NOY-1(-R), and MPAF upon treatment with the inhibitors selected in (**B**). Inhibitors showing LD_50_ values below 5 µM (green) in GCT72 and higher LD_50_ values in MPAF (5 - 10 µM = yellow, > 10 µM = red) were further evaluated. **D** Color-coded changes in cell cycle distribution (G1 = light blue, S = yellow, G2 / M = green, mitotic catastrophe = red, changes < 5 % = grey) upon treatment with LD_50_ (72 h) concentrations for 24 h with indicated drugs, as compared to the solvent control (DMSO) in GCT72(-R), 1411H, NOY-1(-R), and MPAF. **E** Lollipop graph summarizing relative number of apoptotic cells in GCT cell lines and fibroblast control cells after treatment with LD_50_ (72 h) concentrations for 48 h with the indicated drugs in comparison to the solvent control. Of note, due to high autofluorescence of Nintedanib, all cell types were treated with LD_50_ values (72 h) of GCT72. **F** Densitometric evaluation of relative pixel intensities of the most prominent phosphorylation sites in cell lysates from GCT72 and GCT72-R treated with AZD7762, Danusertib, Nintedanib, OSU-03012, or SNS-314 (24 h, LD_50 72 h_) in comparison to the solvent control (DMSO), as evaluated by the human phospho-kinase array

A phospho-kinase array of YST-like cells (GCT72, NOY-1(-R), 1411H) has been performed to identify signaling molecules and putative YST-specific targets, which were not present in non-cancerous control cells (MPAF) (Additional file 3: Fig. S3A). Compared to fibroblasts, in YST cell lines high levels of AKT1 - 3 (T308, S473), ERK1 / 2 (T202 / Y204, T185 / Y187), GSK3α / β (S21 / S9) and p53 (S15, S46, S392) phosphorylation were detected (Fig. 3D, Additional file 2: Fig. S2 A).

Thus, based on the TSO and phospho-kinase array, we included AZD4547 (FGFR1-4), Nintedanib (FGFR1 - 3), AZD7762, MK-8776 (both CHEK1), and Rapamycin (mTOR) as potential inhibitory drugs to target YST(-R) (Fig. 3B). Additionally, based on the previously described resemblances between YST and HCC, we included drugs to treat HCC, i. e. Sorafenib, Lenvatinib, Regorafenib, and Cabozantinib (Fig. 3B) (Fonseca et al. 2020). The mRNA expression levels of the putative targets of these (multikinase) inhibitors were evaluated in GCT72, 1411H, NOY-1, and MPAF cells. Here, *AURKA / B*, *CSK*, *FGFR1 */ 2, *KIT*, *PARP1 */ 2, *PDGFRA*, *RAF1*, and *YES1* were specifically expressed in the YST cells in comparison to fibroblasts (Additional file 2: Fig. S2B). The mutational status as well as mRNA expression level of respective targets of the putative (multikinase) inhibitors were also evaluated in the TCGA GCT cohort (Additional file 3: Fig. S3). No aberrations were observed in *ABL2*, *AURKA*, *AURKB*, *AXL*, *BRAF*, *BTK*, *FGFR2 */ *3*, *MET*, *PDGFRB*, *REG1A*, and *SRC*, and only few deep deletions (0.7 %) were noted in *ABL1*, *FGFR1 */ *4*, *FLT4*, *MTOR*, *PARP*, *PDK1*, *RET*, while *CSK*, *EGFR*, *ERBB2*, and *RAF1* harbored missense mutations (Additional file 3: Fig. S3 A). *PDGFRA* amplifications and *CHEK1* deletions were noted in 2.1 % and 8.0 % of GCT cases, respectively. Missense mutations as well as amplifications in *KIT* were observed in 15 % of the GCT patients, though mostly in SEM (Additional file 3: Fig. S3A). Regarding the mRNA levels of these putative targets, specific expression profiles / clusters were noted. As such, most SEM tissues showed specifically high levels of *BTK*, *CSK*, *FGFR3*, *KIT*, *ABL2*, *RET*, *PARP2*, *RAF1*, and *PDK1*, while non-seminomatous GCT were positive for *AURKA */ *B*, *FGFR1 */ *4*, *YES1*, *MET*, *ERBB2*, *EGFR*, *FLT4*, *PDGFRA */ *B*, *AXL*, and *SRC* (Additional file 3: Fig. S3B). High expression levels of *ABL1*, *FGFR2*, *MTOR*, *BRAF*, and *PARP1* were seen in both tumor subtypes (Additional file 3: Fig. S3B).

Out of the 17 tested multikinase inhibitors, treatment of GCT72 cells with Danusertib, SNS-314 (both AURKA - C), Nintedanib (VEGFR1 - 3, FGFR1 - 3, PDGFRA / B), Sorafenib (RAF1, BRAF, VEGFR2 / 3, PDGFRB, FLT3, KIT), Talazoparib (PARP1 / 2), OSU-03012 (PDK1), AZD4547 (FGFR1 - 3), AZD7762 (CHEK1), or Rapamycin (mTOR) resulted in LD_50_ values of below 5 µM (Fig. 3C, Additional file 4: Fig. S4A, B). All other drugs showing higher LD_50_ values were excluded from further analyses (Fig. 3C, Additional file 4: Fig. S4 A, B). Effects on cell viability upon treatment with the most potent inhibitors were further evaluated in NOY-1(-R) (YST), 1411H (EC-YST-intermediate), and fibroblasts (MPAF). With the exception of Nintedanib, LD_50_ values in fibroblasts upon treatment with these drugs were above 5 µM, thereby offering a therapeutic window (Fig. 3C, Additional file 4: Fig. S4B). Next, the cell cycle distribution as well as apoptosis induction upon treatment with AZD4547, AZD7762, Danusertib, Nintedanib, OSU-03012, Rapamycin, SNS-314, Sorafenib, and Talazoparib were evaluated in the four YST-like cell lines (GCT72, NOY-1(-R), 1411H) and fibroblast controls (MPAF) (Fig. 3D, Additional file 4: Fig. S4C). In comparison to the solvent control (DMSO), treatment with AZD7762, Danusertib, OSU-03012, SNS-314, Sorafenib, and Talazoparib affected the cell cycle in most GCT cells in a cell line-dependent manner. Prominently, treatment with Danusertib, SNS-314, or Talazoparib resulted in a mitotic catastrophe in YST-like cells after 24 h, while fibroblasts were only affected slightly, showing a small accumulation in the G0 / G1 or G2 / M phase upon treatment with AZD4547 or SNS-314, respectively (Fig. 3D, Additional file 4: Fig. S4C).

Of the four tested YST-like cell lines, the GCT72 and 1411H showed the highest apoptosis induction under most conditions, while the female NOY-1(-R) were the least sensitive YST-like cells (Fig. 3E). Induction of apoptosis remained rather low (< 5 %) in fibroblasts (Fig. 3E). Taking together, treatment with AZD4547 and Nintedanib resulted in apoptosis induction without altering the cell cycle distribution, while treatment with AZD7762, Danusertib, SNS-314, Sorafenib, and Talazoparib not only disrupted the cell cycle, but also induced apoptosis specifically in GCT72 YST cells (Fig. 3D, E; Additional file 4: Fig. S4C). Subsequently, the molecular effects upon treatment with the most sensitive multikinase inhibitors AZD7762, Danusertib, Nintedanib, OSU-03012, and SNS-314 have been evaluated in GCT72(-R) cells (Fig. 3F, Additional file 2: Fig. S2C, D). As such, treatment with the CHEK1 inhibitor AZD7762 enhanced phosphorylation of CHEK2 (T68), while it decreased activity of GSK3α / β (S21 / S9), SRC (Y419), STAT5a / b (Y694 / Y699), and WNK1 (T60) in GCT72(-R) cells. Danusertib treated cells presented elevated phosphorylation of GSK3α / β (S21 / S9) and p53 (S46, S392), while HSP60 and phosphorylation of ERK1 / 2 (T202 / Y204, T185 / Y187), SRC (Y419), and WNK1 (T60) were diminished in both cell lines. Nintedanib treatment resulted commonly in both cell lines in decreased levels of HSP60 and phosphorylation of p53 (S15, S392), SRC (Y419), and WNK1 (T60). Treatment with the PDK1 inhibitor OSU-03012 resulted in several shared abundances on phospho-proteome level in both cell lines (increase in CREB (S133), ERK1 / 2 (T202 / Y204, T185 / Y187), GSK3α / β (S21 / S9) activity), however, phosphorylation of p38α (T180 / Y182), p53 (S15, S46), PRAS40 (T246), STAT3 (Y727), and WNK1 (T60) were oppositional in GCT72(-R) cells. As an AURKA / B inhibitor, treatment with SNS-314 led to increased activity of GSK3α / β (S21 / S9) and p53 (S46, S392) in both cell lines. Additionally, decrease in YES (Y426) phosphorylation was found in both cell lines upon treatment with all here tested inhibitors. Remarkably, all five inhibitors resulted in diminished phosphorylation of AKT1 - 3 (S473) and WNK1 (T60) specifically in the resistant cell line, thereby indicating that the PI3K / PDK1 signaling cascade might be putatively targetable in YST-R (Fig. 3F, Additional file 2: Fig. S2C, D).

**Fig. 4:**
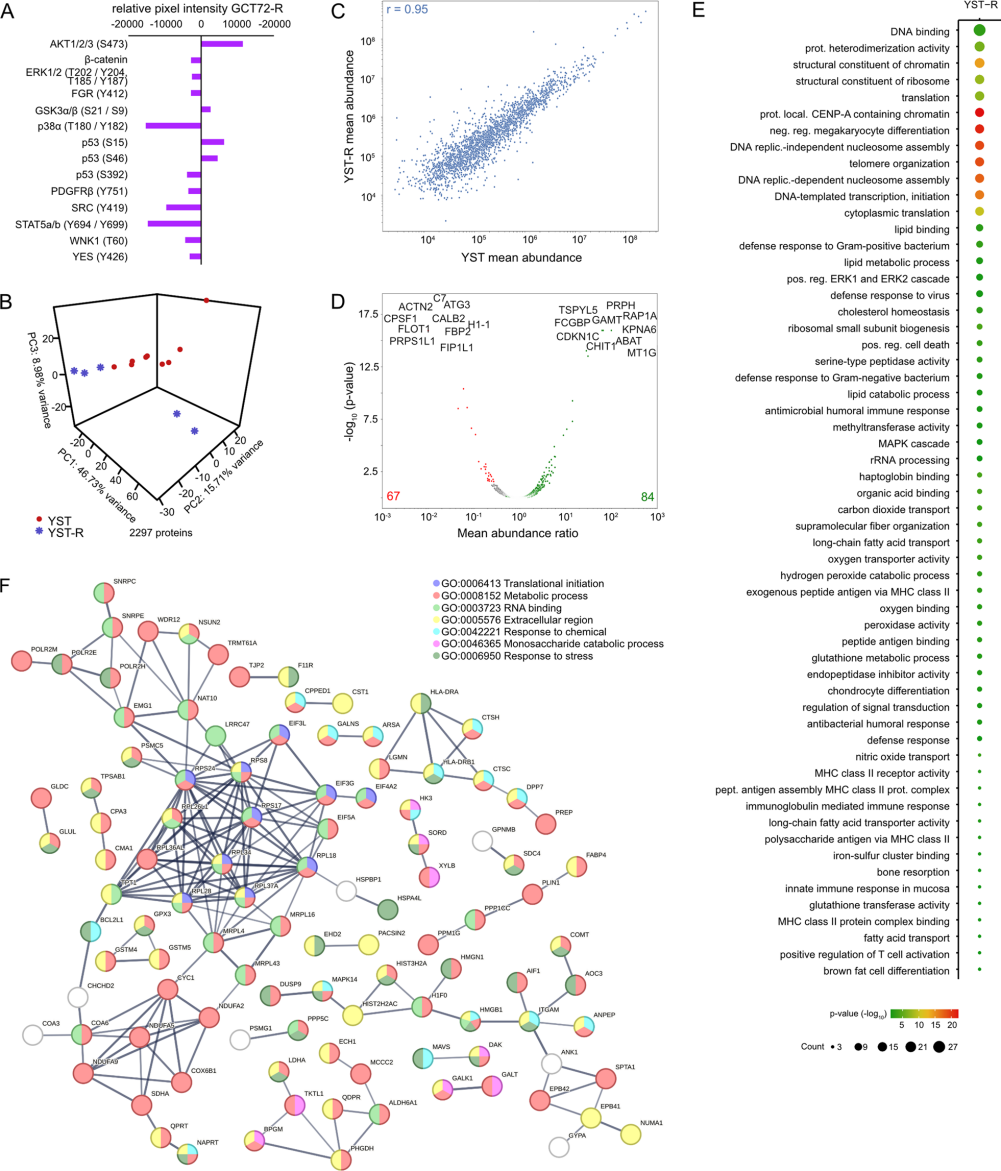
The molecular resistance mechanisms in YST. **A** Densitometric evaluation of relative pixel intensities of the most prominent phosphorylation sites in cell lysates from GCT72-R cells in comparison to the parental cell line, as evaluated by the human phospho-kinase array. **B** PCA plot, **C** Pearson’s correlation plot and **D** Volcano plot of mass-spectrometry data of FFPE-embedded YST(-R) tissues. **E** Enrichment plots showing gene ontology terms found exclusively in YST-R tissues. **F** STRING interaction analysis of YST-R-specific proteins

Consequently, based on a molecular-guided approach, the authors could identify suitable YST-specific candidates that could be targeted using already tested or even approved multikinase inhibitors. Nevertheless, one of the major obstacles during cisplatin-based chemotherapy of YST is the development of resistance mechanisms. At present, this fundamental process is poorly understood. Thus, we subsequently aimed at the molecular characterization of potential mechanisms driving towards a resistant phenotype in YST. Analyzing the differences between YST cells and their resistant sublines, elevated phosphorylation of AKT1 - 3 (S473) and p53 (S15, S46) were seen in GCT72-R cells, while activity of ERK1 / 2 (T202 / Y204, T185 / Y187), FGR (Y412), GSK3α / β (S21 / S9), p38α (T180 / Y182), p53 (S392), PDGFRβ (Y751), SRC (Y419), STAT5a / b (Y694 / Y699), WNK1 (T60), and YES (Y426) was reduced in the resistant subline (Fig. 4A, Additional file 2: Fig. S2C). To further decipher the underlying molecular mechanisms driving YST to a resistant phenotype and identify further targets for the treatment of YST, mass spectrometry-based proteome analyses were performed. A PCA revealed that YST-R samples (n = 5) clustered distinguishably apart from primary YST tissues (n = 9) (Fig. 4B). Even though both tissue types had a high correlation (r^2^ = 0.95) (Fig. 4C), 84 proteins were highly enriched and 67 were significantly depleted (abundance ratio < 0.5 or > 2, p-value < 0.05) in YST-R compared to therapy-naïve YST (Fig. 4D, Additional file 6: Data S1C). A DAVID-based gene ontology and STRING interaction analysis of the proteins enriched in YST-R (abundance ratio > 2) revealed that these factors are involved in translational initiation and RNA binding (e. g. RPL34, RPS8, EIF3G), extracellular matrix (ECM)-related processes (e. g. COL3A1, COL2A1, ITGAX, MFAP5), as well as innate / humoral (pathogen-dependent) immune response (e. g. CD36, HLA-DPB1, HLA-DRB1, LILRB5). Additionally, factors relevant during oxidative stress response and MAPK / Ras / Rap1 signaling (RALB, RAP1A, GNG12, DUSP9, PPP5C) were identified as putative supporting processes in the acquisition of resistance in YST (Fig. 4E, F).

## Discussion

Cisplatin-based chemotherapy is the standard treatment for metastatic GCT. Especially the YST component remains in refractory cases, thereby resulting in a resistant phenotype with limited treatment options. Even though immune checkpoint inhibition has been a promising novel therapeutic approach in various solid tumor entities (Jacob et al. 2021; Dall’Olio et al. 2022) and albeit high levels of PD-L1 and CTLA-4 have been noted in GCT tissues (Lobo et al. 2019; Fankhauser et al. 2015; Cierna et al. 2016), up to now, treatment with immune checkpoint inhibitors, such as pembrolizumab (NCT02499952), durvalumab and tremelimumab (NCT03081923), avelumab (NCT03403777), nivolumab and / or ipilimumab (NCT03333616) showed rather limited efficacy in related clinical trials (Mego et al. 2019; Kawahara et al. 2022; Zschäbitz et al. 2016; Zschäbitz et al. 2017; Tsimberidou et al. 2021; McGregor et al. 2021; Necchi et al. 2019a; Adra et al. 2018). Hence, there is a high necessity of finding novel therapeutic approaches for the treatment of (refractory) GCT.

This study validated the cytotoxic efficacy of a novel ADC targeting CLDN6 as a therapeutic option for GCT. As most ADC, the here presented payload is based on the microtubule inhibitor MMAE (Fu et al. 2022). Beside targeting the antigen-positive cell, also off-target effects for MMAE-based ADC have been noted e. g. for the CD30-ADC brentuximab vedotin (Romano et al. 2019). As such, treatment with brentuximab vedotin not only resulted in apoptosis induction resembling immunogenic cell death, but also led to a pro-inflammatory immune response against lymphoma cells, thereby offering the putative combination with an anti-PD-1 therapy (Cao et al. 2017).

**Fig. 5:**
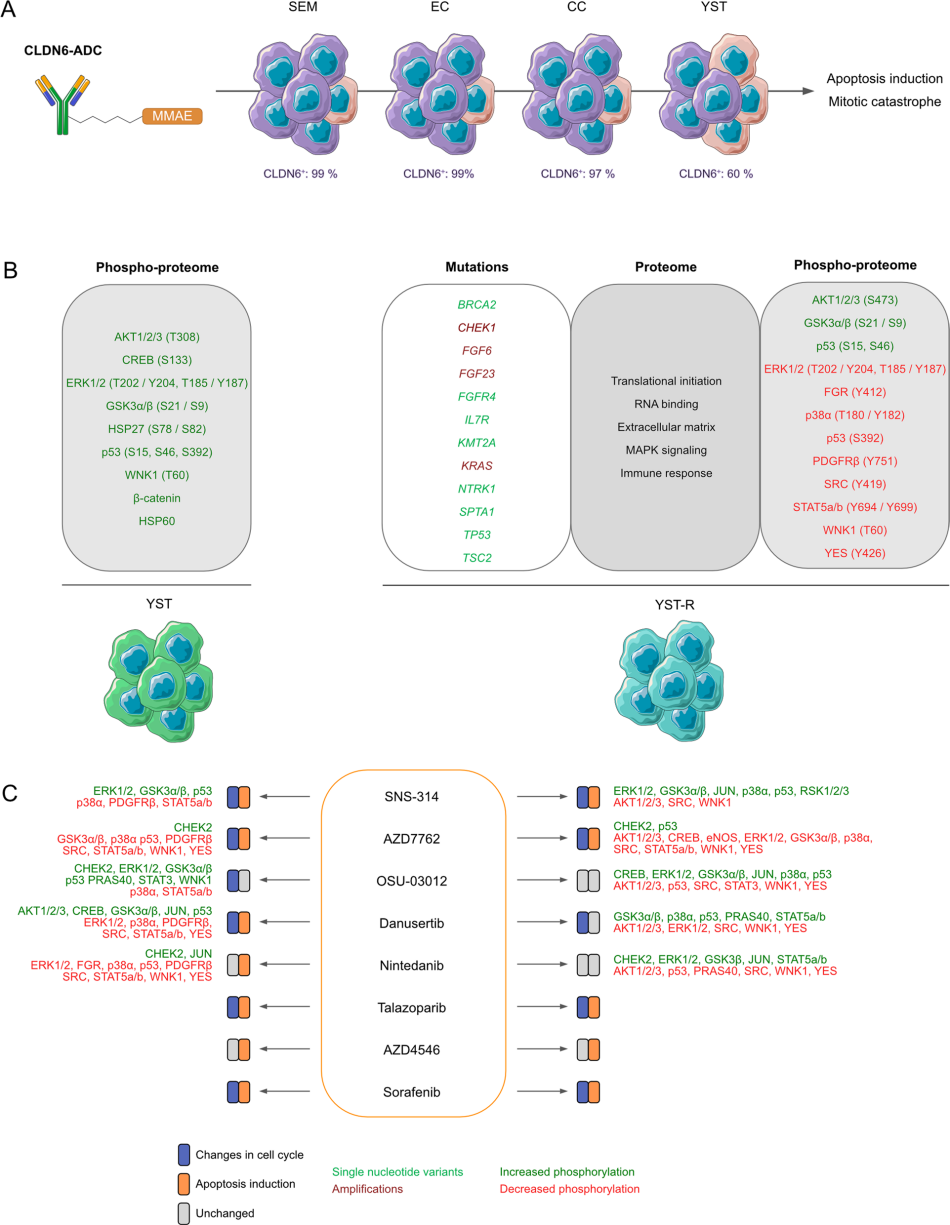
Graphical summary of the observed findings of this study. **A** Targetability of GCT cells by the CLDN6-ADC and provoked effects. **B** Molecular features of YST(-R) cells on DNA and protein level. **C** Molecular effects of the drugs screened as therapeutic option for YST(-R). Created using bioicons (https://bioicons.com/)

Treatment with CLDN6-ADC resulted in mitotic catastrophes and induction of apoptosis in CLDN6^+^ GCT(-R) cells (Fig. 5A). Compared to SEM, EC and CC cells, the CLDN6-ADC was less efficient in GCT72 YST cells (Fig. 5A). By flow cytometry, we measured a CLDN6^+^ and a predominant CLDN6^−^ population in GCT72 cells. Thus, we conclude that the CLDN6-ADC targets the smaller CLDN6^+^ population only, explaining the weaker responses of GCT72 cells in performed analyses on cell cycle phase distribution and apoptosis rates. Due to the low levels of CLDN6 on protein level, female YST cells are not suitable for an attack by the CLDN6-ADC. Beyond GCT, CLDN6 would be also a suitable target for other CLDN6^+^ tumor entities, such as myeloid leukemia, ovarian, endometrial, or urothelial carcinoma (Zhang et al. 2021). Several clinical trials are evaluating the targetability of CLDN6 using immune therapeutic approaches. Currently, a phase 1 / 2 trial using CLDN6-targeting CAR-NK cells is recruiting patients suffering from advanced ovarian, endometrial cancer, or GCT (NCT05410717), while other phase 1 / 2 trials using CLDN6 CAR-T cells (BNT211; NCT04503278) or mRNA encoded bispecific T cell engaging antibody targeting CD3 and CLDN6 (BNT142; NCT05262530) are also currently recruiting for the treatment of solid tumors. However, a phase 2 trial using the monoclonal antibody targeting CLDN6 (ASP1650) for the treatment of refractory GCT patients had to be terminated due to the lack of efficacy (NCT03760081) (Adra et al. 2022). Though, a current study describing the generation and preclinical characterization of a CLDN6-ADC for the treatment of ovarian and endometrial cancer using a patient-derived xenograft model showed reduced tumor volume specifically in CLDN6^+^ tumors (McDermott et al. 2022). Here, a phase 1 trial is currently recruiting to test this CLDN6-ADC (DS-9606a) for the treatment of advanced ovarian cancer and GCT (NCT05394675). As such, one major advantage of using CLDN6-ADC instead of CAR-T-cell-based therapy is its rapid therapeutic accessibility, which, in case of the manufacturing process of CLDN6-CAR-T-cells, might otherwise require a longer time (Reinhard et al. 2020; Rasche et al. 2021). Additionally, in case of autologous CAR-T-cells, harvesting T-cells from heavily pretreated patients might be challenging due to the quality and quantity of T-cells (Rasche et al. 2021). Alternatively, allogeneic T-cells from healthy donors might circumvent these manufacturing issues, however, putative graft versus host disease might be a risk factor (Rasche et al. 2021; Rafiq et al. 2020).

To identify factors that allow for attacking YST components specifically, we further aimed at the identification of novel therapeutic targets based on genomic alterations and changes on the proteome level. Similar to previous observations in primary and resistant GCT tissues (Necchi et al. 2020; Necchi et al. 2019b; Cheng et al. 2020; González-Barrios et al. 2022), we observed an overall low TMB of averaged 3.2 mut / Mb in the YST-R samples. Nevertheless, factors, such as *CHEK1*, *FGF6*, *FGF23*, and *TP53* were commonly amplified in at least 50 % of the evaluated samples (Fig. 5 B). Additionally, we observed AKT1 - 3 (T308 and S473), ERK1 / 2 (T202 / Y204, T185 / Y187), GSK3α / β (S21 / S9) and p53 (S15, S46, S392) phosphorylation in YST samples, while resistant YST cells further showed elevated activity of AKT1 - 3 (S473), GSK3α / β (S21 / S9), and p53 (S15, S46) (Fig. 5B). Together with our previous observations of certain molecular analogies between YST and HCC, we identified novel therapeutic targets whose inhibition resulted in apoptosis induction and / or cell cycle arrest in a drug-dependent manner in YST cells, while fibroblasts remained mostly unaffected, thereby opening a therapeutic window for the treatment of (cisplatin-resistant) YST patients. Overall, several phase 2 studies showed only limited clinical benefit from treatment with either the PARP1 / 2 inhibitor Olaparib (Giorgi et al. 2020) and Veliparib (Mego et al. 2021), c-Met inhibitor Tivantinib (Feldman et al. 2013), ABL1 / 2 / KIT / PDGFRA / B inhibitor Imatinib (Einhorn et al. 2006; Piulats et al. 2007), BRAF / FGFR1 / KIT / PDGFRB / RAF1 inhibitor Sorafenib (Skoneczna et al. 2014), VEGFR1-3 / PDGFR / FGFR / KIT / c-Fms inhibitor Pazopanib (Necchi et al. 2017), mTOR inhibitor Everolimus (Fenner et al. 2018), or VEGFR2 / PDGFRB inhibitor Sunitinib (Feldman et al. 2010; Oechsle et al. 2011; Reckova et al. 2012) in heavily pretreated refractory GCT patients. Nevertheless, most of these studies did not stratify between GCT subtypes, so that no conclusion can be taken with regard to the efficacy for YST patients. Previously described success stories of treatment with tyrosine kinase inhibitors, as it has been shown for human epidermal growth factor receptor 2 (HER2) positive breast cancer (Schlam and Swain 2021) or chronic myeloid leukemia (Kim et al. 2017), are often based on specific mutational patterns, which are rarely found in GCT (Shen et al. 2018). Using mutational profiling as well as proteome-wide analyses, the here presented (pre-clinical) investigation identified (multikinase) inhibitors targeting CHEK1, AURKA - C, VEGFR1 - 3, FGFR1 / 2, PDGFRA / B, mTOR / AKT, RAF1, BRAF, PDGFRB, KIT, or PARP1 / 2 to be considered promising therapeutic options for (refractory) YST patients. As such, an in silico evaluation regarding the absorption, distribution, metabolism, excretion and toxicity (ADMET) of AZD4647, AZD7762, Danusertib, Nintedanib, OSU-03012, SNS-314, Sorafenib and Talazoparib using the ‘ADMETlab 2.0’ web platform revealed that most of these multikinase inhibitors had a good / moderate intestinal absorption and volume distribution (Additional file 7: Data S2). Since clearance was 2.067 - 7.25 mL / min / kg, hepatoxicity should be considered upon treatment with these inhibitors (Additional file 7: Data S2). Moreover, treatment with the most sensitive inhibitors AZD7762, Danusertib, Nintedanib, OSU-03012, and SNS-314 specifically decreased AKT1 - 3 (S473), SRC (Y419), WNK1 (T60), and YES (Y426) phosphorylation especially in YST-R cells (Fig. 5C). Further, treatment with AZD7762, Danusertib, or SNS-314 enhanced JUN (S63) and p53 activity (in at least two of the three evaluated phosphorylation sites), thereby offering novel combined treatment approaches (Fig. 5C).

It is generally believed that cisplatin resistance might occur due to a diminished import, enhanced export, increased detoxification, elevated DNA repair mechanisms, decreased apoptosis induction, and / or augmented alternating signaling pathways eventually resulting in the circumvention of drug-induced cytotoxicity (Galluzzi et al. 2012; Skowron et al. 2021a). Resistance mechanisms in GCT have been often reported to be involved in altered DNA repair mechanisms or apoptosis induction (Skowron et al. 2021a; Jacobsen and Honecker 2015; Vries et al. 2020). Here, we present the first description of putative resistance mechanisms specifically in YST, i. e., besides the previously mentioned role of p53, YST-R were prominently enriched in factors relevant for the translational initiation, RNA binding, immune response, ECM, and oxidative stress response. We identified factors relevant during MAPK signaling to be exclusively enriched in YST-R tissues (Fig. 5B). Additionally, resistant YST cells showed elevated activity of the AKT and p53 pathway. Hence, this study identified signaling cascades that could be targeted using multikinase inhibitors as an alternative treatment approach for (resistant) YST.

## Conclusion

This study offers a decisive groundwork for the understanding of molecular pathways resulting in cisplatin resistance of YST, proposes pertinent therapeutic strategies and offers alternative therapeutic options using (multikinase) inhibitors or an ADC targeting CLDN6.

## Supplementary Information

**Additional file 1: Figure S1.** A) Mutational status and mRNA expression profile of *CLDN6* in the GCT TCGA (The Cancer Genome Atlas) cohort as analyzed through https://www.cbioportal.org/. B) XTT cell viability assays of CLDN6 antibody, CLDN6-ADC, or MMAE treated GCT cell lines and non-cancerous fibroblasts (MPAF) (24  - 96 h). C) Raw flow cytometry data indicating cell cycle distributions of GCT cell lines and MPAF treated with either CLDN6 antibody alone (red), CLDN6-ADC (green), or MMAE (blue) in comparison with untreated controls (grey). D) Immunohistochemical evaluation of CLDN6 in YST-R tissues (n = 10).

**Additional file 2: Figure S2.** A) Raw human phospho-kinase array of various cell lysates (GCT72, 1411H, NOY-1, MPAF). Corresponding membrane layout with the most prominent dots (marked in red) was used for quantification using ImageJ as shown in Fig. 3 A. B) Relative mRNA expression of potential YST target genes in GCT72, 1411H, NOY-1, and MPAF. *ACTB* and *GAPDH* were used as housekeeping genes. C) Raw data and D) densitometric evaluation of GCT72(-R) cells treated with AZD7762, Danusertib, Nintedanib, OSU-03012, or SNS-314 (24 h, LD_50 72 h_) in comparison to the solvent control (DMSO).

**Additional file 3: Figure S3.** A, B) Mutational status (A) and mRNA expression profiles (B) of potential targets to treat YST as found in the TCGA GCT cohort.

**Additional file 4: Figure S4.** A) XTT cell viability assays of GCT72 cells treated with indicated drugs for 24 - 96 h. B) XTT cell viability assays of GCT cell lines GCT72(-R), NOY-1(-R), 1411H, and non-cancerous fibroblasts (MPAF) treated with indicated drugs for 24 - 96 h. C) Raw flow cytometry data of cell cycle distributions of GCT cell lines and MPAF treated with indicated drugs in comparison with solvent controls.

**Additional file 5: Table S1.** List of used A) cell lines, B) inhibitors, C), oligonucleotides, and D) antibodies.

**Additional file 6: Data S1.** Raw and processed mass spectrometry data of YST(-R) tissues and TSO assay data.

**Additional file 7: Data S2.** Structures and ADMET (absorption, distribution, metabolism, excretion, toxicity) prediction of AZD4647, AZD7762, Danusertib, Nintedanib, OSU-03012, SNS-314, Sorafenib and Talazoparib as analyzed by the `ADMETlab 2.0’ web platform (https://admet.scbdd.com/).

## Data Availability

All data generated or analyzed during this study are included in this published article and its supplementary information files. LC-MS raw data generated in this study can be accessed via ProteomeXchange (http://www.proteomexchange.org) (PXD039063).

## Abbreviations

ABTS: 2,2′-Azino-bis(3-ethylbenzothiazoline-6-sulfonic acid
ADC: Antibody-drug-conjugate
ADMET: Absorption, distribution, metabolism, excretion and toxicity
CAF: Cancer-associated fibroblast
CAR: Chimeric antigen receptor
CC: Choriocarcinoma
EC: Embryonal carcinoma
ECM: Extracellular matrix
ELISA: Enzyme-linked immunosorbent assay
FDR: False discovery rate
FFPE: Formalin-fixed, paraffin-embedded
g: Grams
GCNIS: Germ cell neoplasia in situ
GCT: Germ cell tumor
h: Hour
HCC: Hepatocellular carcinoma
L: Liter
LD_50_: Lethal dose, 50%
LC-MS: Liquid chromatography coupled to mass spectometry
M: Molar
m: Meter
min: Minute
miR: MicroRNA
MMAE: Monomethyl auristatin E
MNV: Multi-nucleotide variants
MSI: Microsatellite instability
Mut / Mb: Mutations per megabase
NS: Non-seminoma
p-: Phosphorylation
PCA: Principal component analysis
PGC: Primordial germ cell
R: Resistant
rpm: Rounds per minute
s: Second
SEM: Seminoma
SNV: Single nucleotide variants
TCGA: The Cancer Genome Atlas
TMB: Tumor mutational burden
TSO: TruSight Oncology 500 Sequencing
YST: Yolk-sac tumor
XTT: 2,3-Bis-(2-methoxy-4-nitro-5-sulfophenyl)-2H-tetrazolium-5-carboxanilide
