# Supplementary figures and images for: Targeting CLDN6 in germ cell tumors by an antibody-drug-conjugate and studying therapy resistance of yolk-sac tumors to identify and screen specific therapeutic options
Source: Mol Med. 2023 Mar 29;29:40. doi: 10.1186/s10020-023-00636-3 (PMC10053054; doi:10.1186/s10020-023-00636-3)

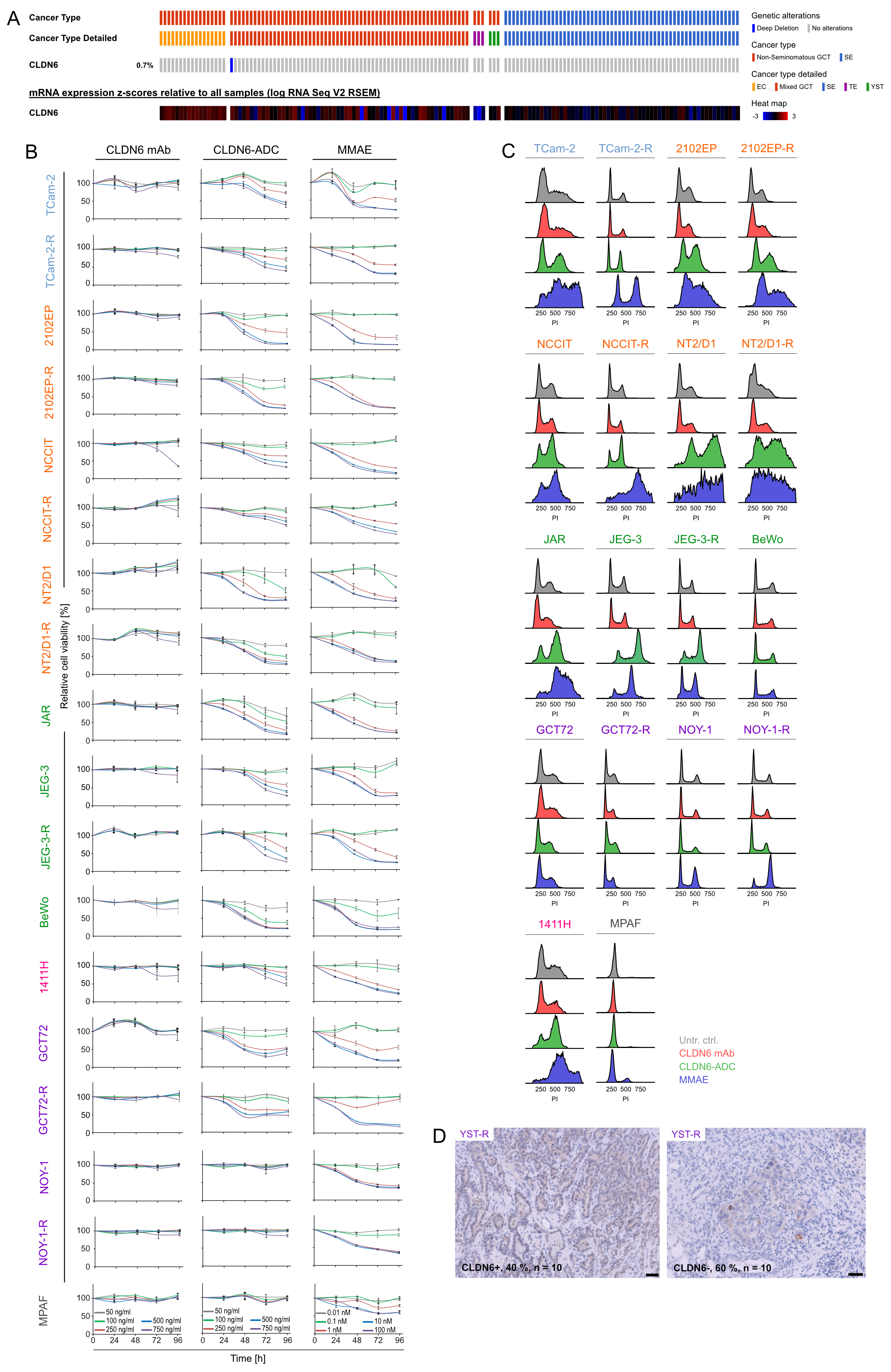

Supplement: Supplementary file 1 — Additional file 1: Figure S1. A) Mutational status and mRNA expression profile of CLDN6 in the GCT TCGA (The Cancer Genome Atlas) cohort as analyzed through https://www.cbioportal.org/. B) XTT cell viability assays of CLDN6 antibody, CLDN6-ADC, or MMAE treated GCT cell lines and non-cancerous fibroblasts (MPAF) (24 - 96 h). C) Raw flow cytometry data indicating cell cycle distributions of GCT cell lines and MPAF treated with either CLDN6 antibody alone (red), CLDN6-ADC (green), or MMAE (blue) in comparison with untreated controls (grey). D) Immunohistochemical evaluation of CLDN6 in YST-R tissues (n = 10). [file 10020_2023_636_MOESM1_ESM.tiff]

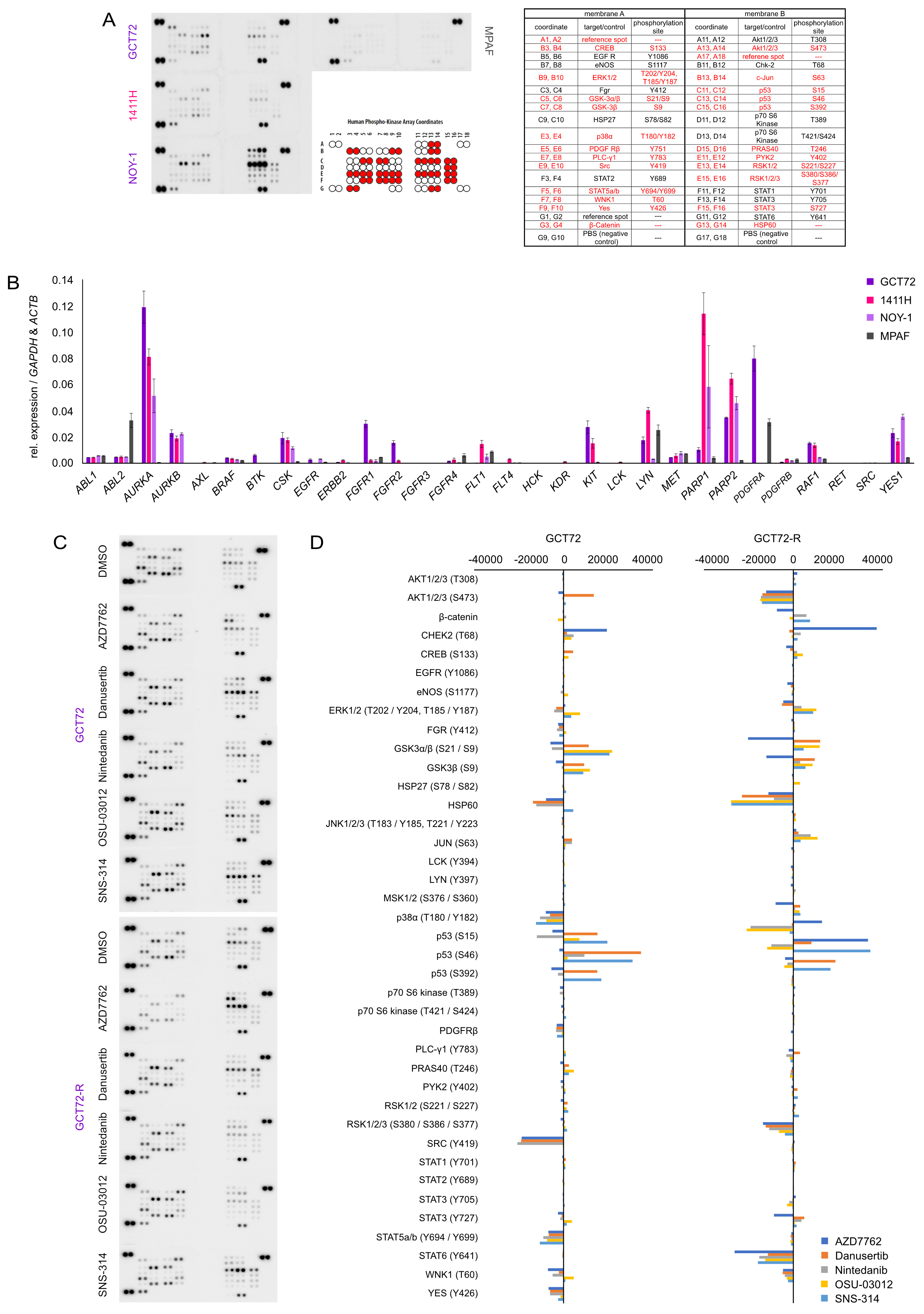

Supplement: Supplementary file 2 — Additional file 2: Figure S2. A) Raw human phospho-kinase array of various cell lysates (GCT72, 1411H, NOY-1, MPAF). Corresponding membrane layout with the most prominent dots (marked in red) was used for quantification using ImageJ as shown in Fig. 3 A. B) Relative mRNA expression of potential YST target genes in GCT72, 1411H, NOY-1, and MPAF. ACTB and GAPDH were used as housekeeping genes. C) Raw data and D) densitometric evaluation of GCT72(-R) cells treated with AZD7762, Danusertib, Nintedanib, OSU-03012, or SNS-314 (24 h, LD50 72 h) in comparison to the solvent control (DMSO). [file 10020_2023_636_MOESM2_ESM.tiff]

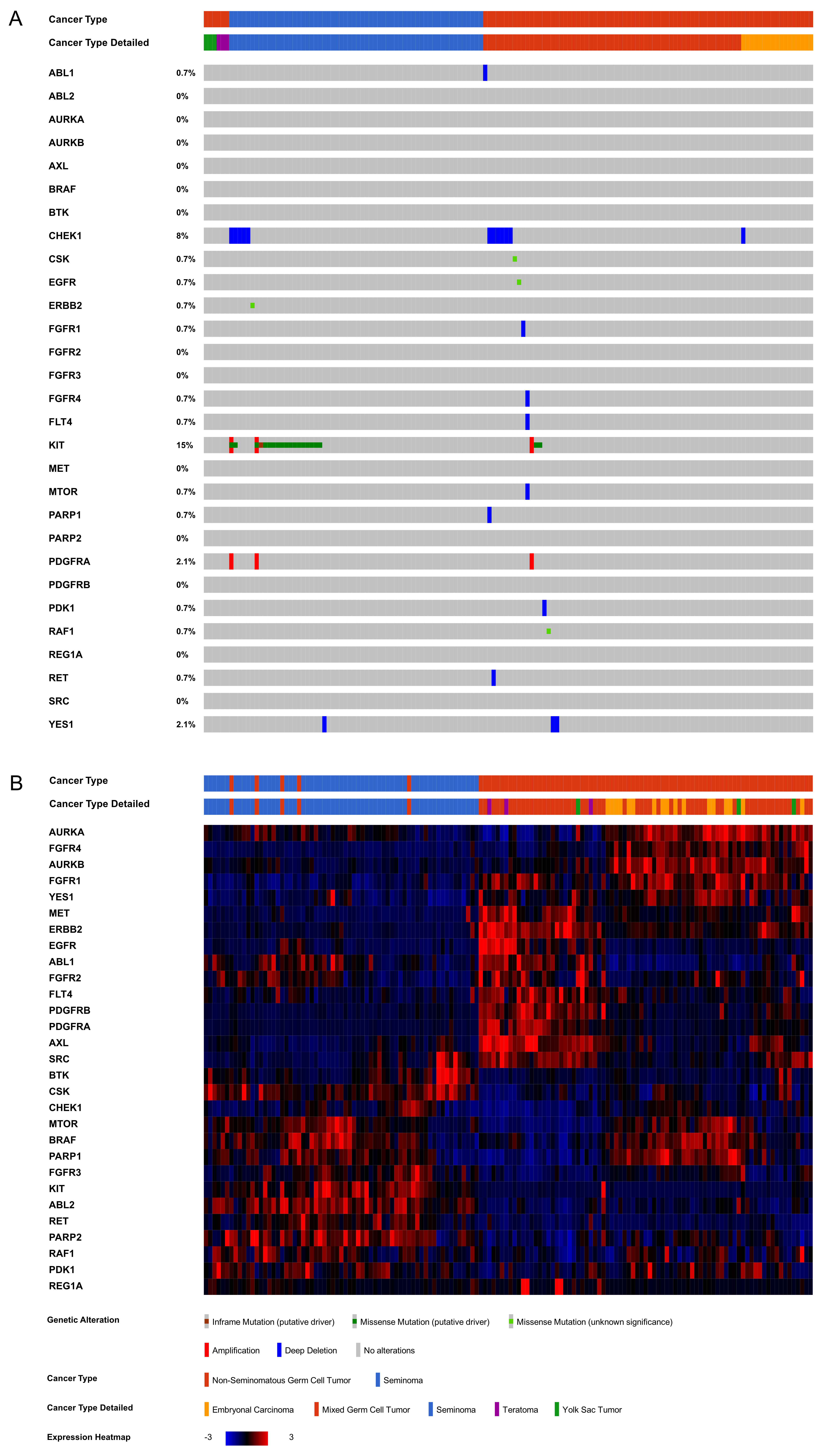

Supplement: Supplementary file 3 — Additional file 3: Figure S3. A, B) Mutational status (A) and mRNA expression profiles (B) of potential targets to treat YST as found in the TCGA GCT cohort. [file 10020_2023_636_MOESM3_ESM.tiff]

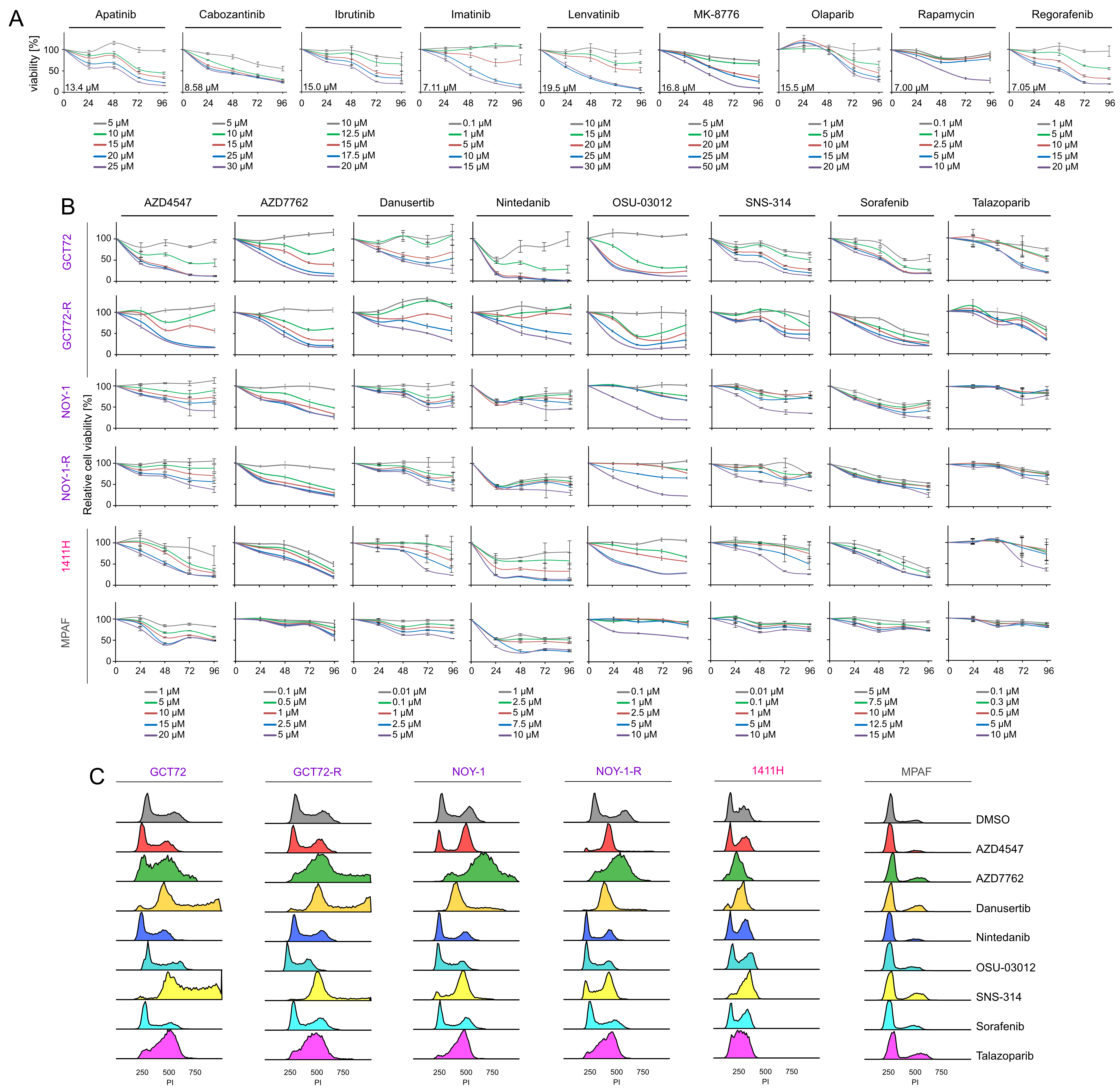

Supplement: Supplementary file 4 — Additional file 4: Figure S4. A) XTT cell viability assays of GCT72 cells treated with indicated drugs for 24 - 96 h. B) XTT cell viability assays of GCT cell lines GCT72(-R), NOY-1(-R), 1411H, and non-cancerous fibroblasts (MPAF) treated with indicated drugs for 24 - 96 h. C) Raw flow cytometry data of cell cycle distributions of GCT cell lines and MPAF treated with indicated drugs in comparison with solvent controls. [file 10020_2023_636_MOESM4_ESM.tiff]
