# Supplementary material for: Targeting CLDN6 in germ cell tumors by an antibody-drug-conjugate and studying therapy resistance of yolk-sac tumors to identify and screen specific therapeutic options
Source: Mol Med. 2023 Mar 29;29:40. doi: 10.1186/s10020-023-00636-3 (PMC10053054; doi:10.1186/s10020-023-00636-3)

Targeting CLDN6 in germ cell tumors by antibody-drug-conjugates and studying therapy resistance of yolk-sac tumors to identify and screen specific therapeutic options

Margaretha A. Skowron^1*^, Mara Kotthoff^1*^, Felix Bremmer^2*^, Katja Ruhnke^1^, Fatma Parmaksiz^1^, Annika Richter^2^, Stefan Küffer^2^, Kirsten Reuter-Jessen^2^, Stella Pauls^4^, Anja Stefanski^4^, Philipp Ströbel^2^,

Kai Stühler^4^, Daniel Nettersheim^1+^

^1^ Department of Urology, Urological Research Laboratory, Translational UroOncology, Medical Faculty and University Hospital Düsseldorf, Heinrich Heine University Düsseldorf, Germany

^2^ Institute of Pathology, University Medical Center Göttingen, Göttingen, Germany

^3^ Department of Urology, University Hospital Ulm, Ulm, Germany

^4^ Molecular Proteomics Laboratory, Heinrich-Heine-University Düsseldorf, Düsseldorf, Germany

* contributed equally

**Data S2**

^+^ corresponding author:

Prof. Dr. Daniel Nettersheim

Department of Urology

Urological Research Laboratory

Translational UroOncology

Medical Faculty and University Hospital Düsseldorf

Heinrich Heine University

Moorenstraße 5

40225 Düsseldorf

Germany

E-Mail: Daniel.Nettersheim@med.uni-duesseldorf.de

Phone: +49 211 81 06731

Supplemental Data 2: Structures and ADMET (absorption, distribution, metabolism, excretion, toxicity) prediction of AZD4647, AZD7762, Danusertib, Nintedanib, OSU-03012, SNS-314, Sorafenib and Talazoparib as analyzed by the ‚ADMETlab 2.0‘ web platform (https://admet.scbdd.com/; Dong, J Cheminform, 2018, PMID: 29943074; Xiong, Nucleic Acids Res, 2021, PMID: 33893803).


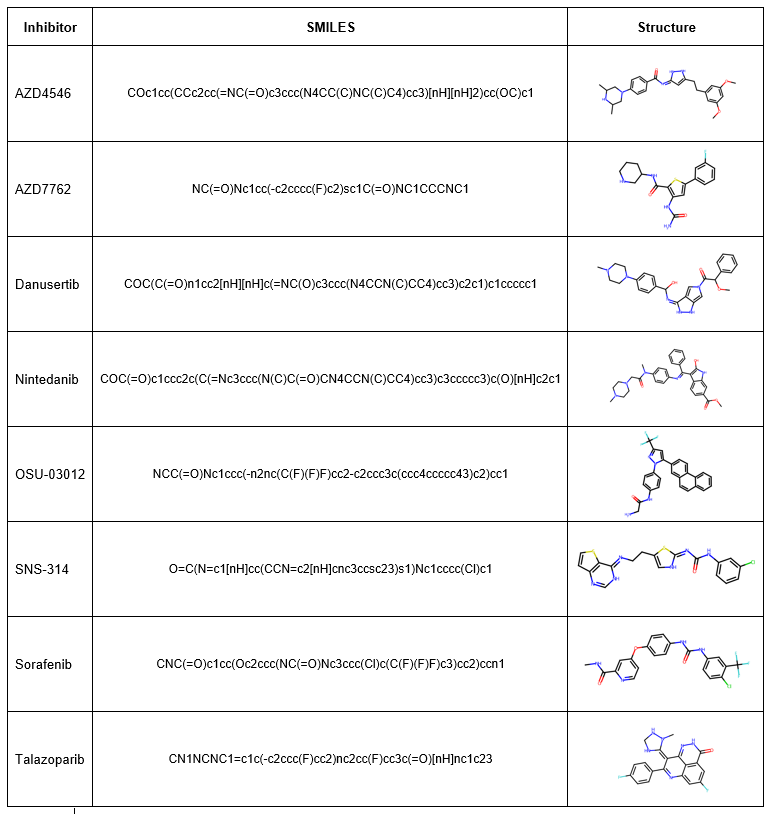


**AZD4546**
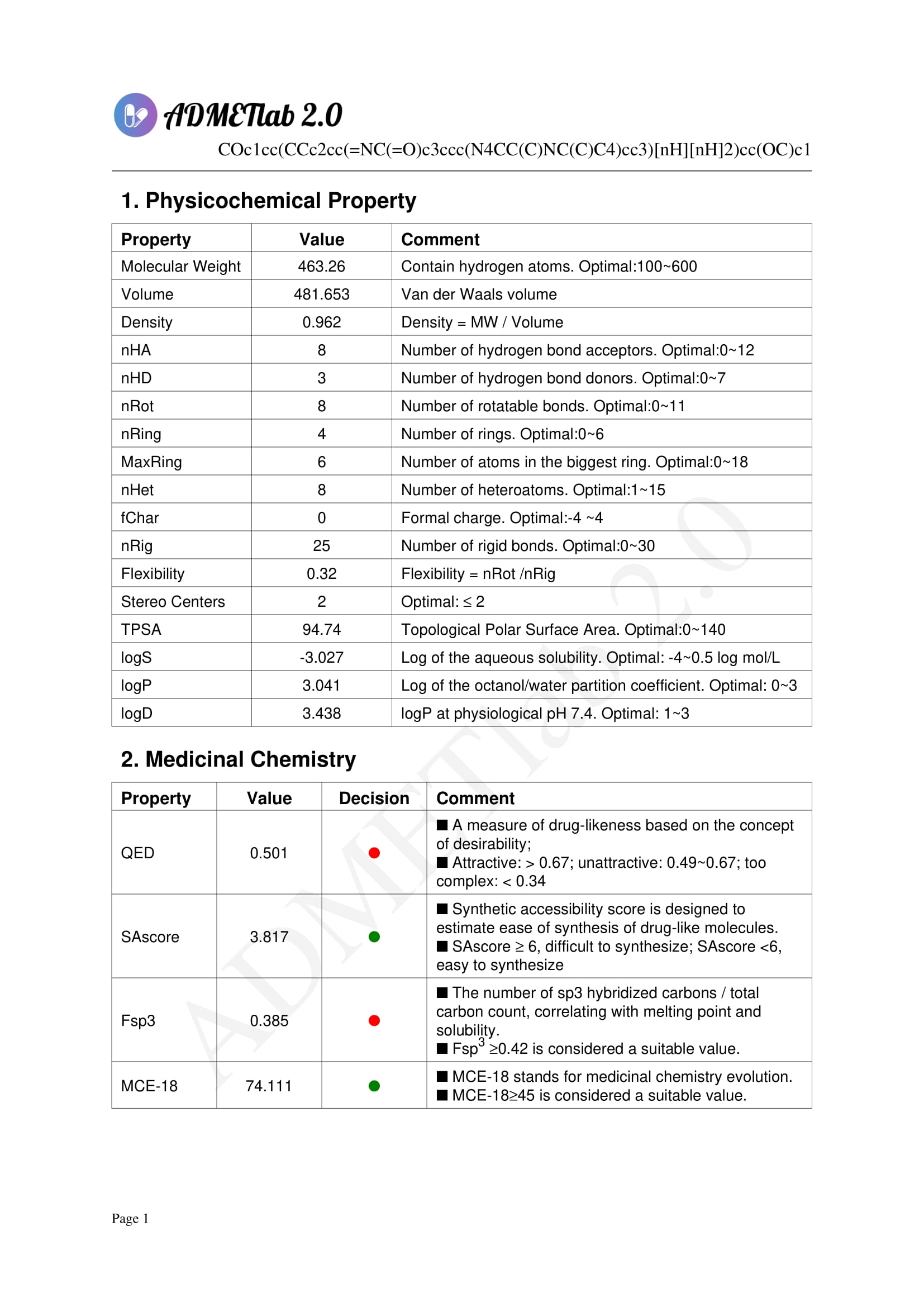

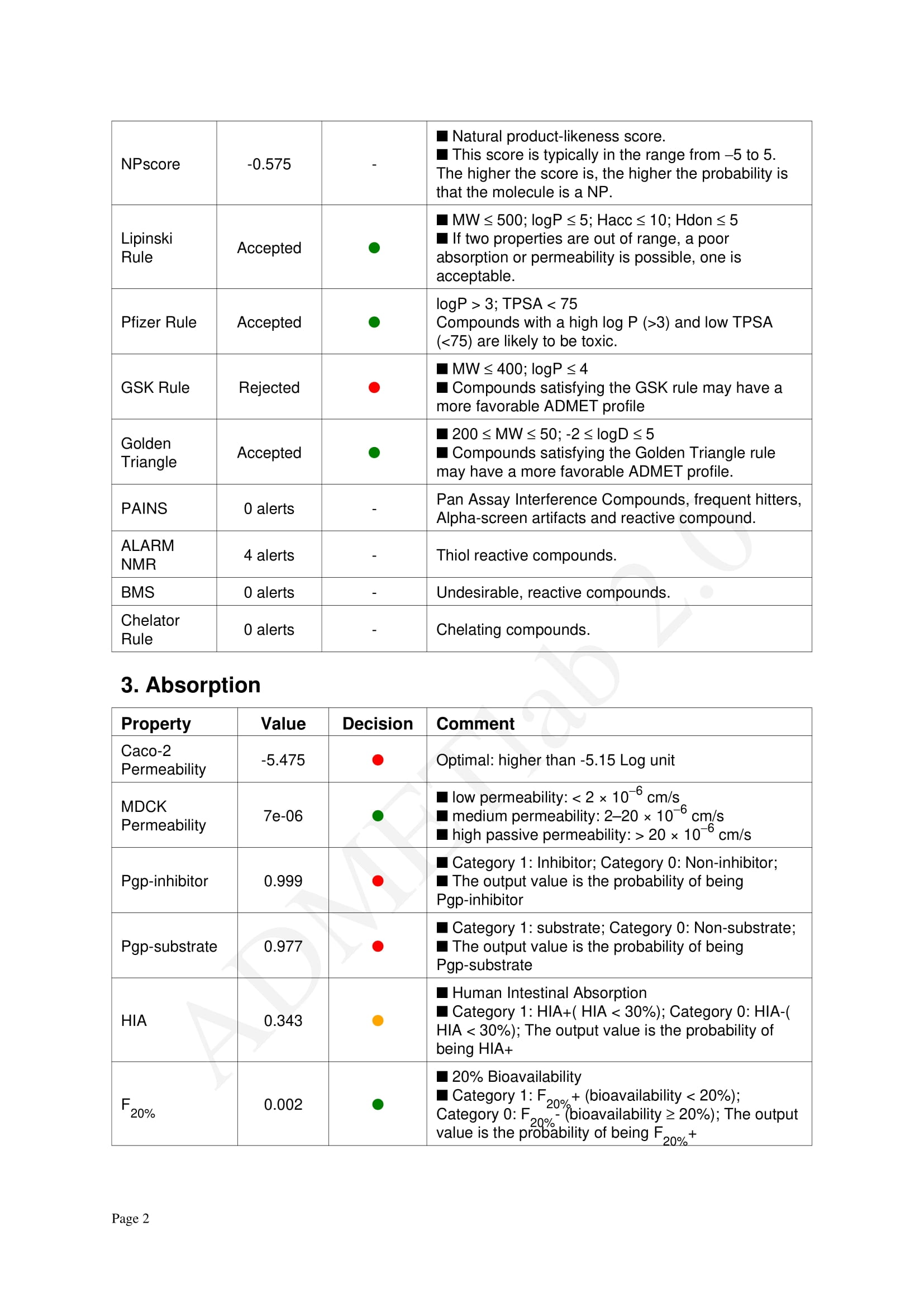

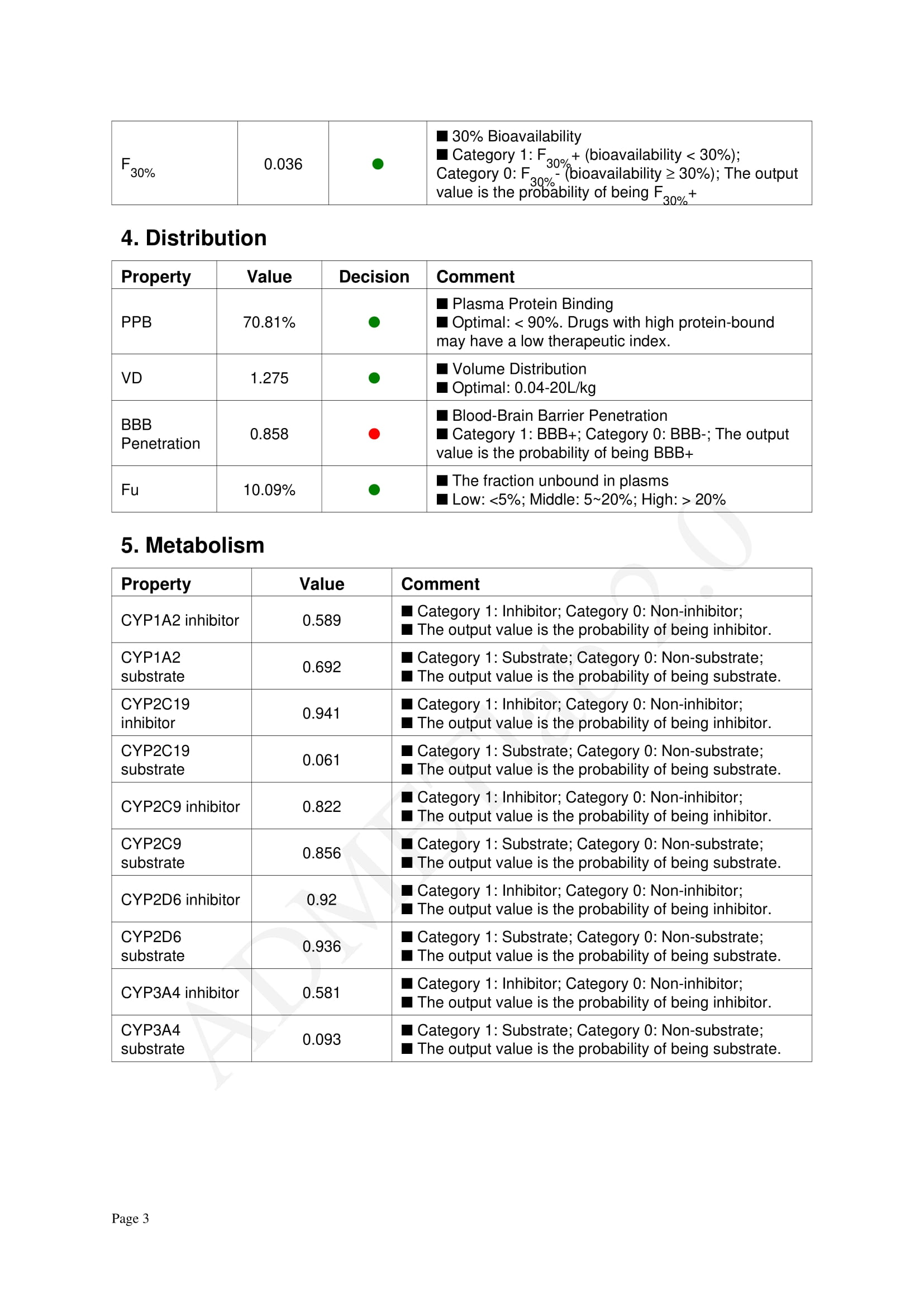

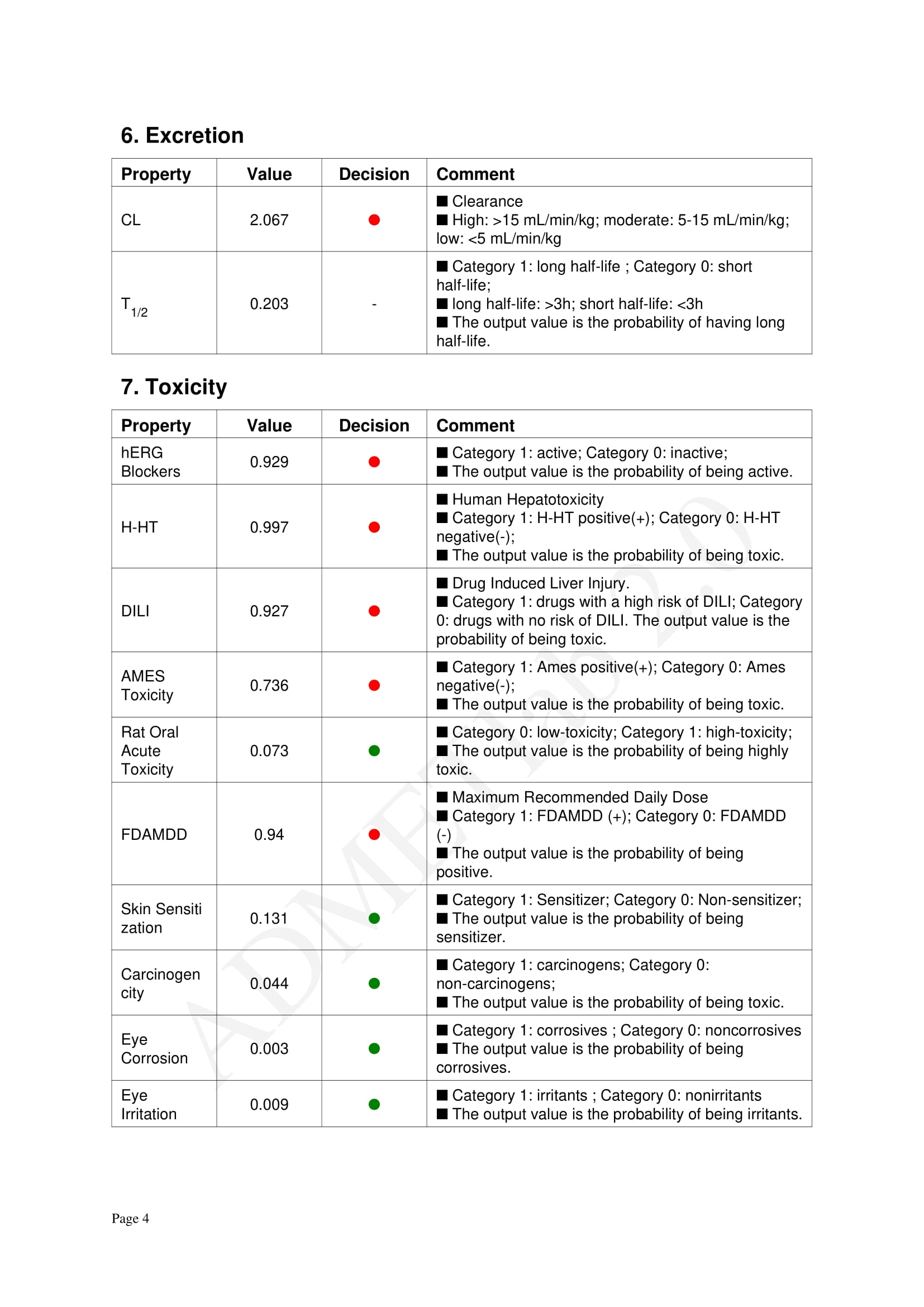

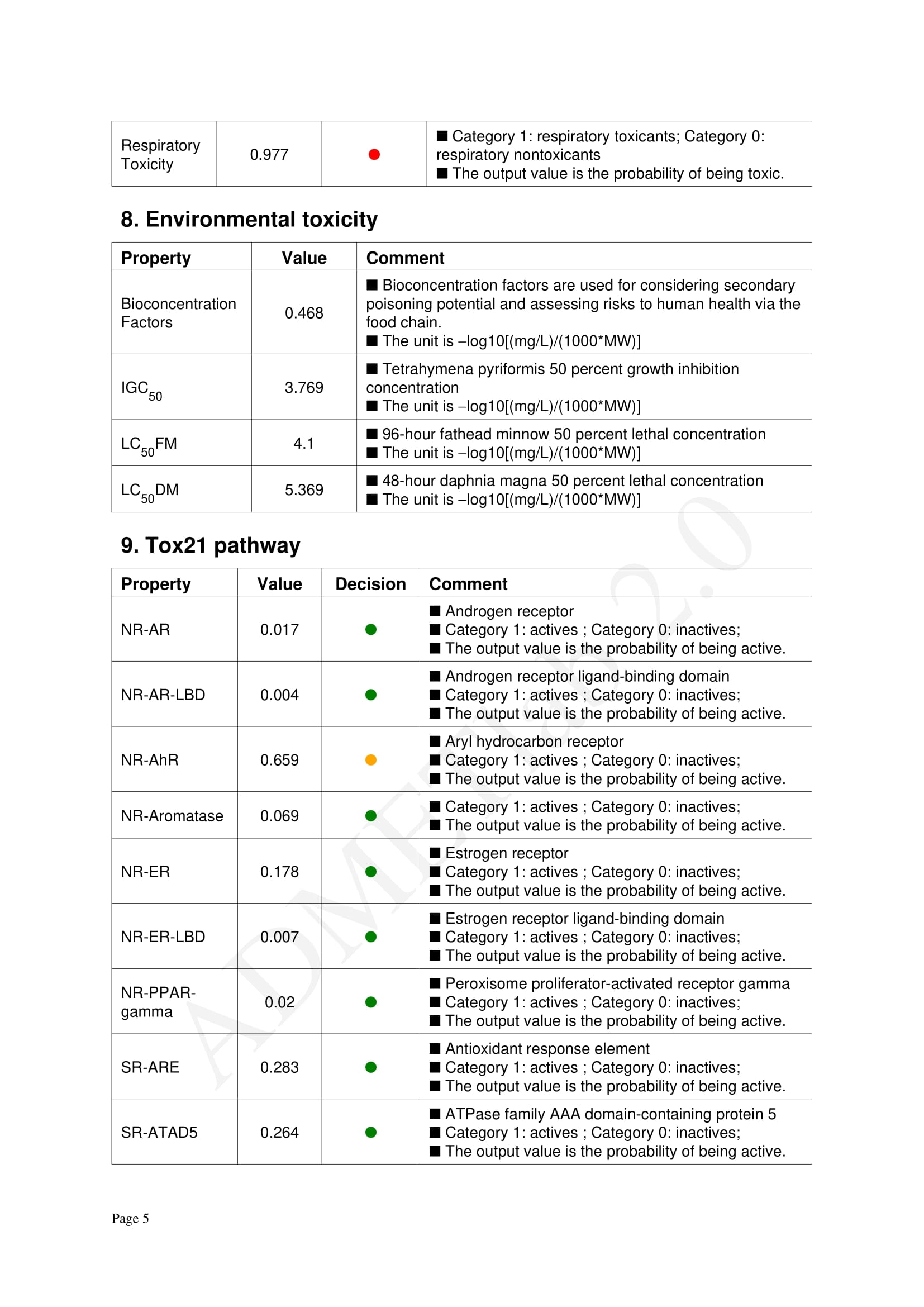

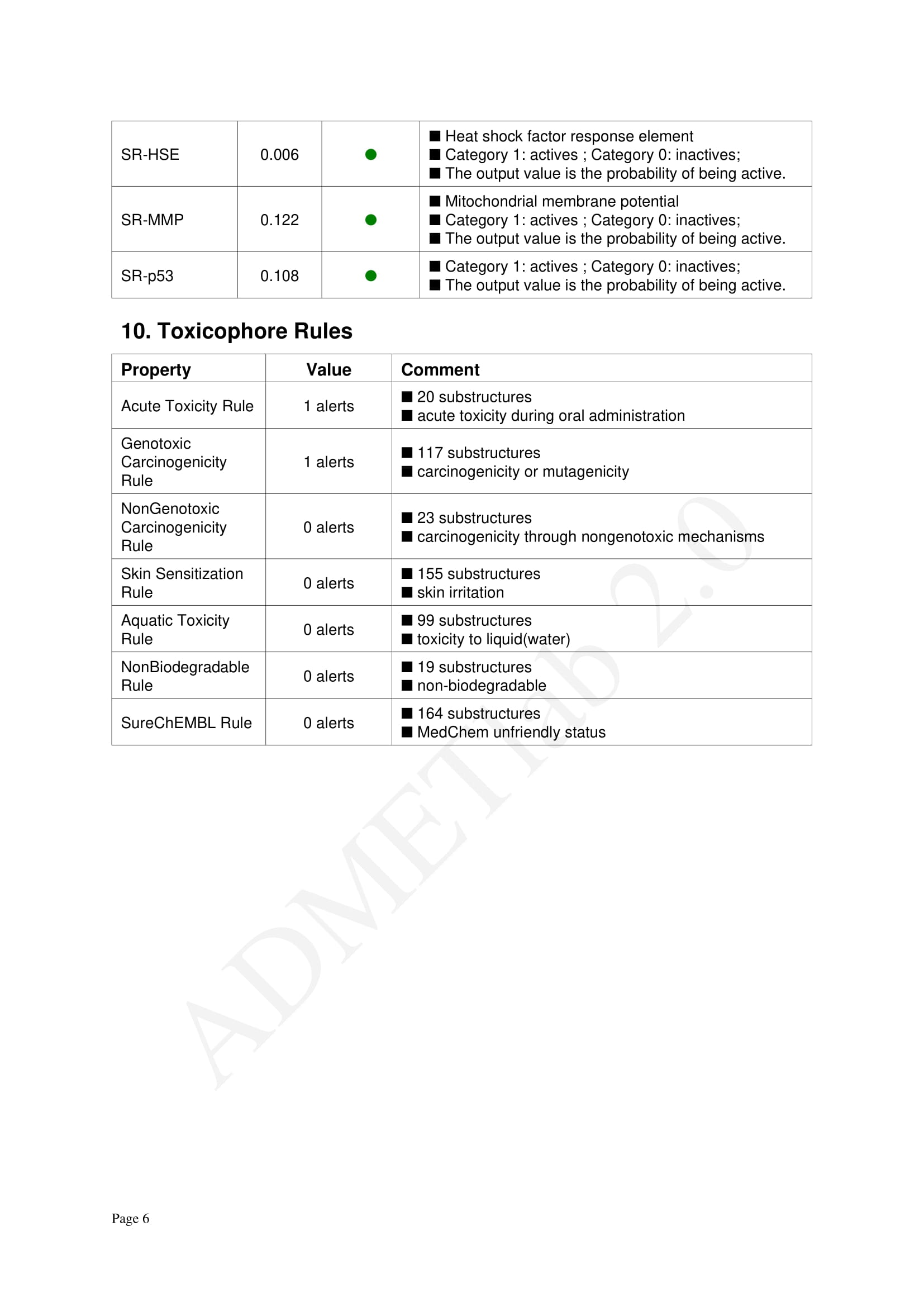


**AZD7762**
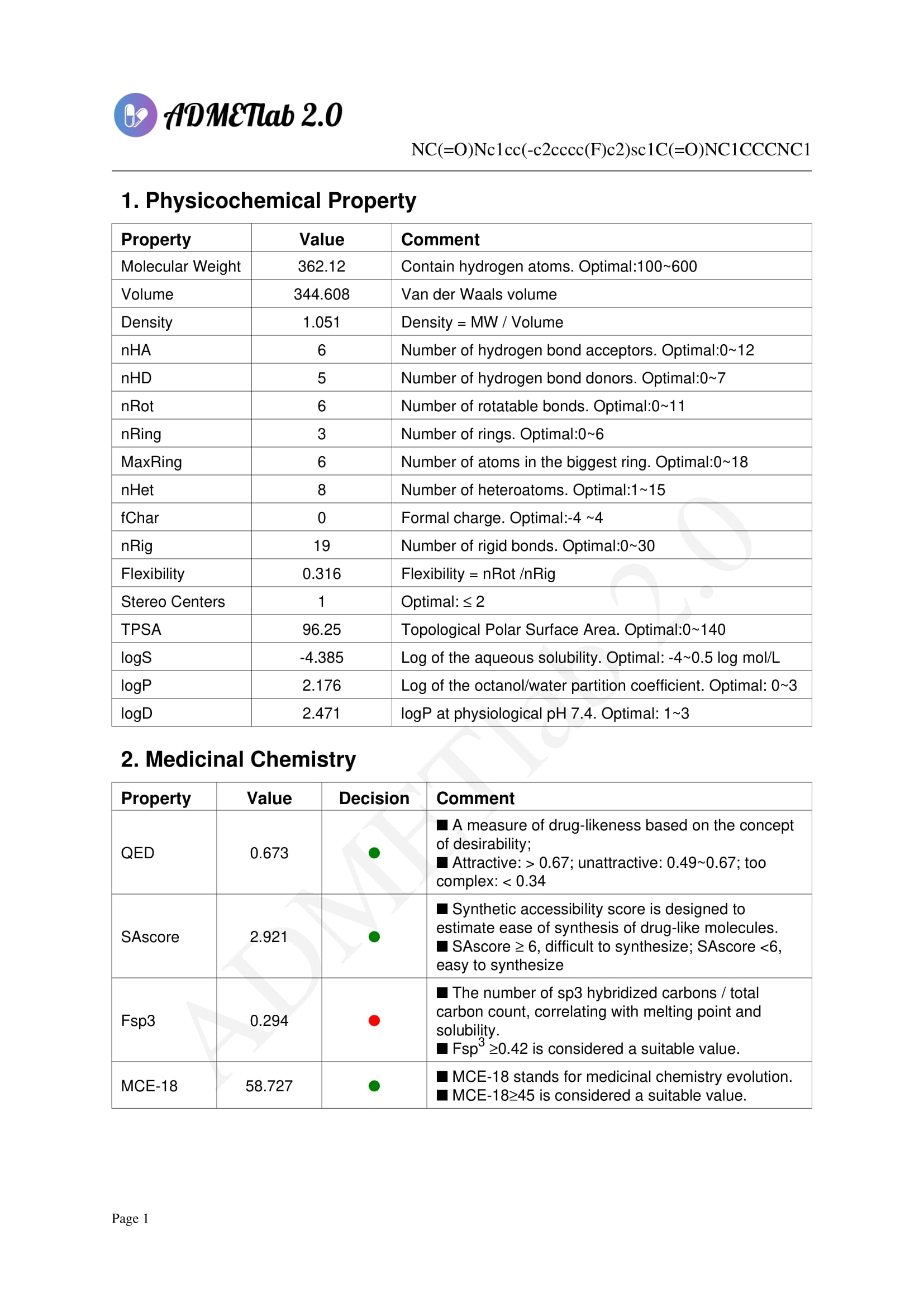

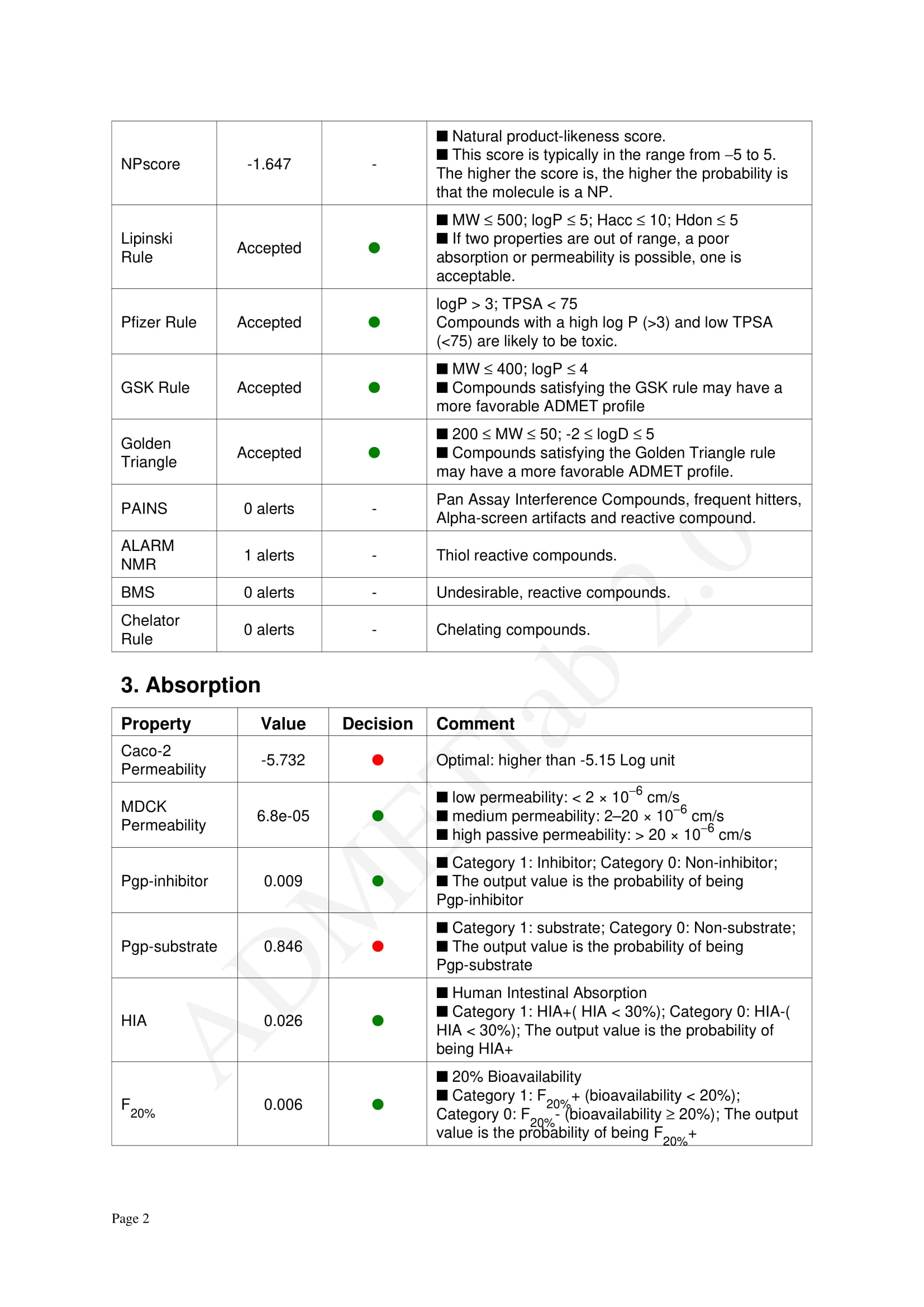

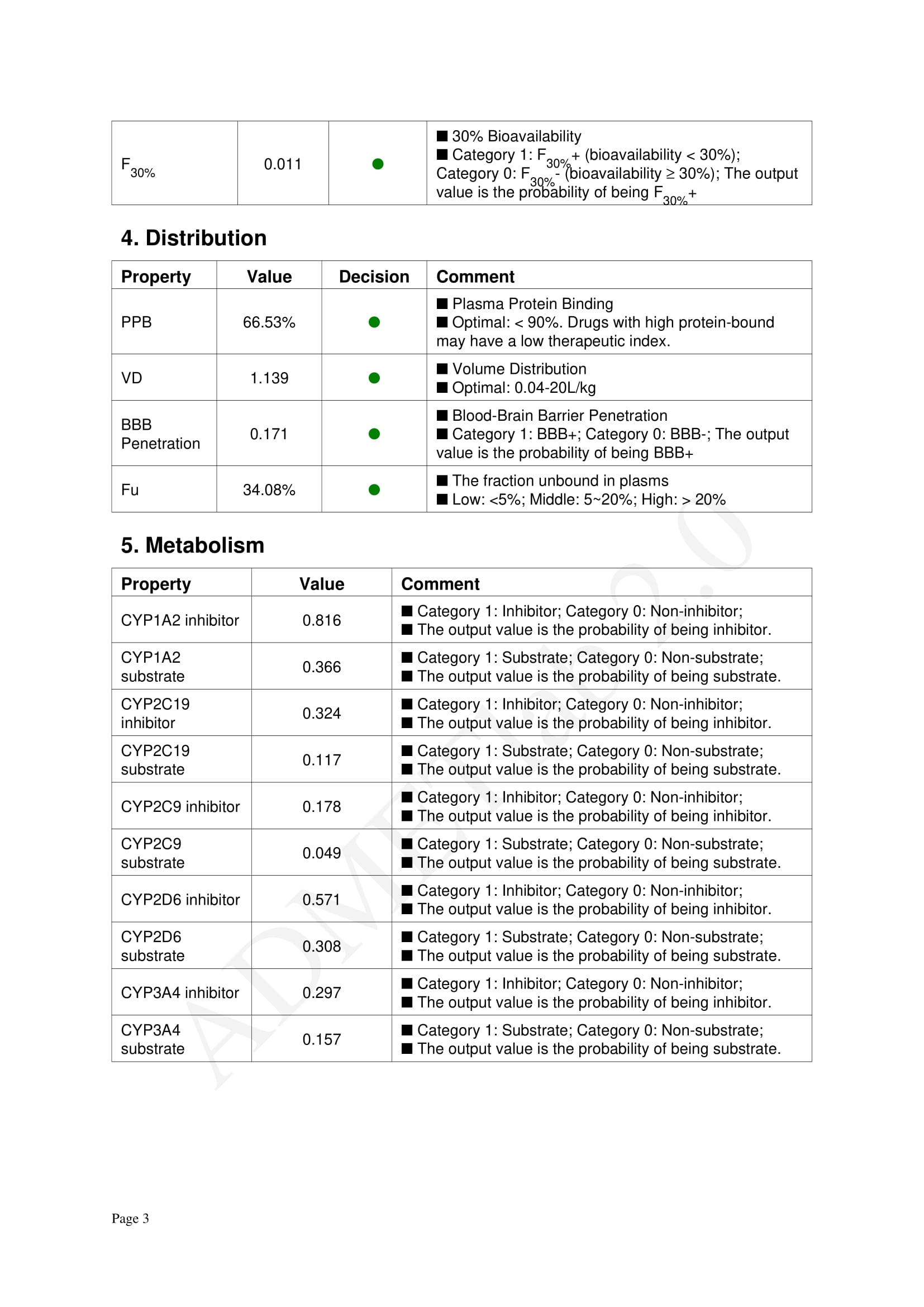

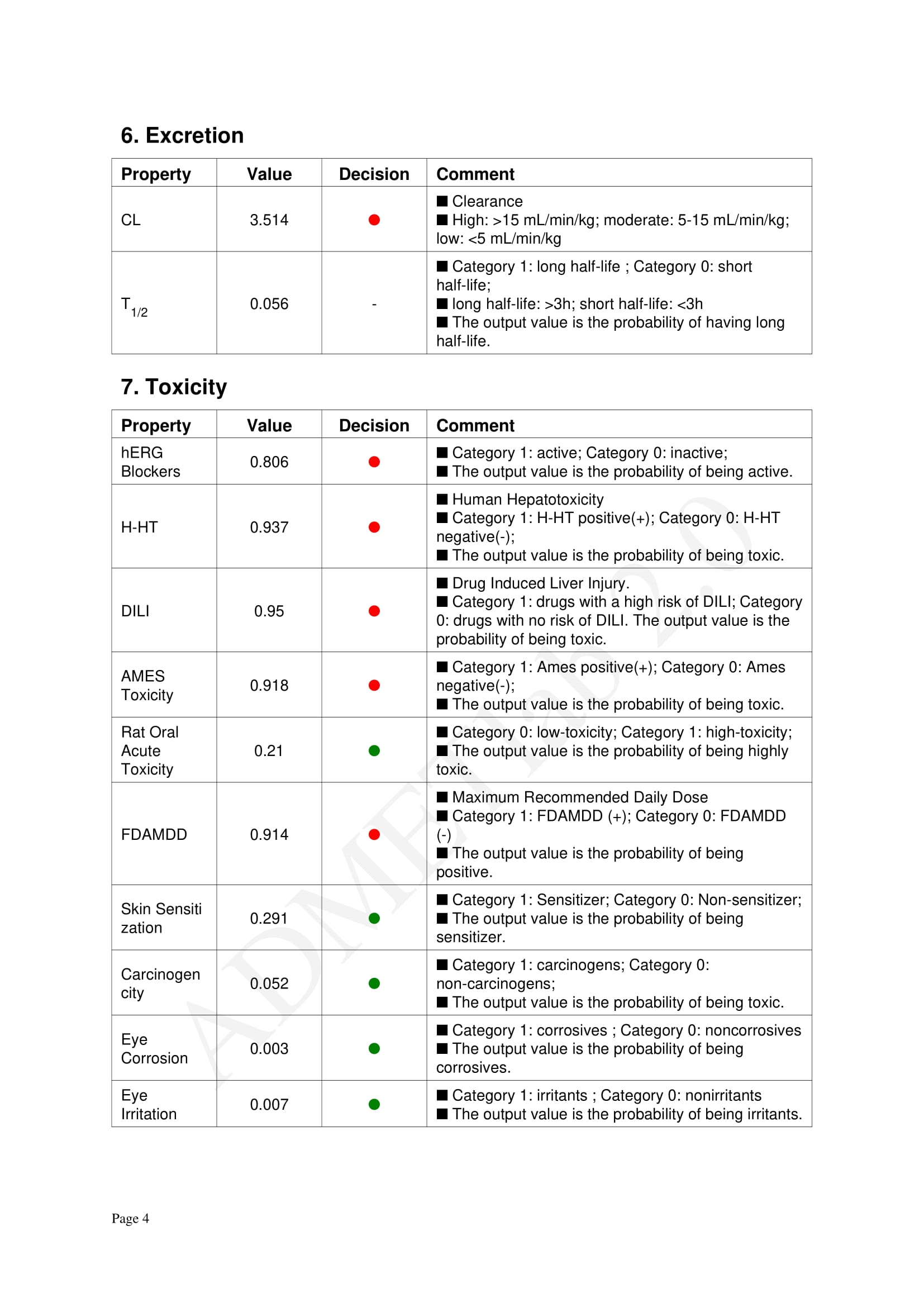

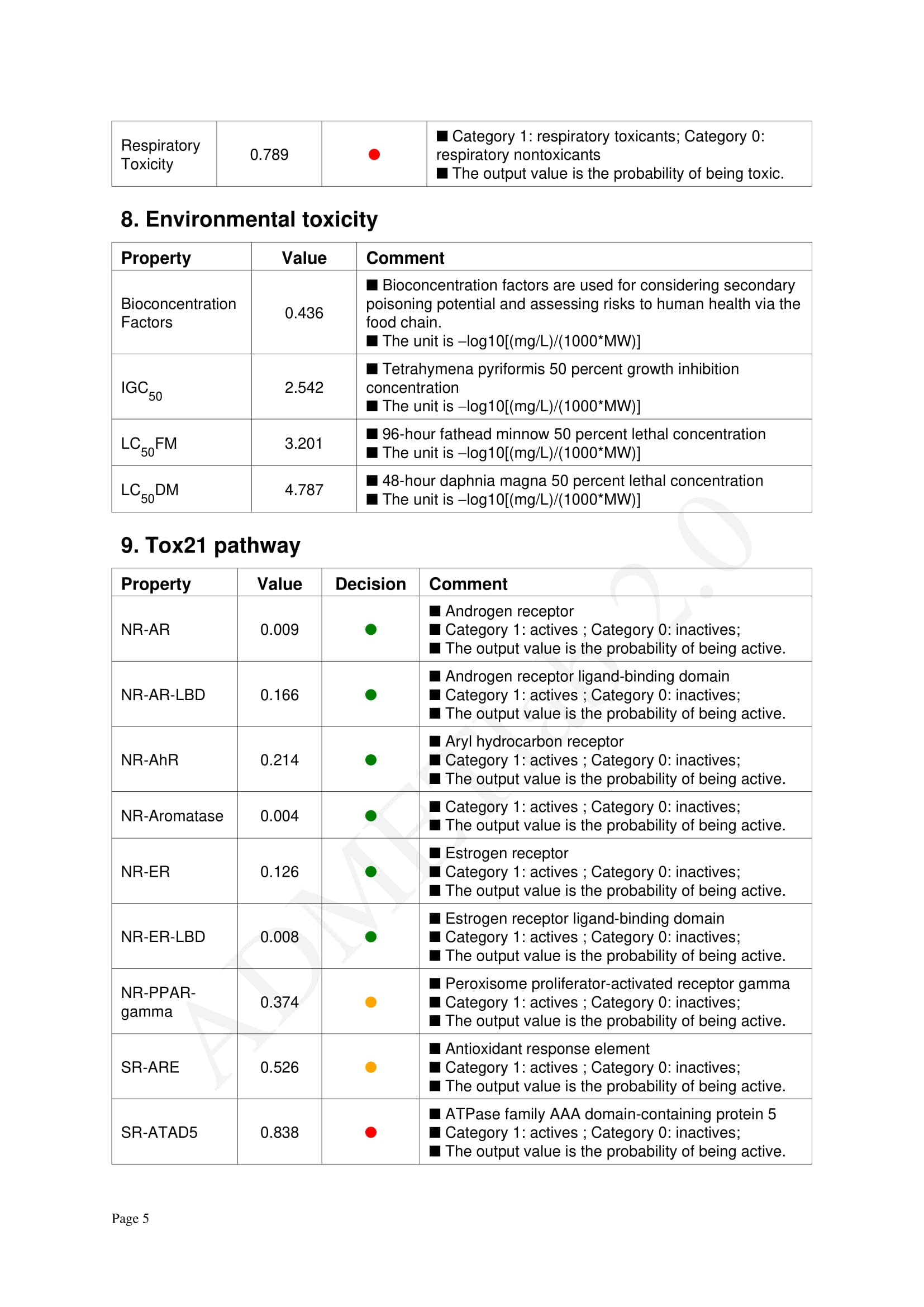

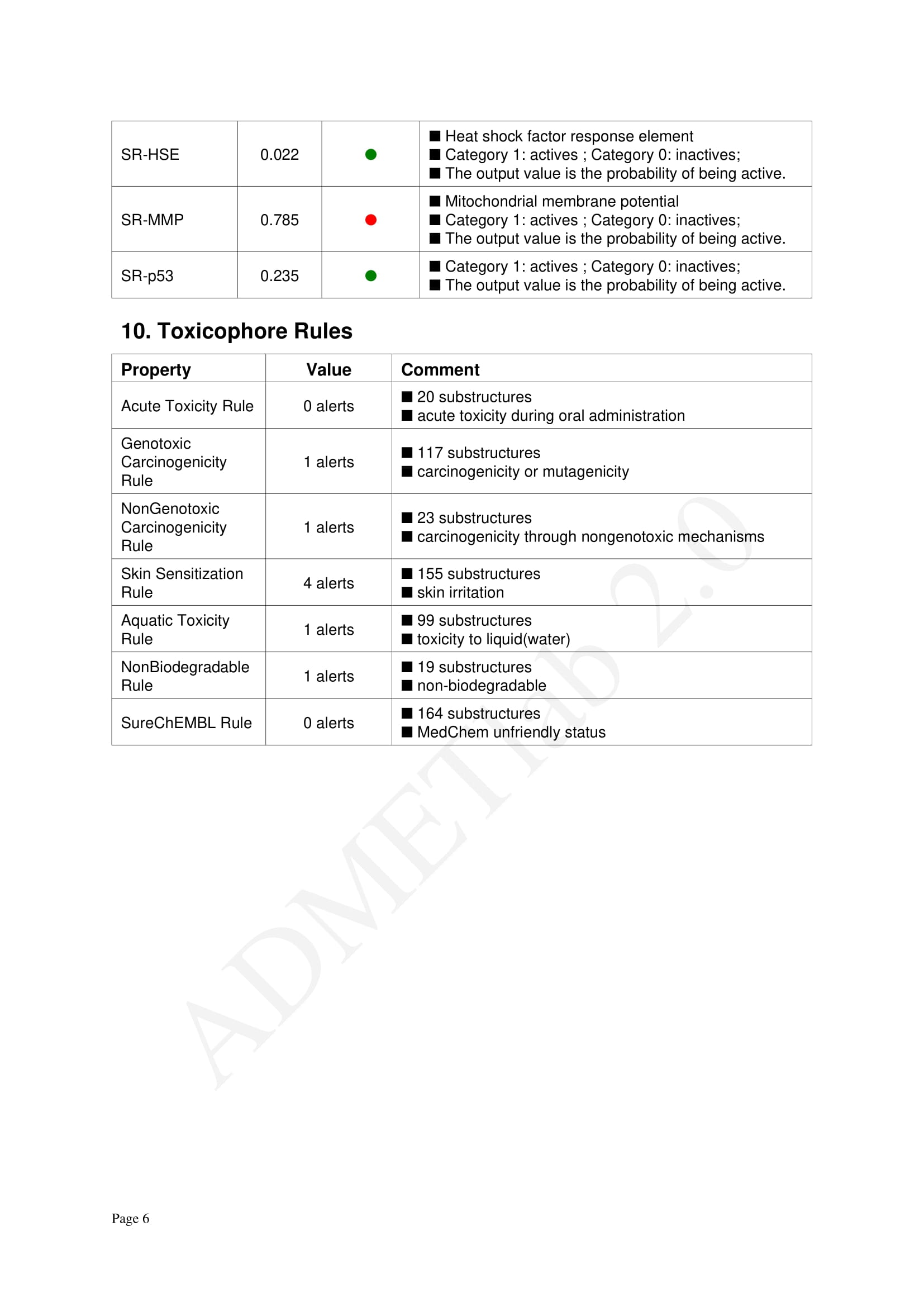


**Danusertib**
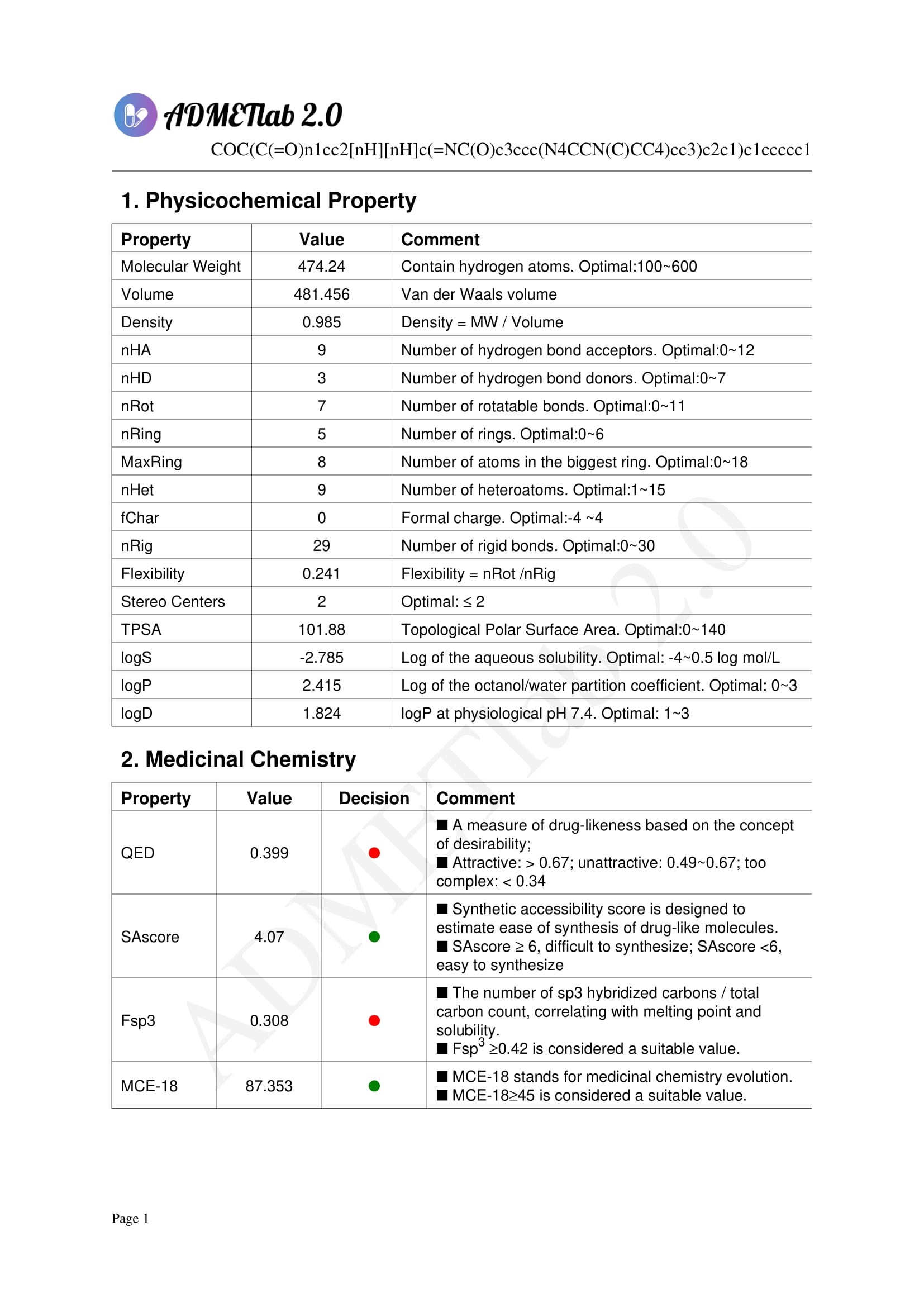

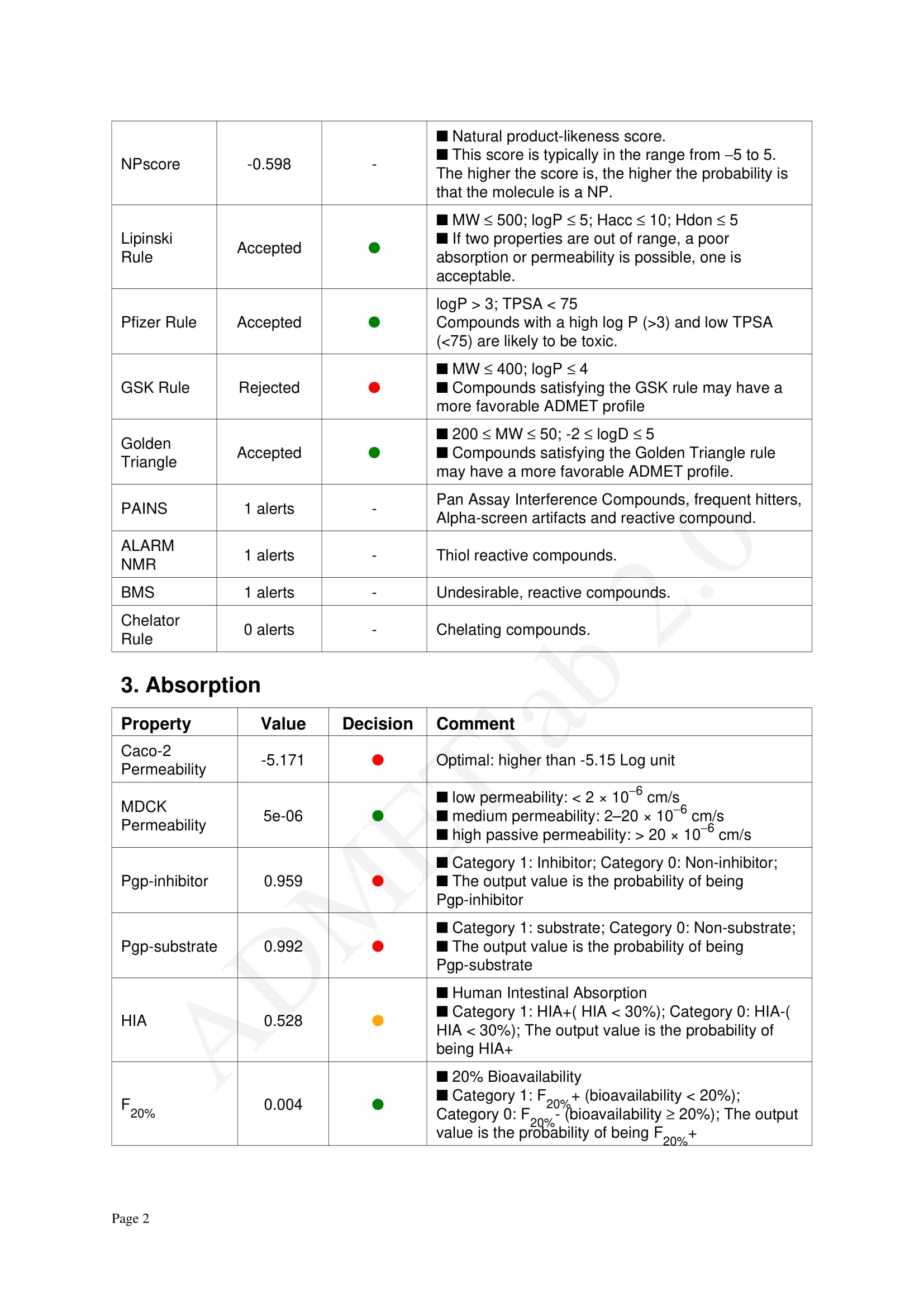

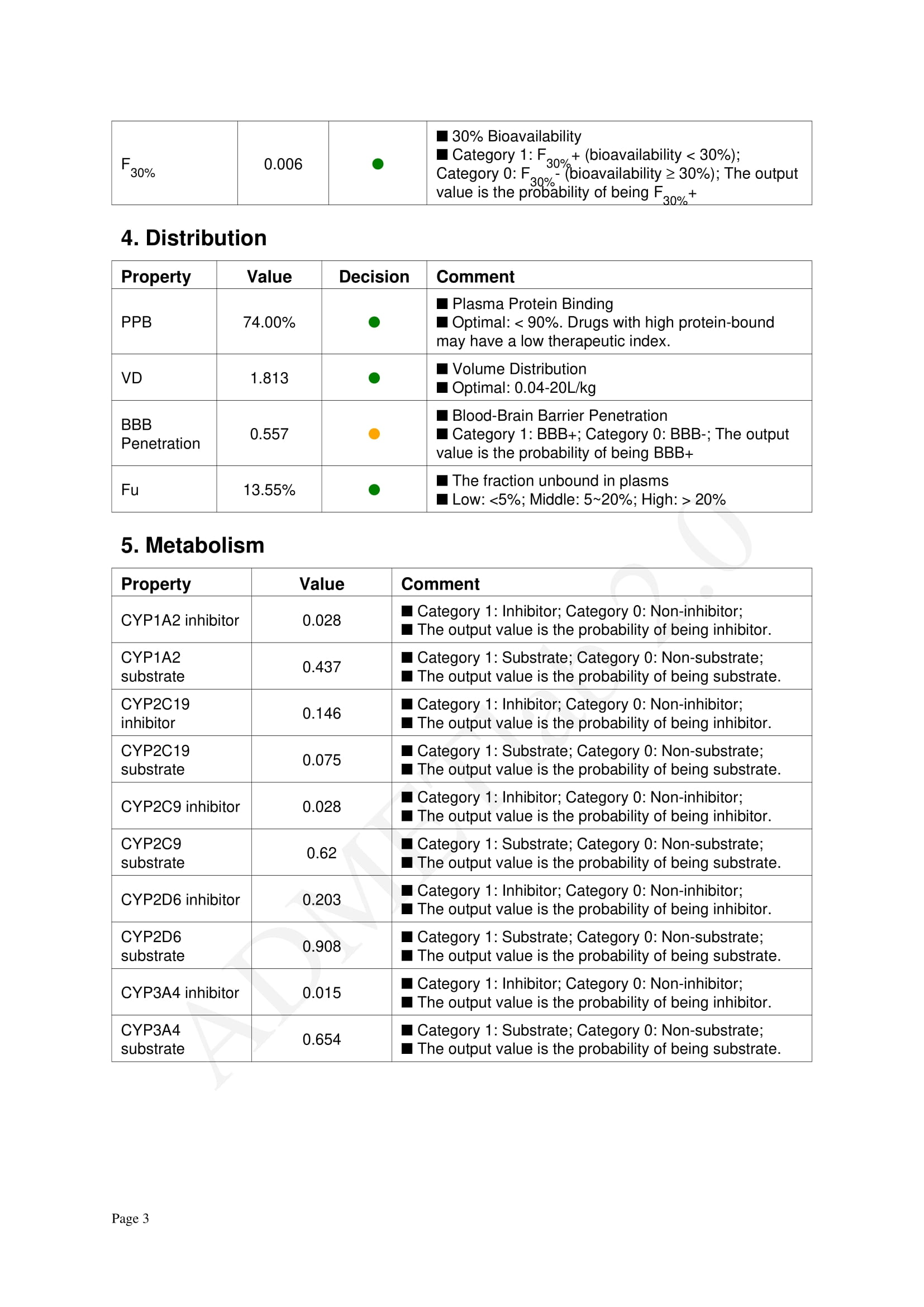

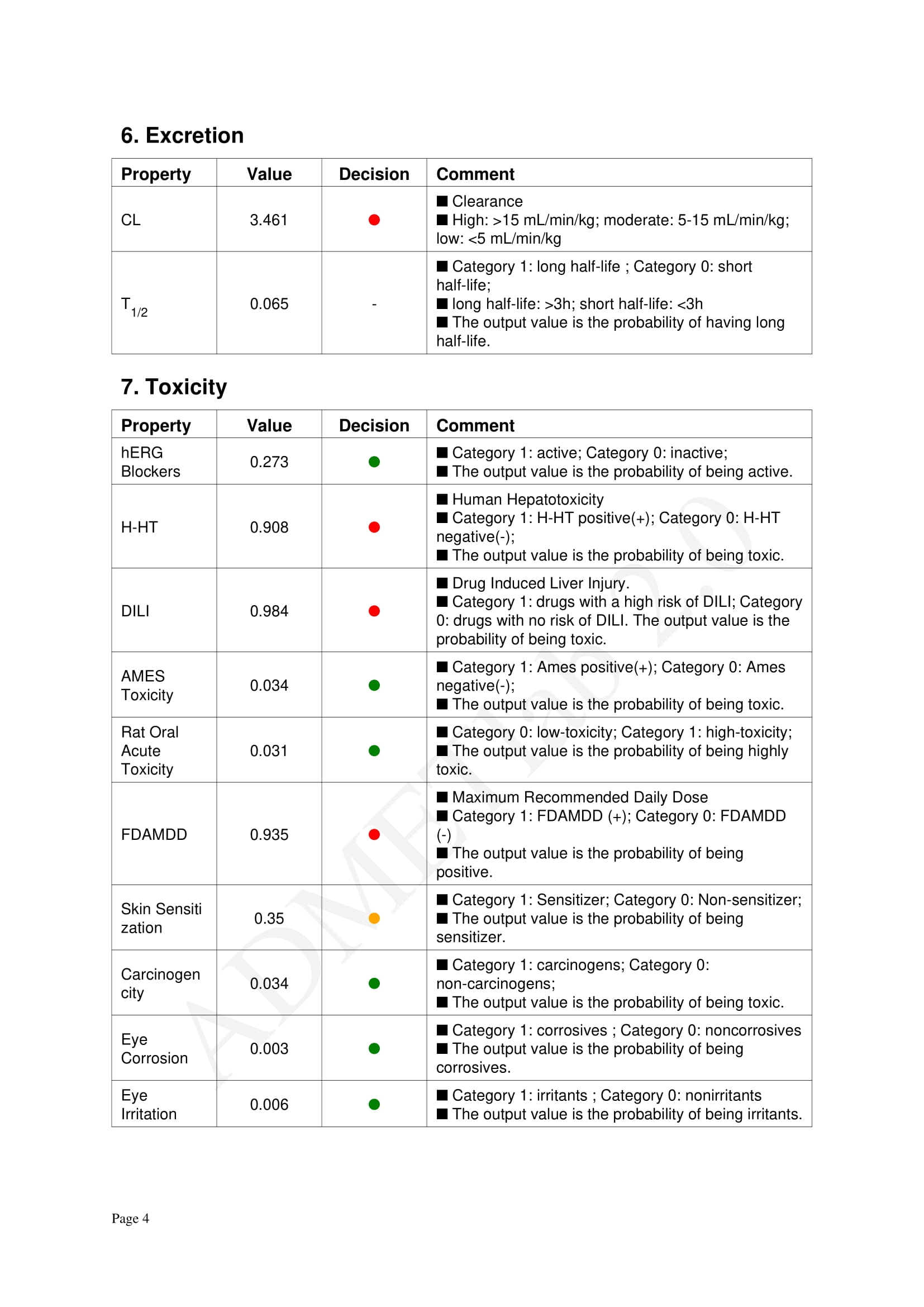

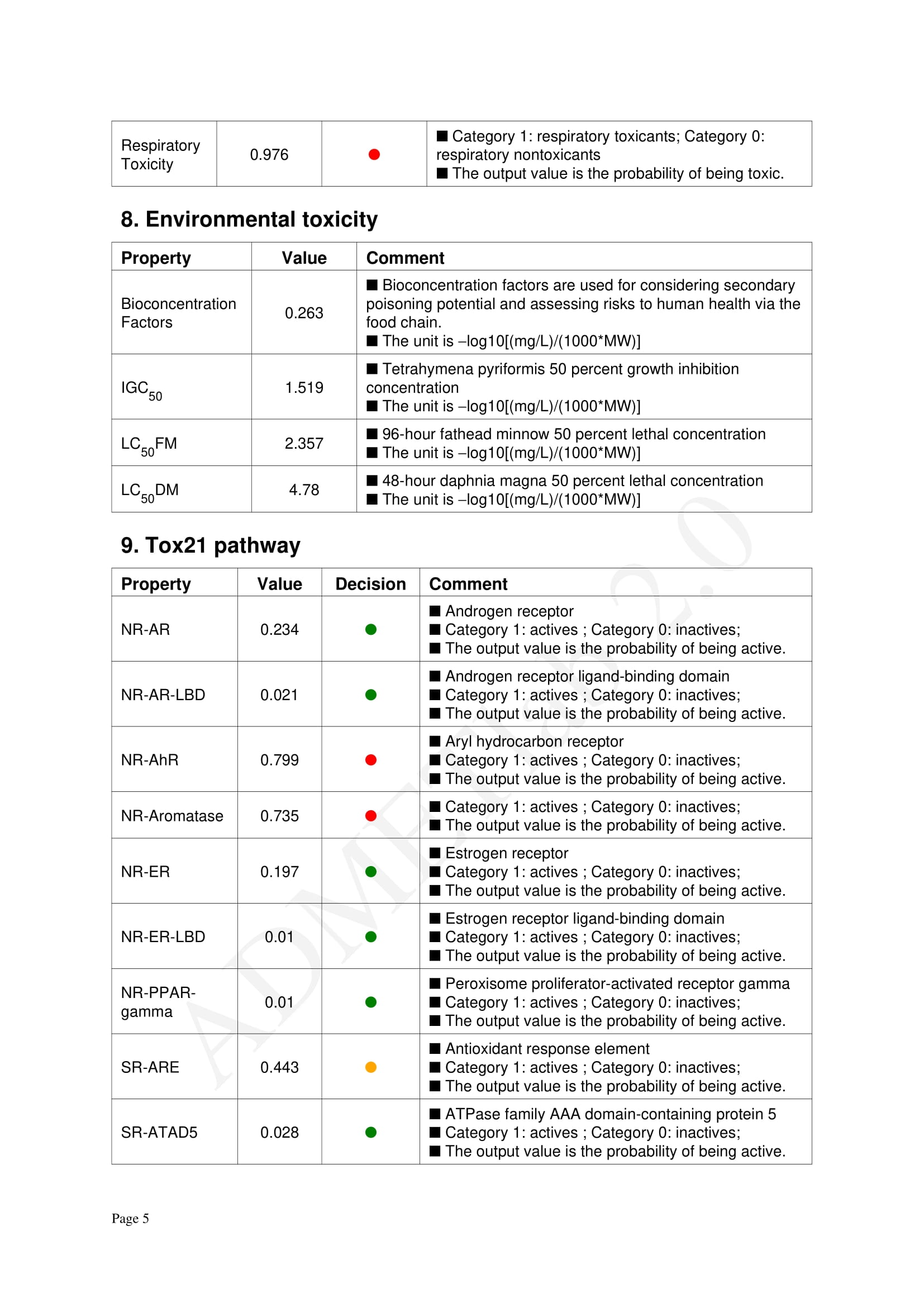

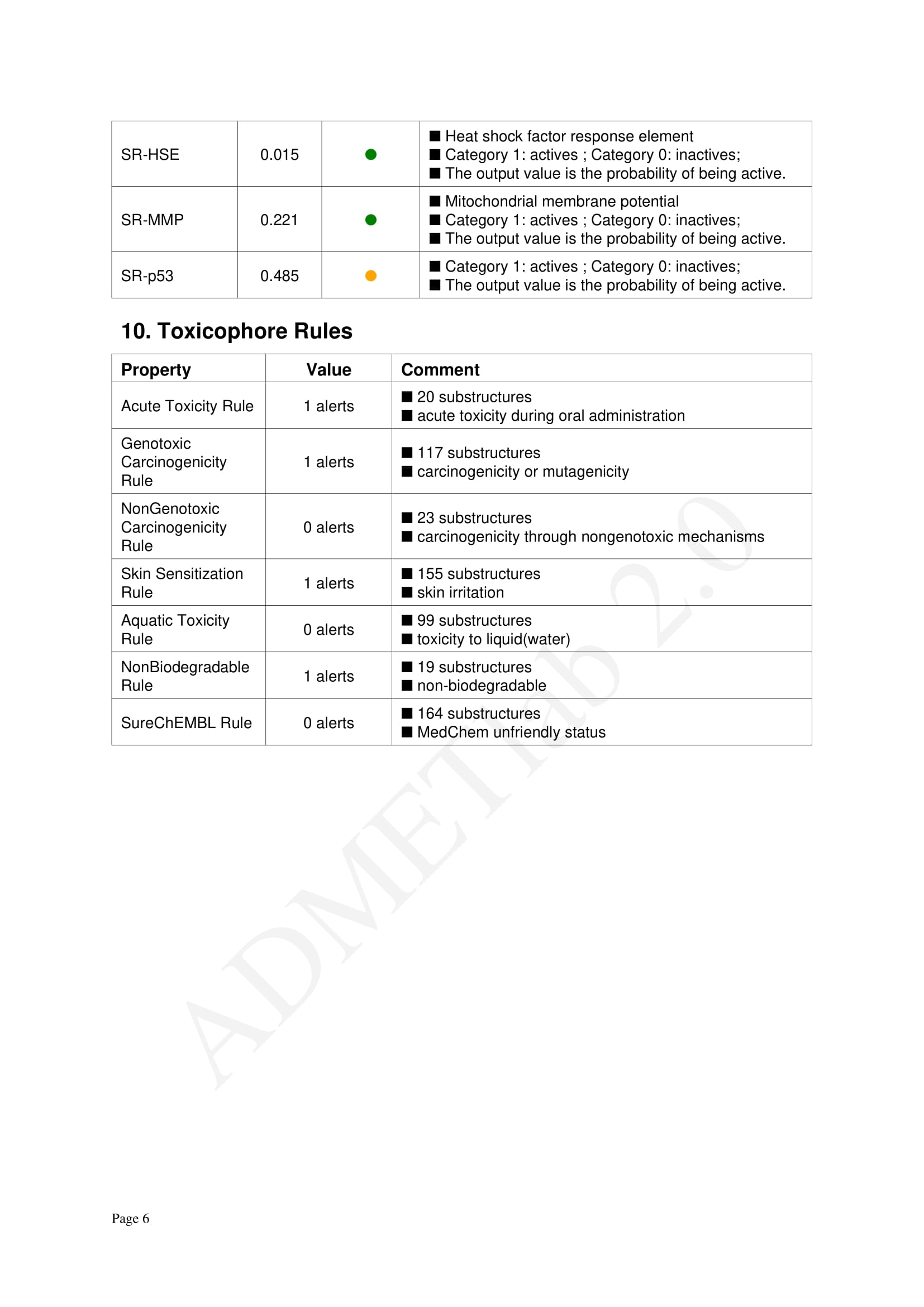


**Nintedanib**
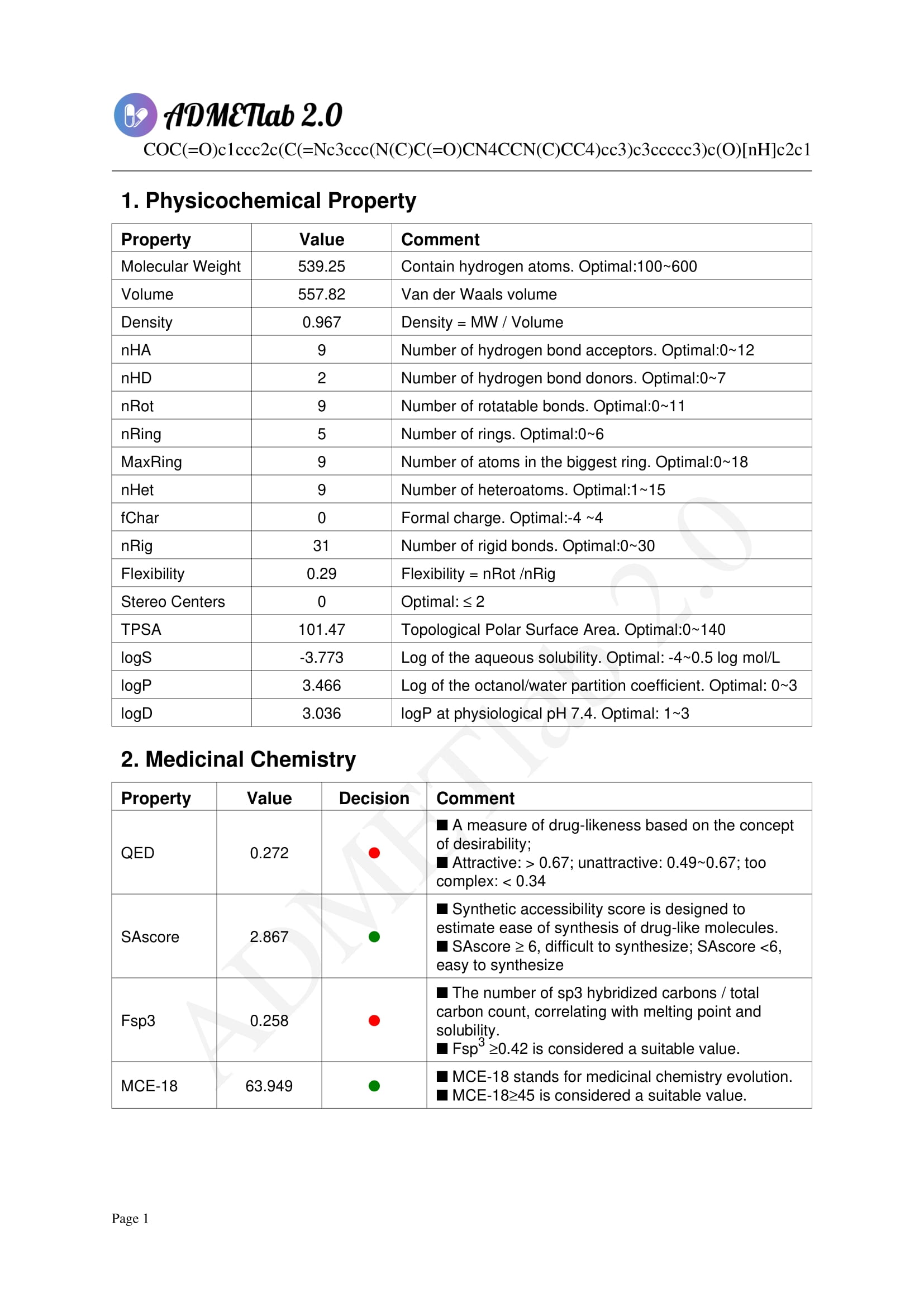

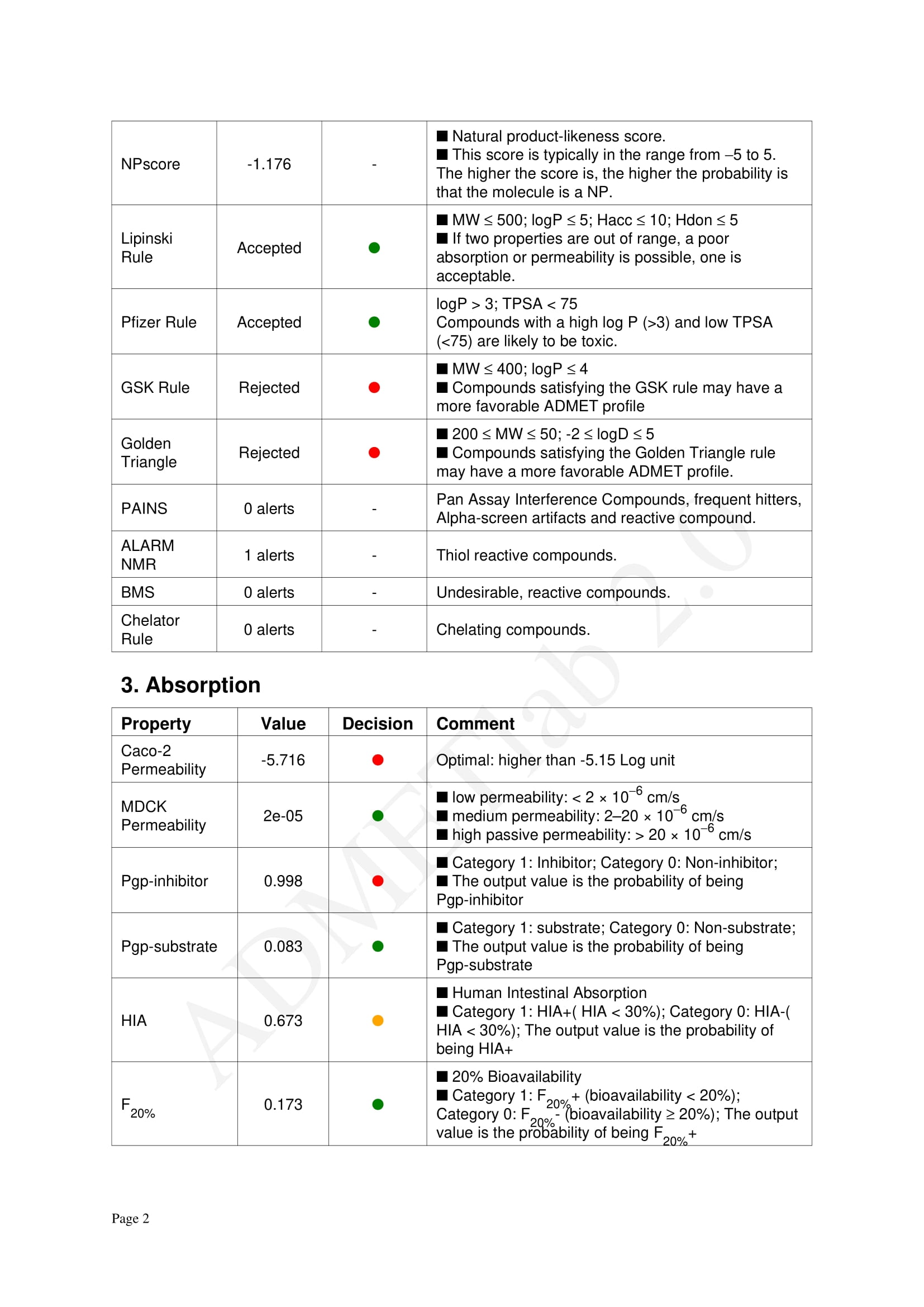

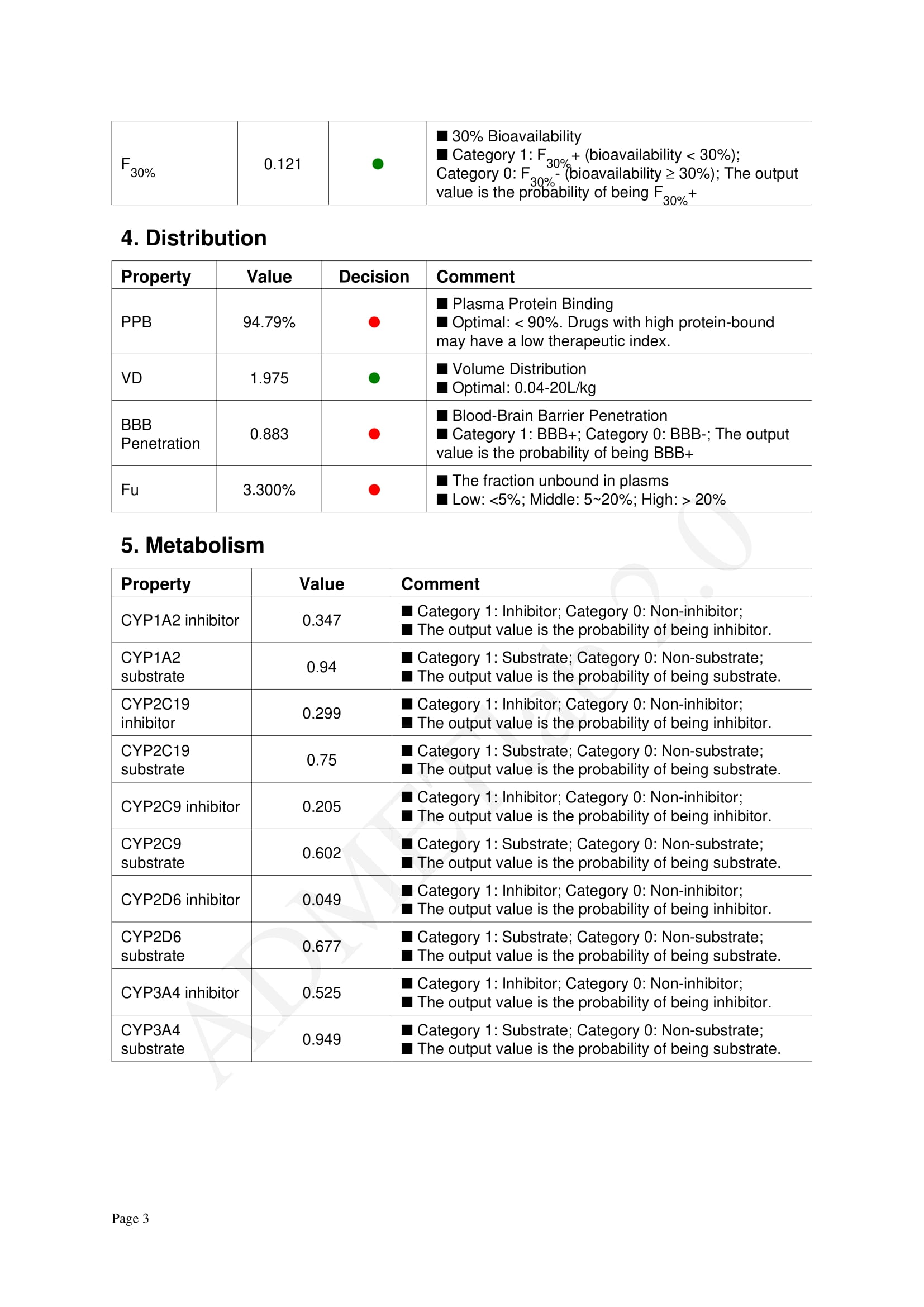

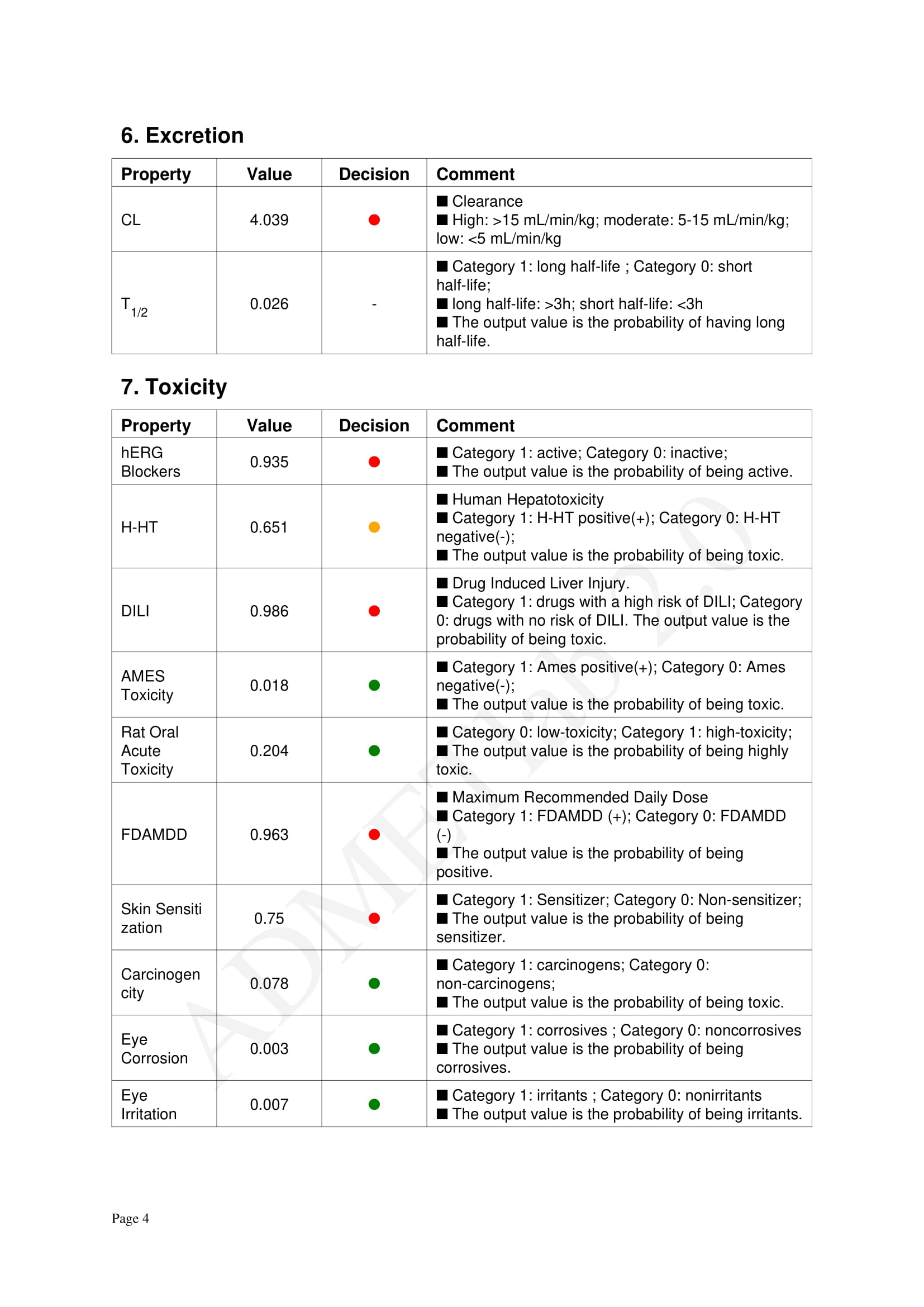

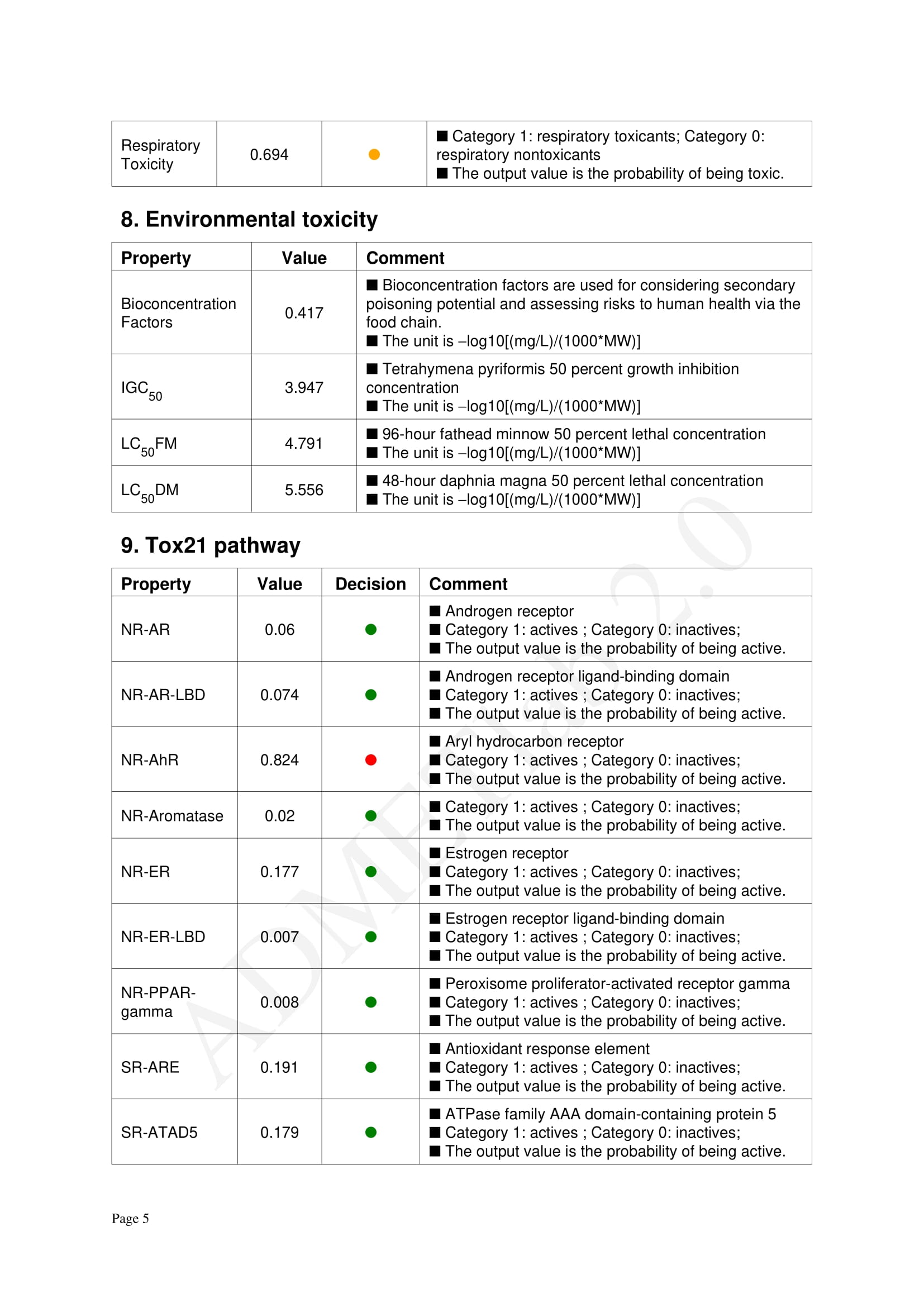

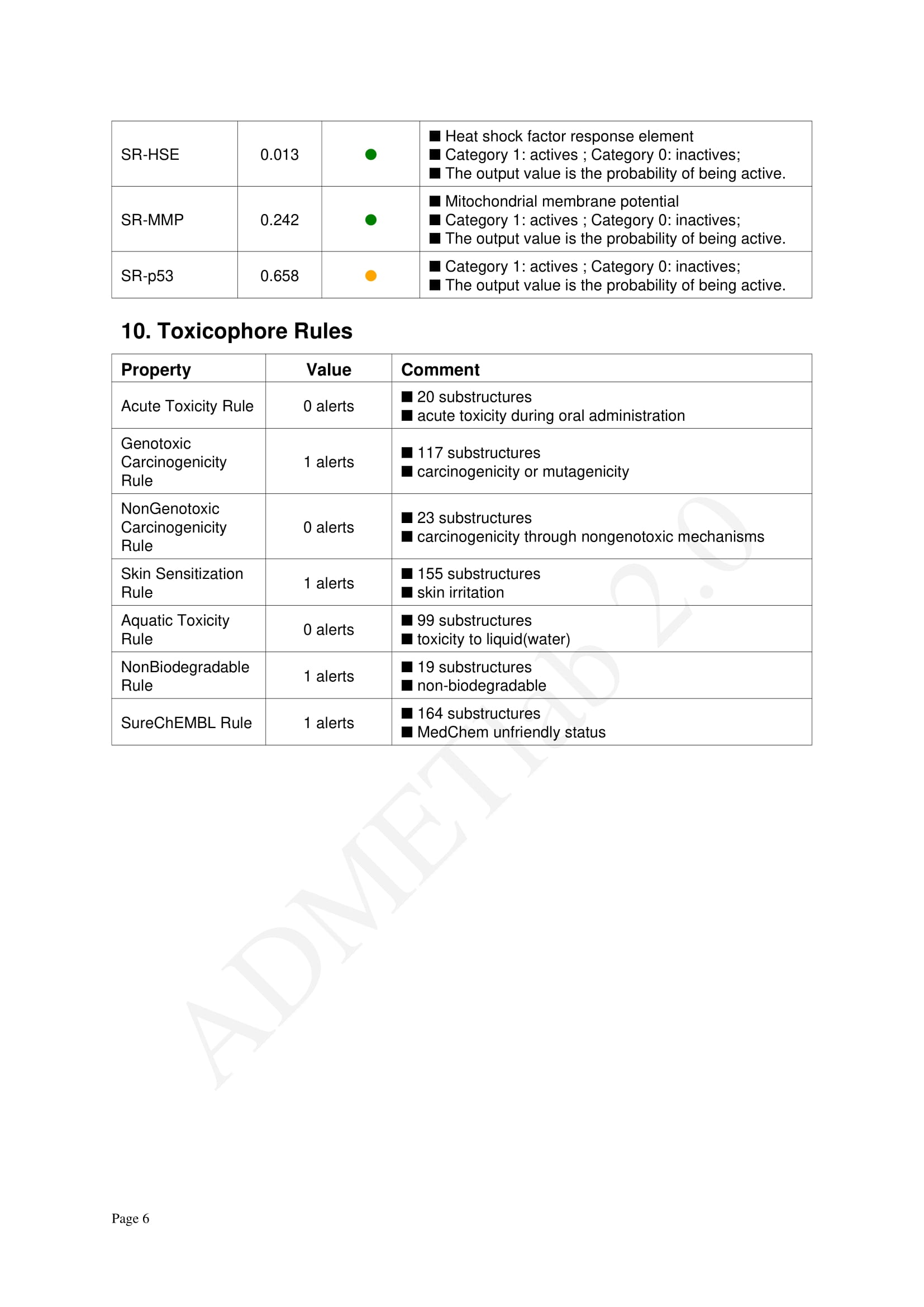


**OSU-03012**
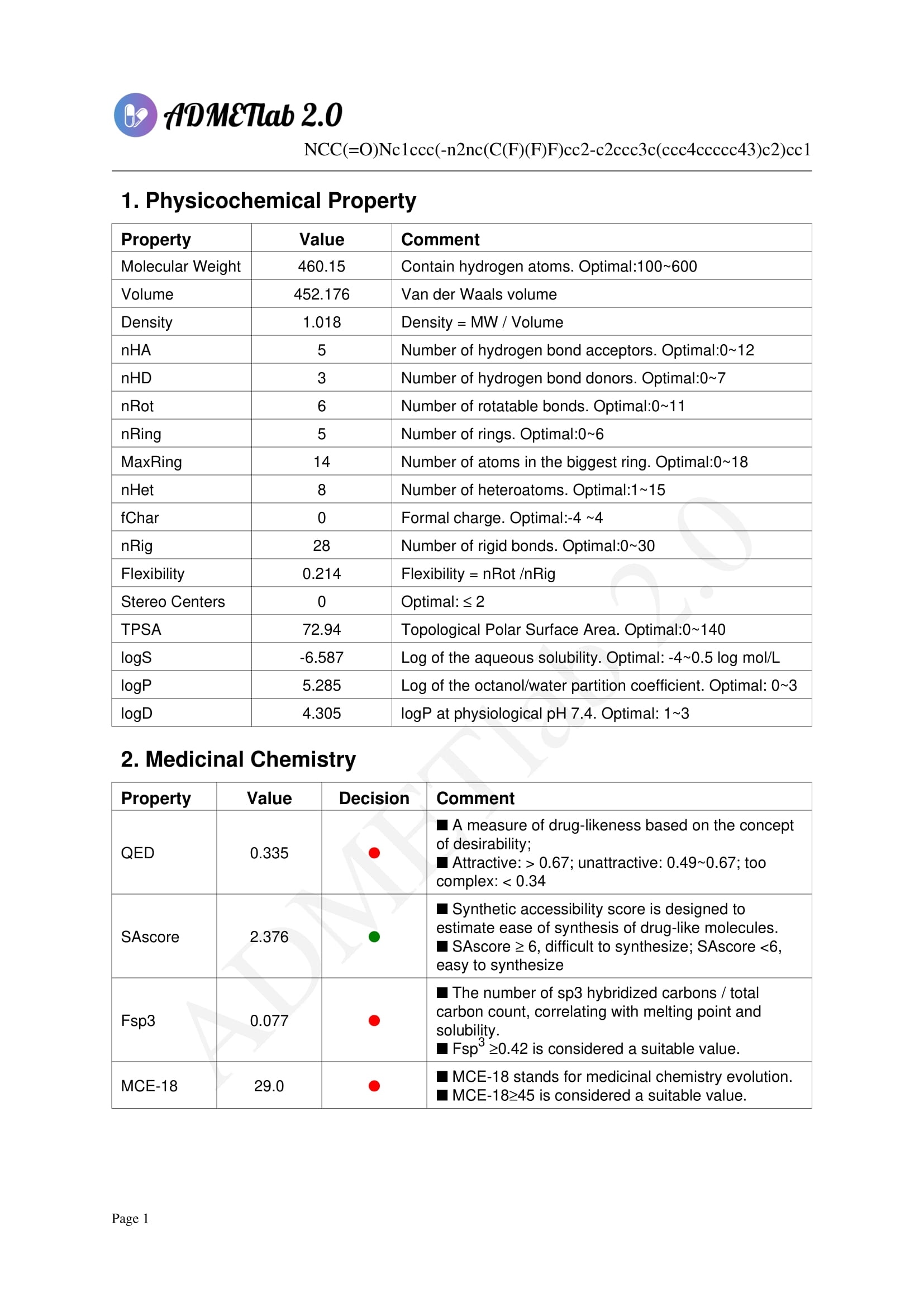

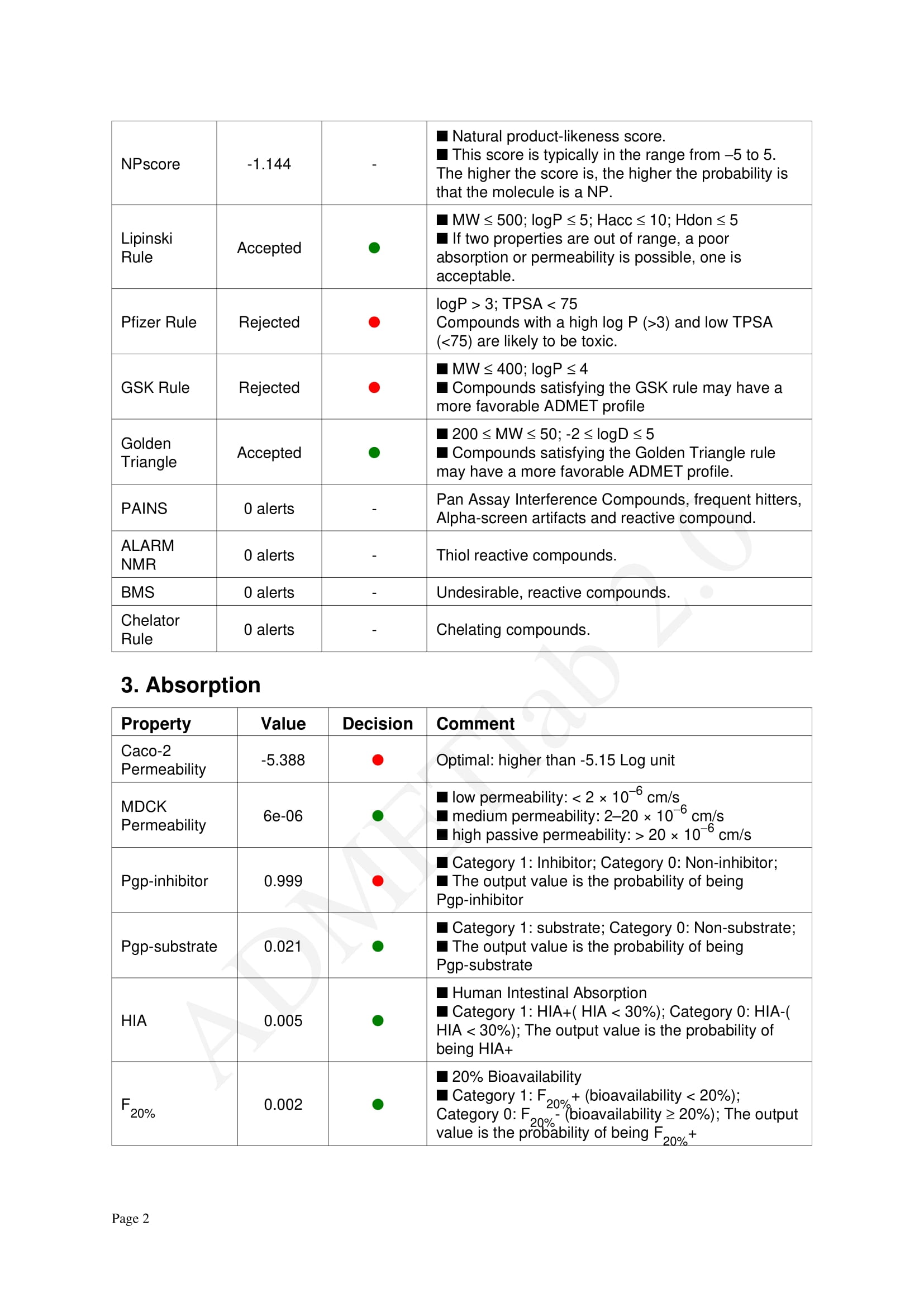

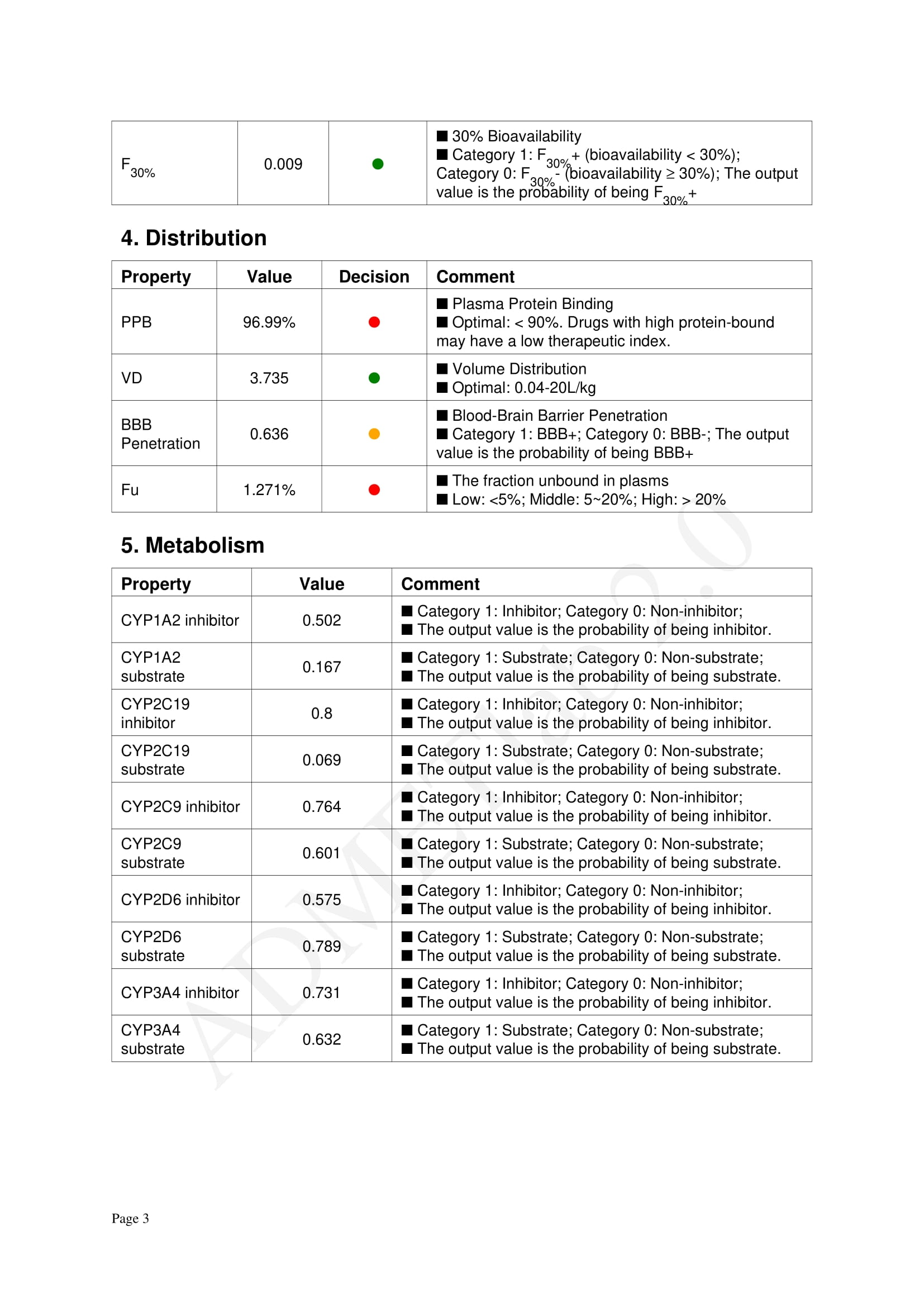

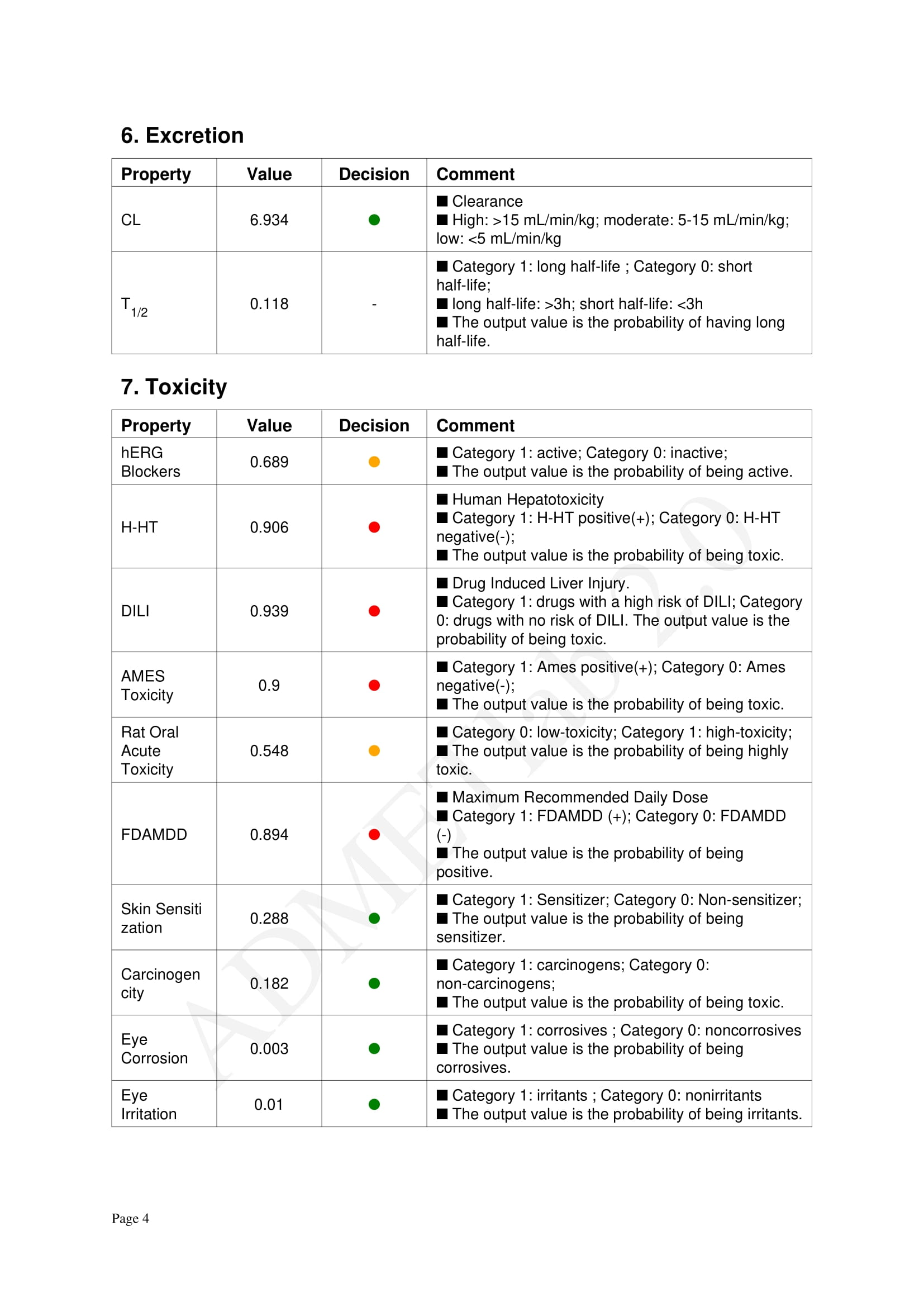

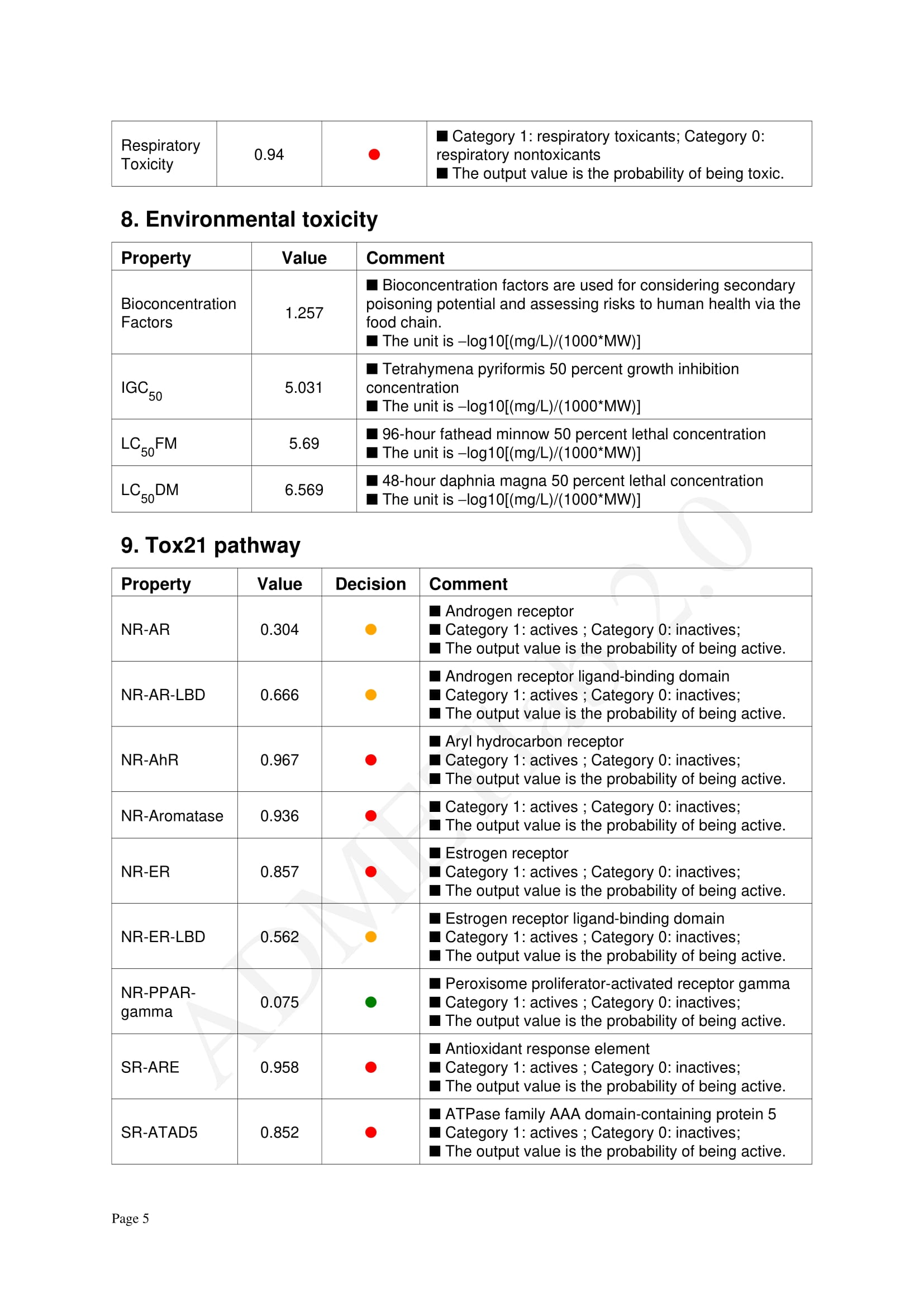

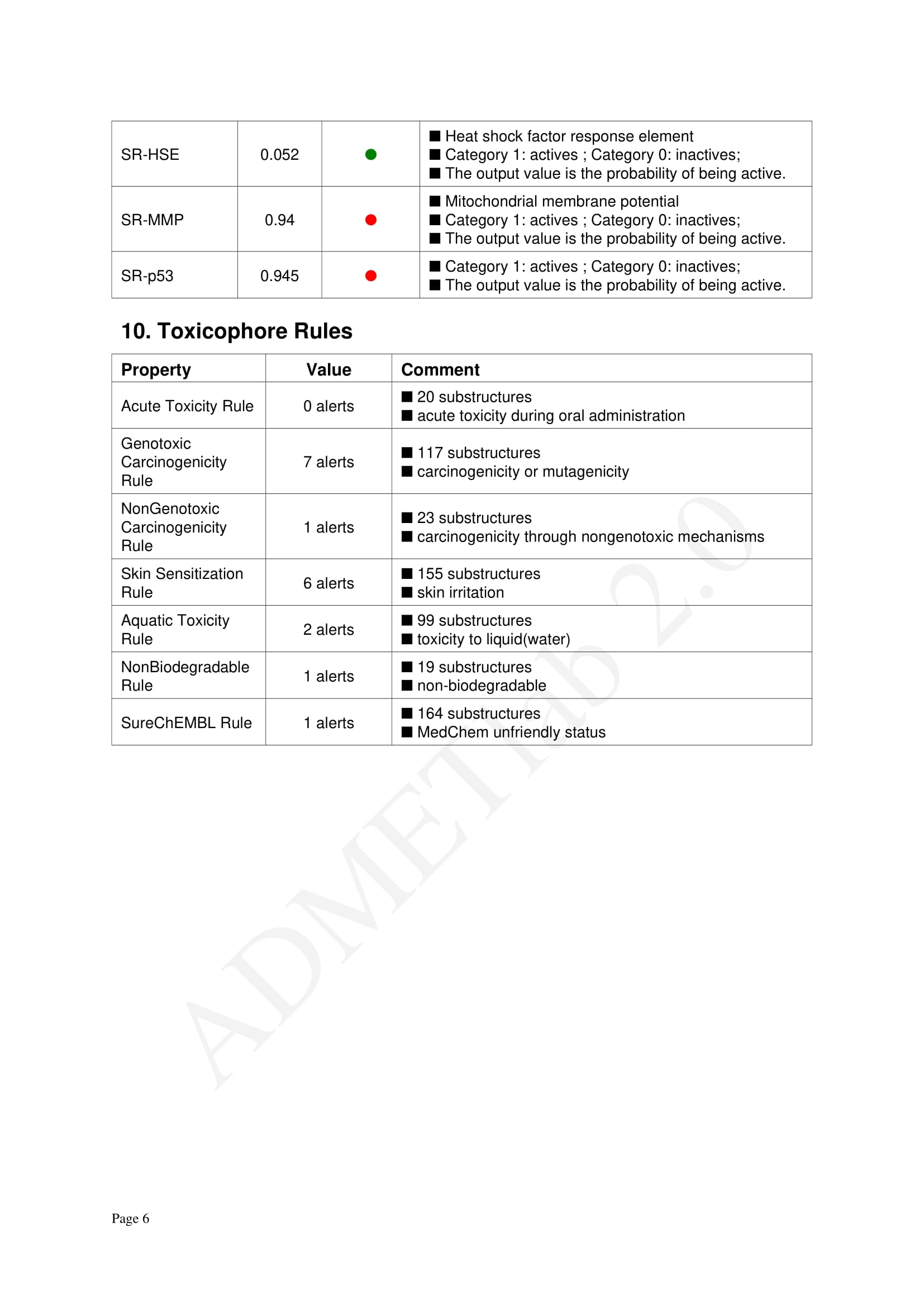


**SNS-314**
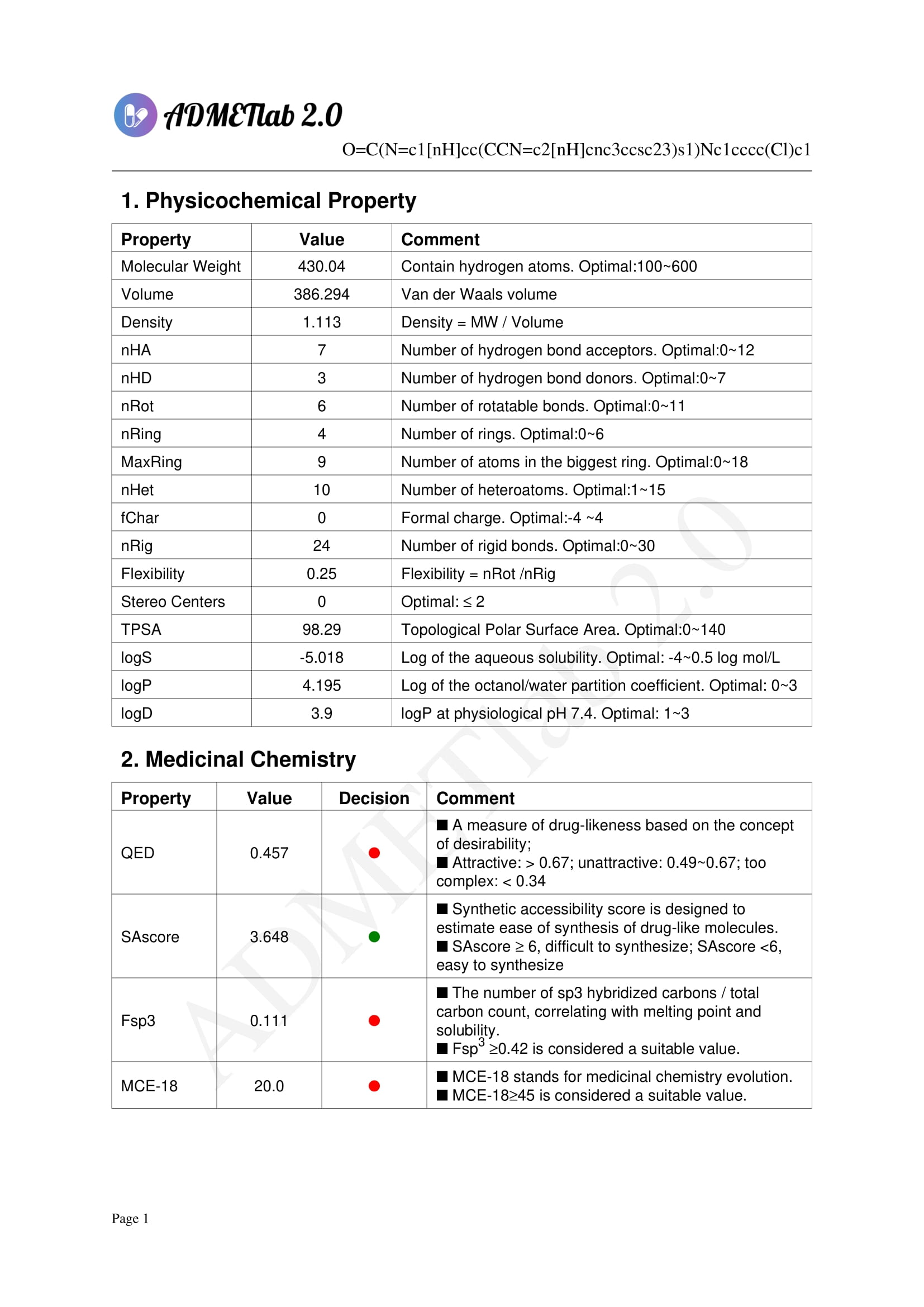

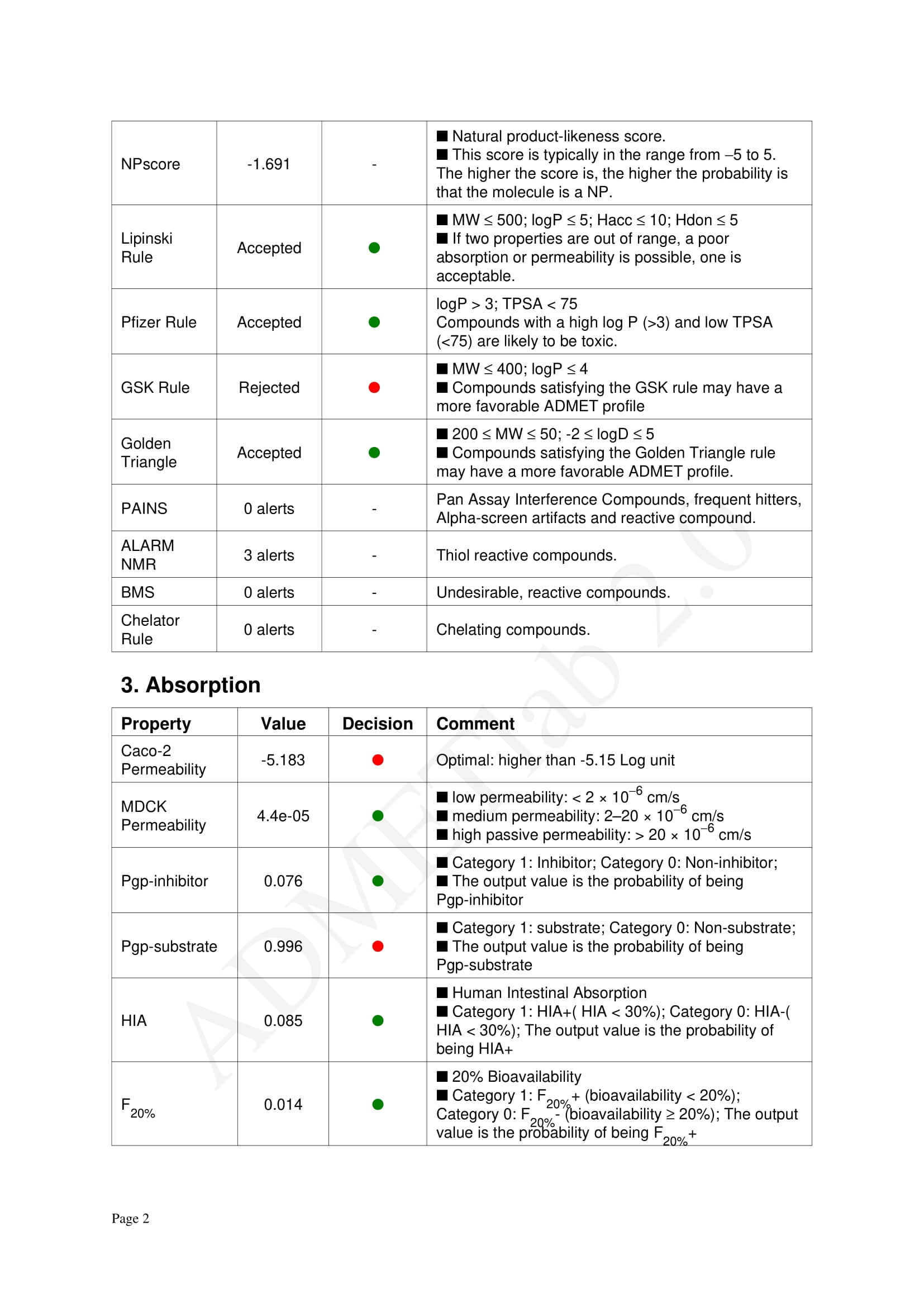

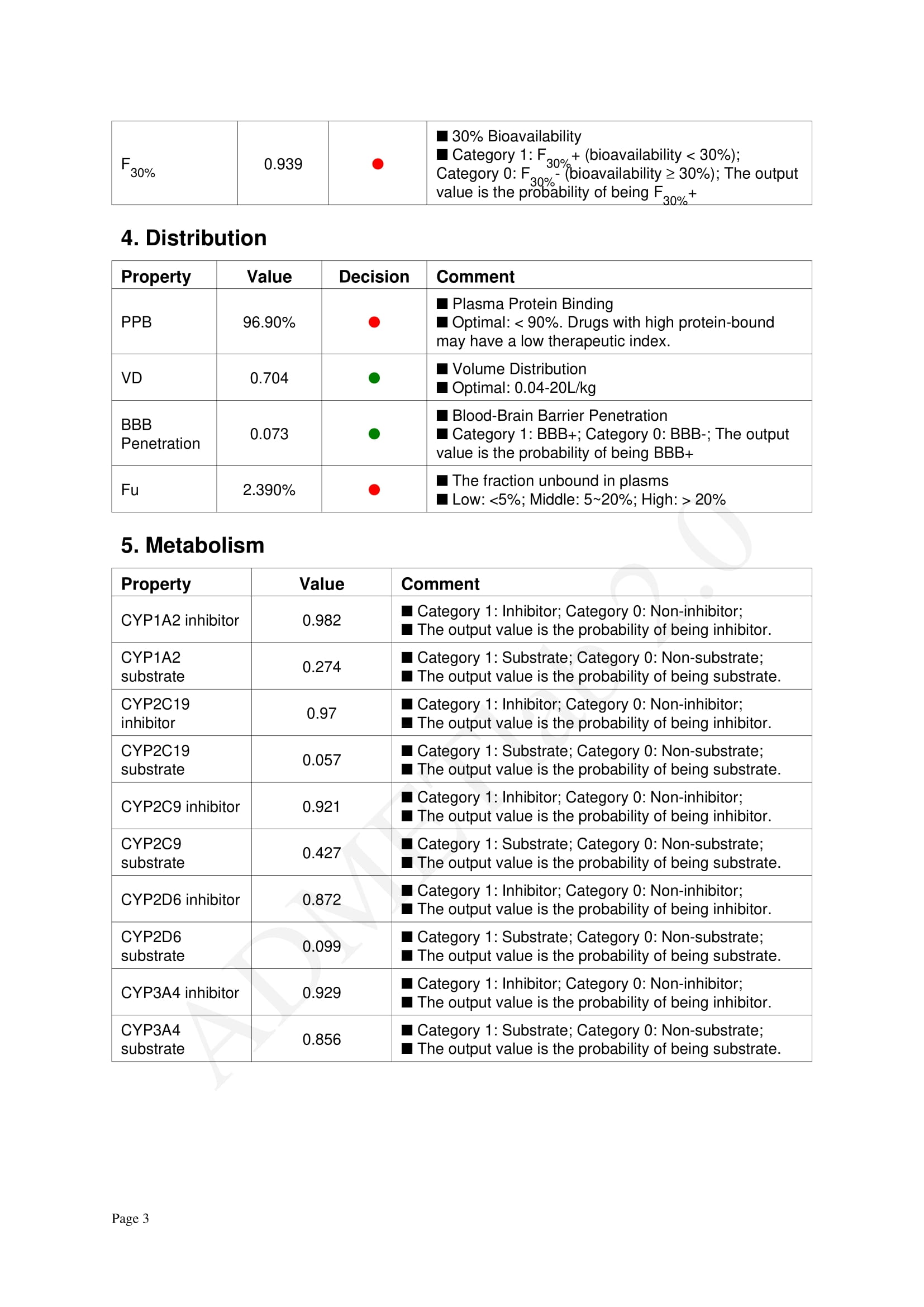

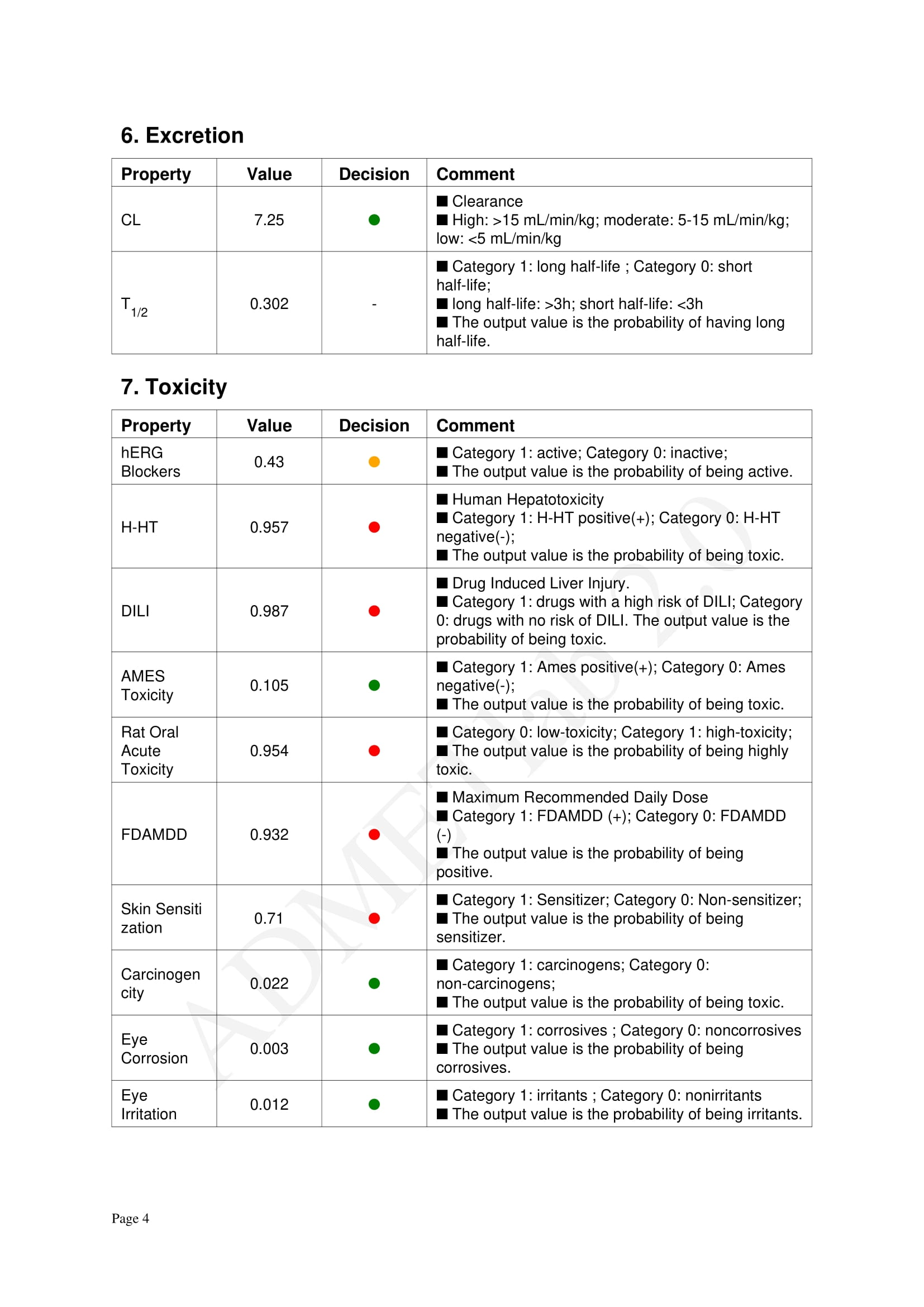

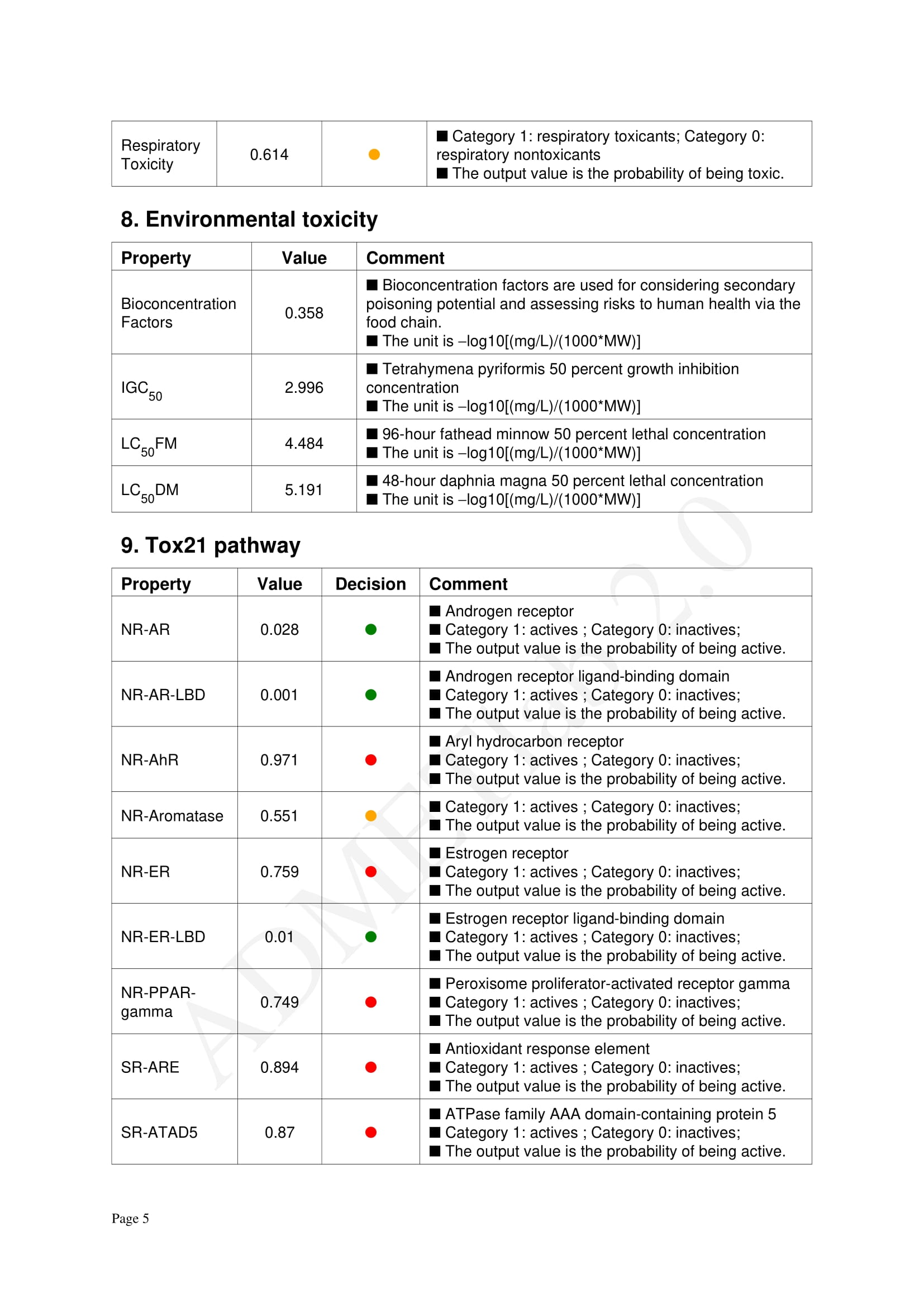

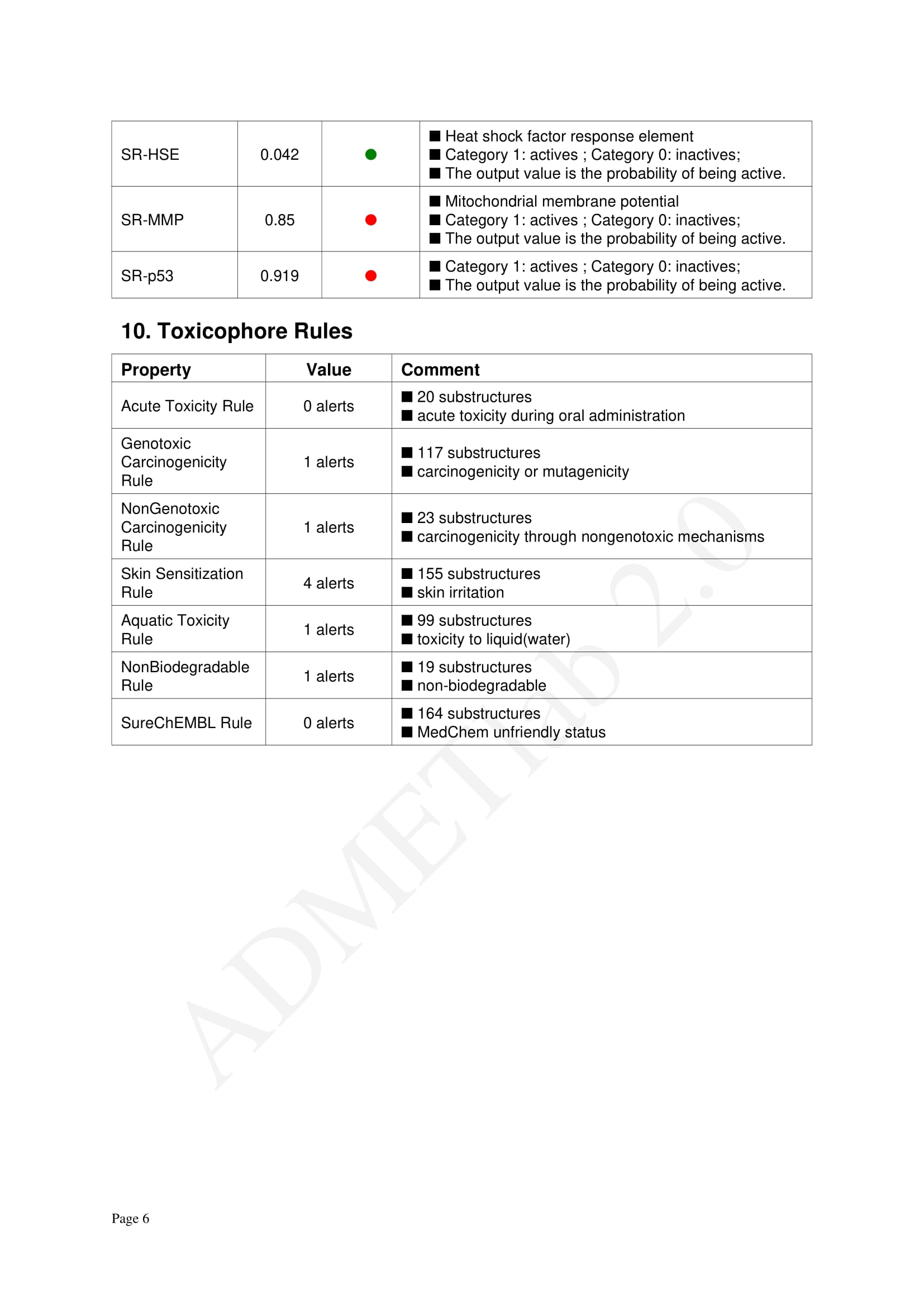


**Sorafenib**
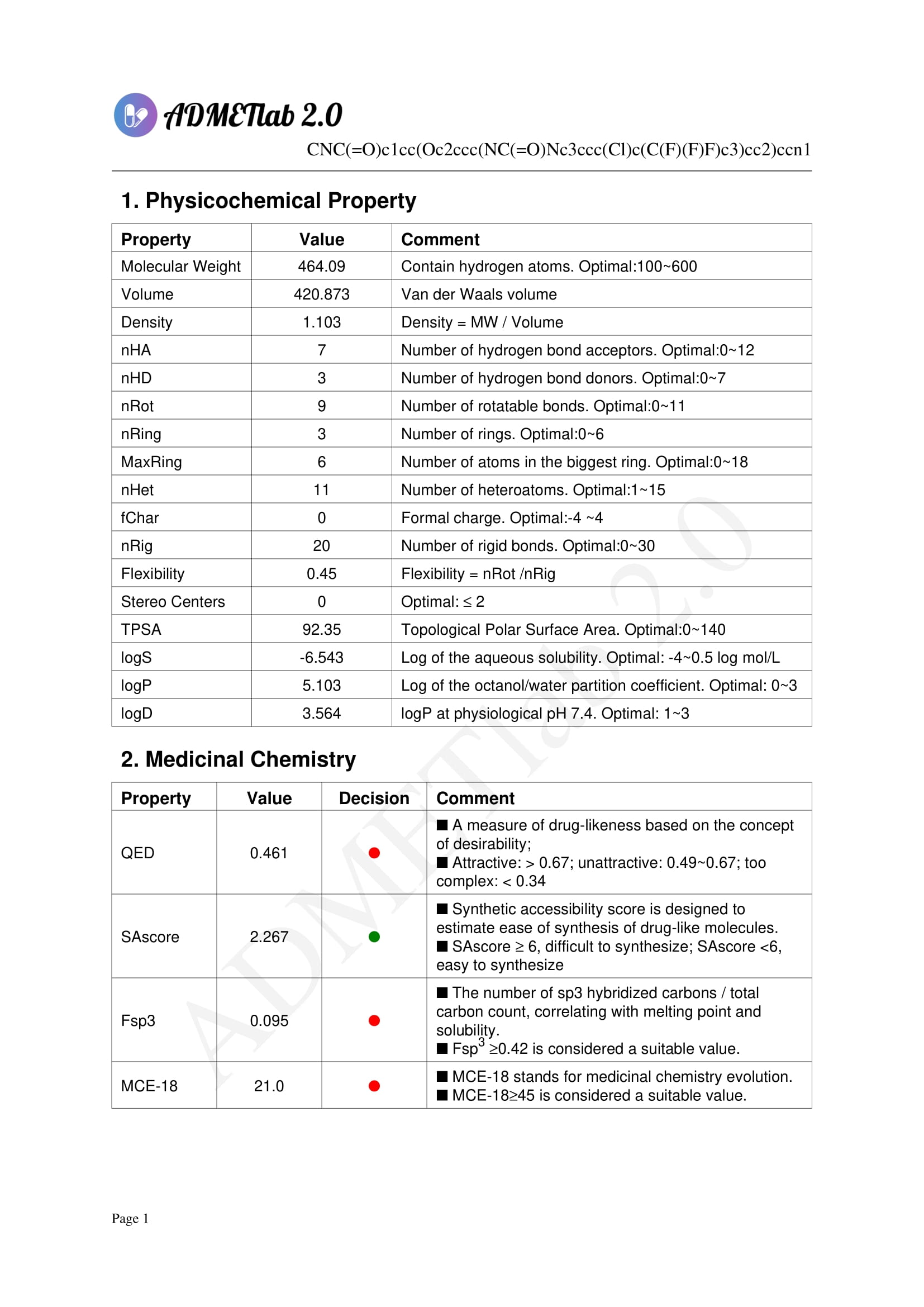

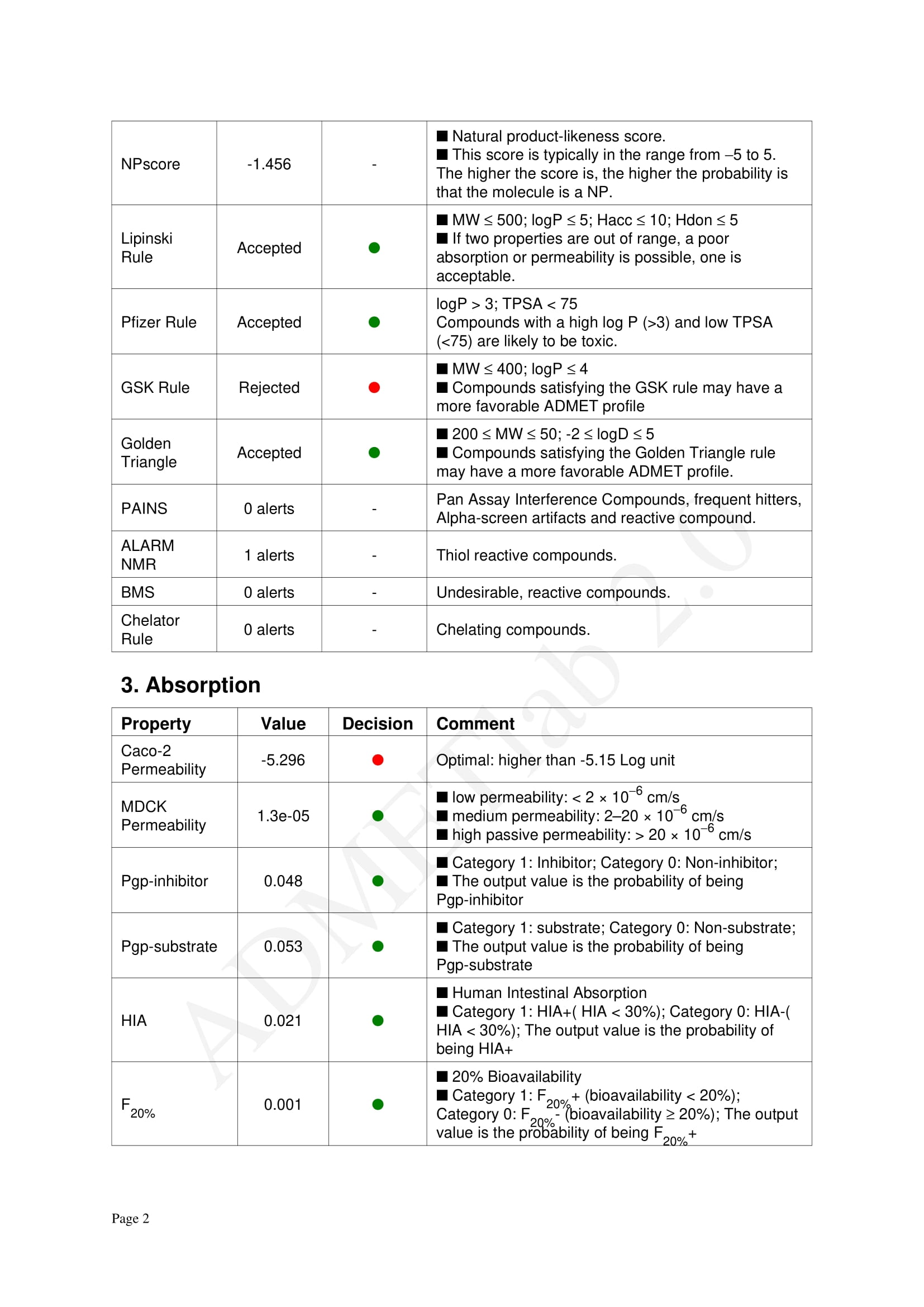

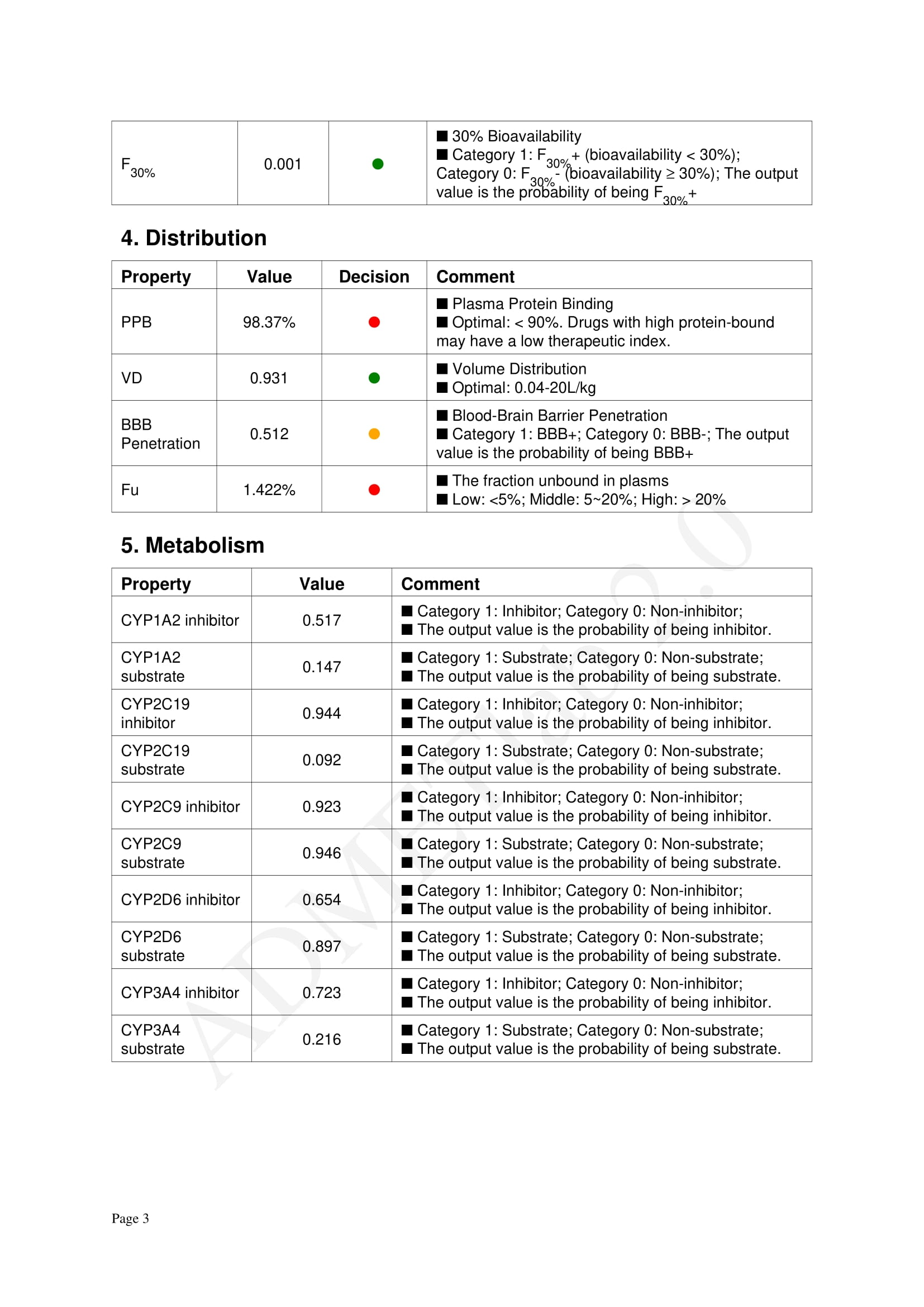

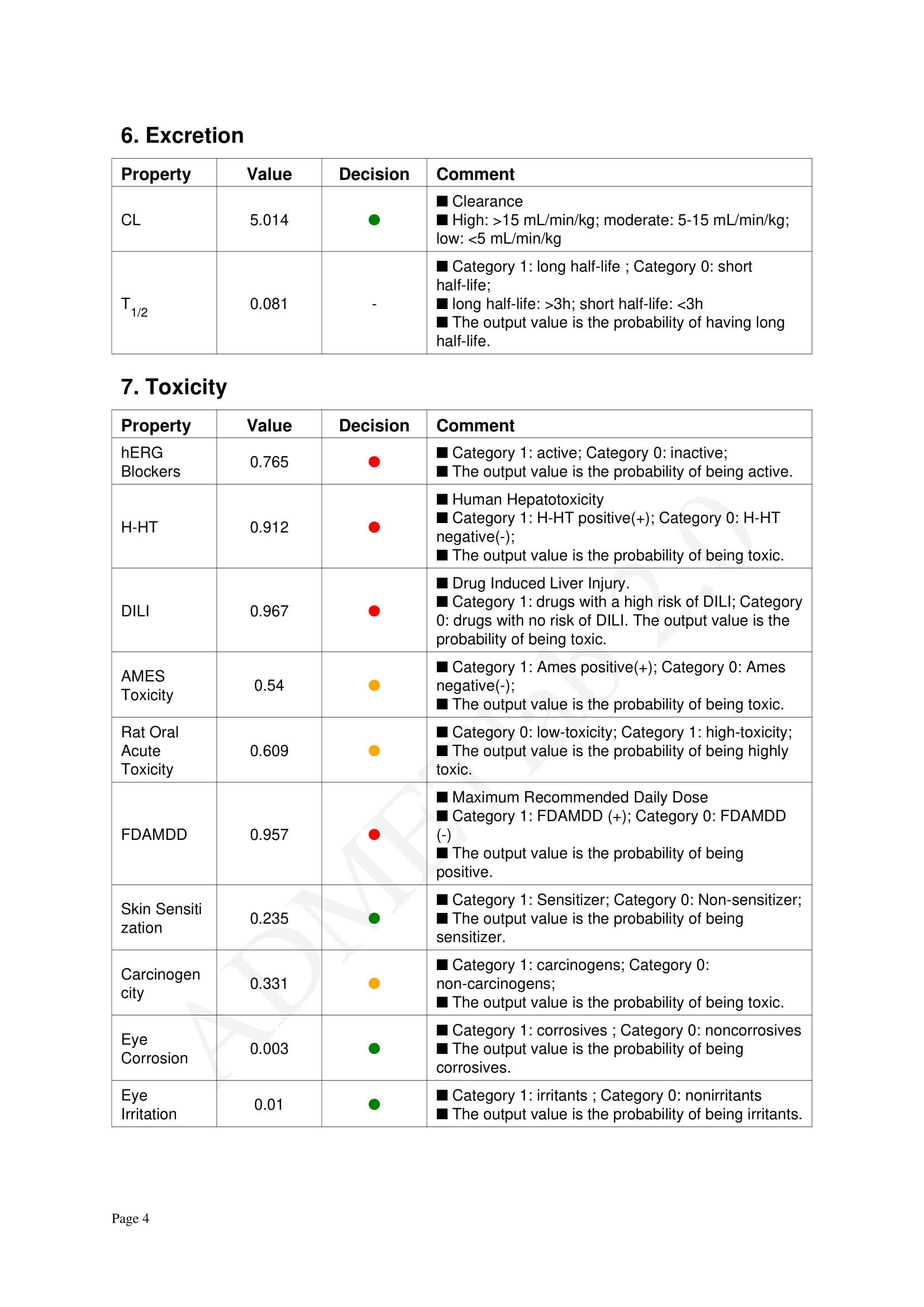

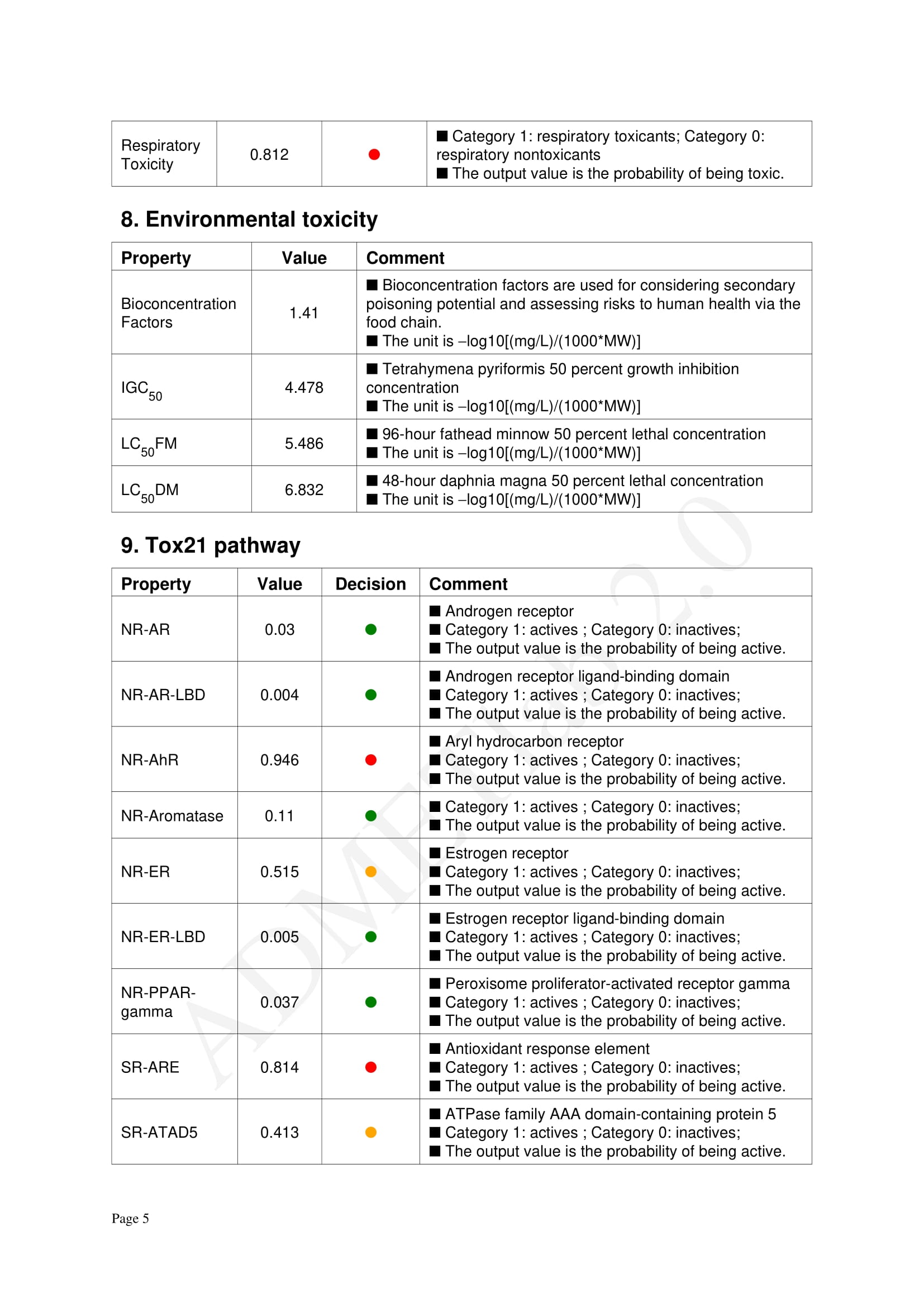

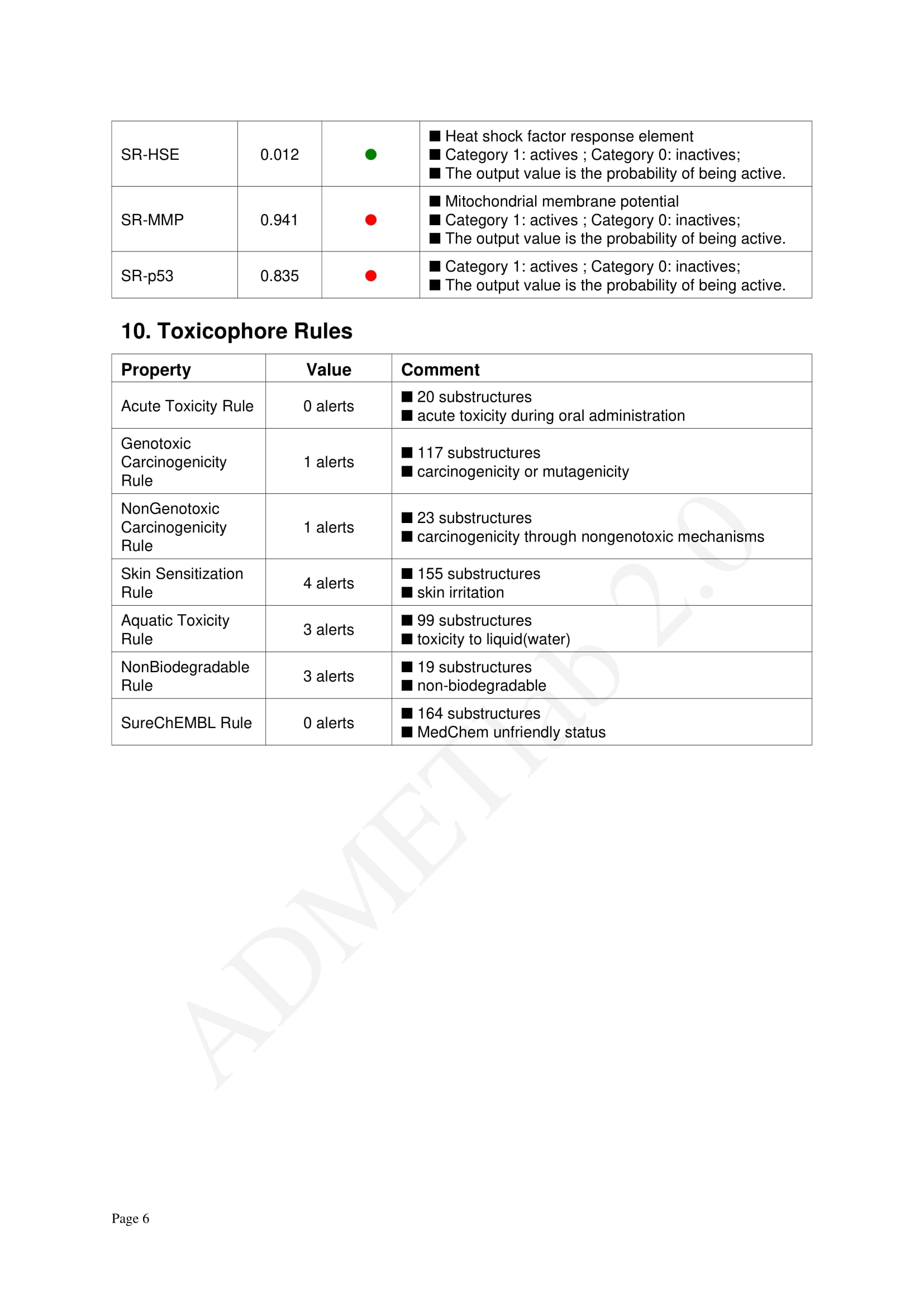


**Talazoparib**
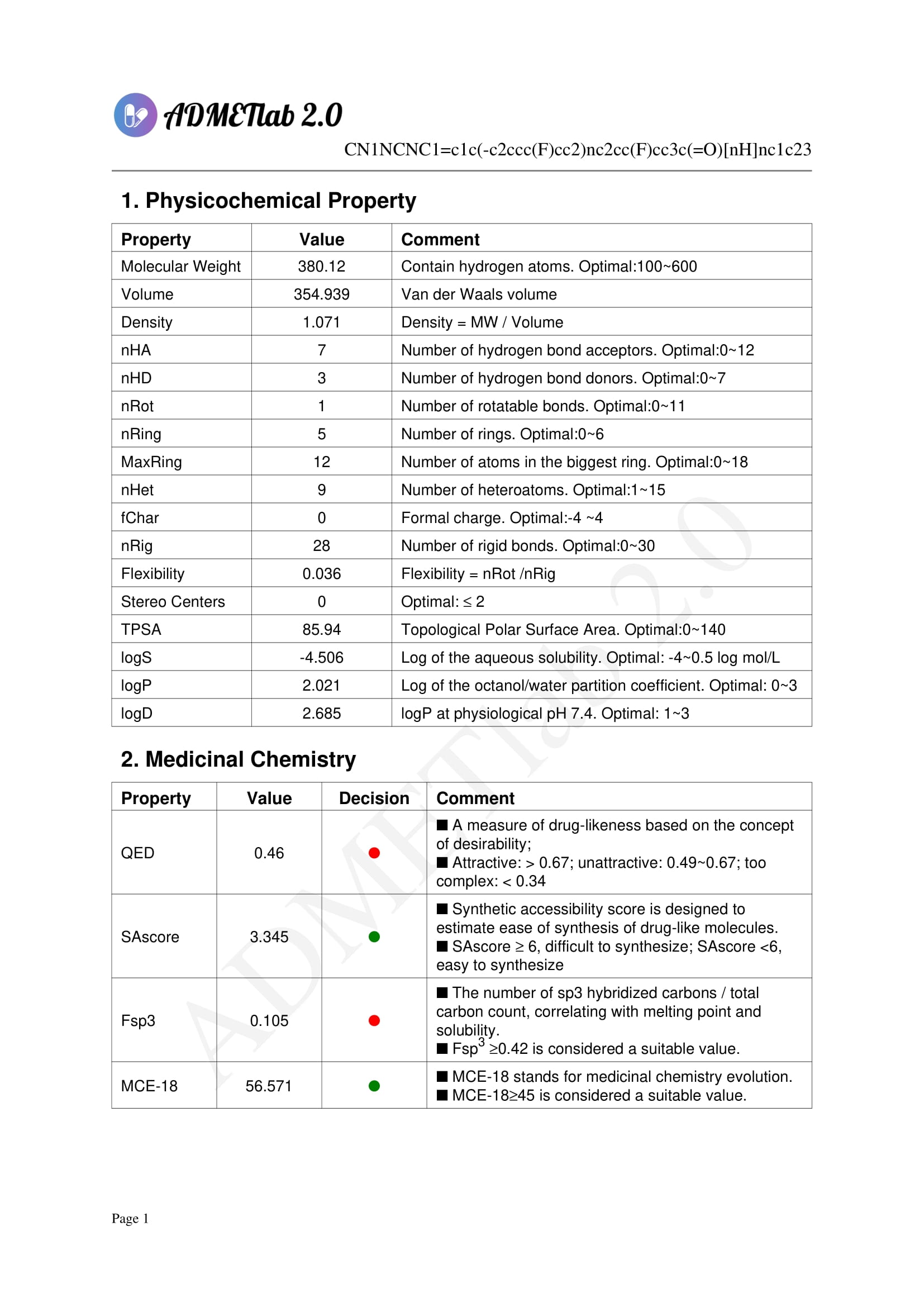

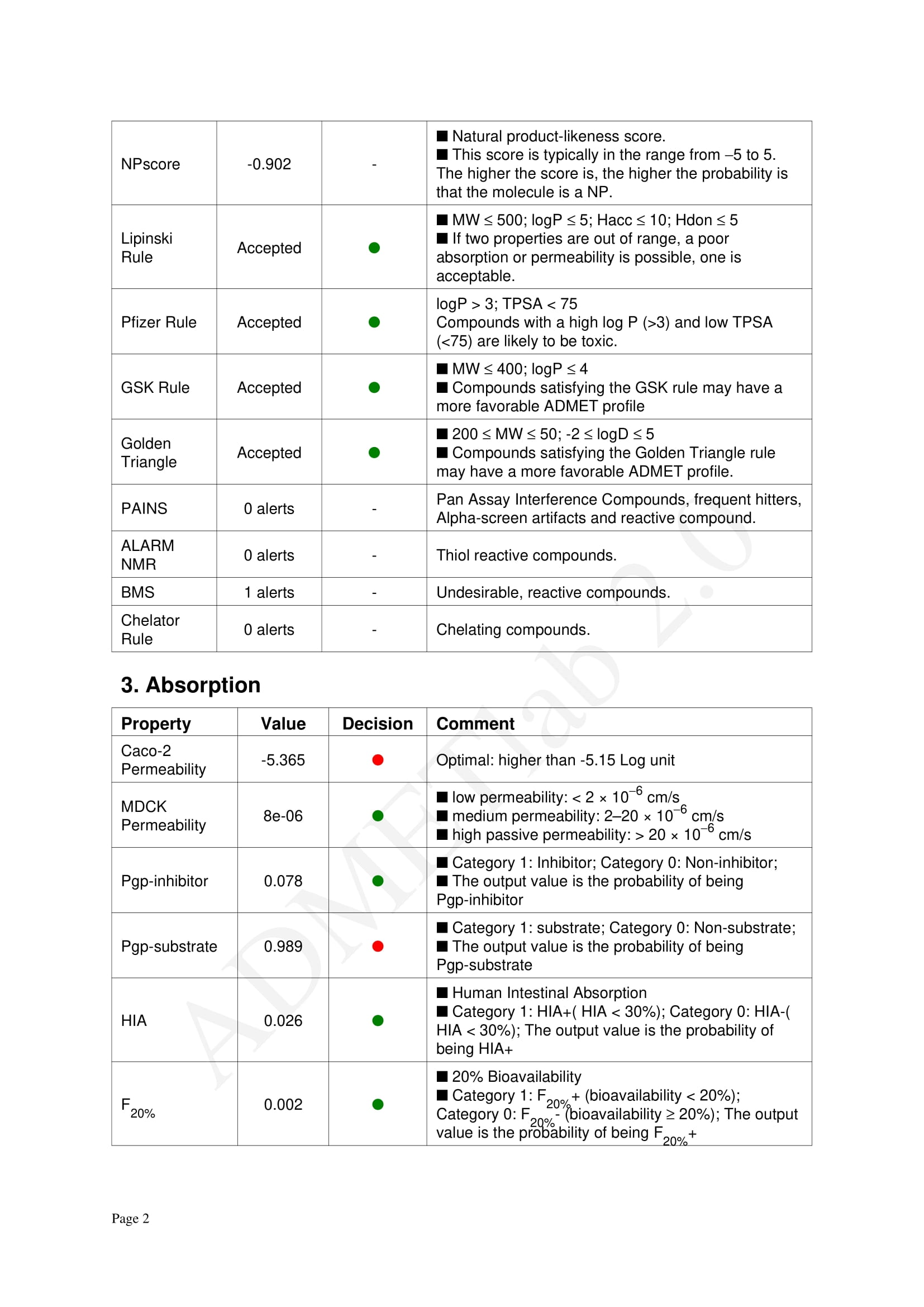

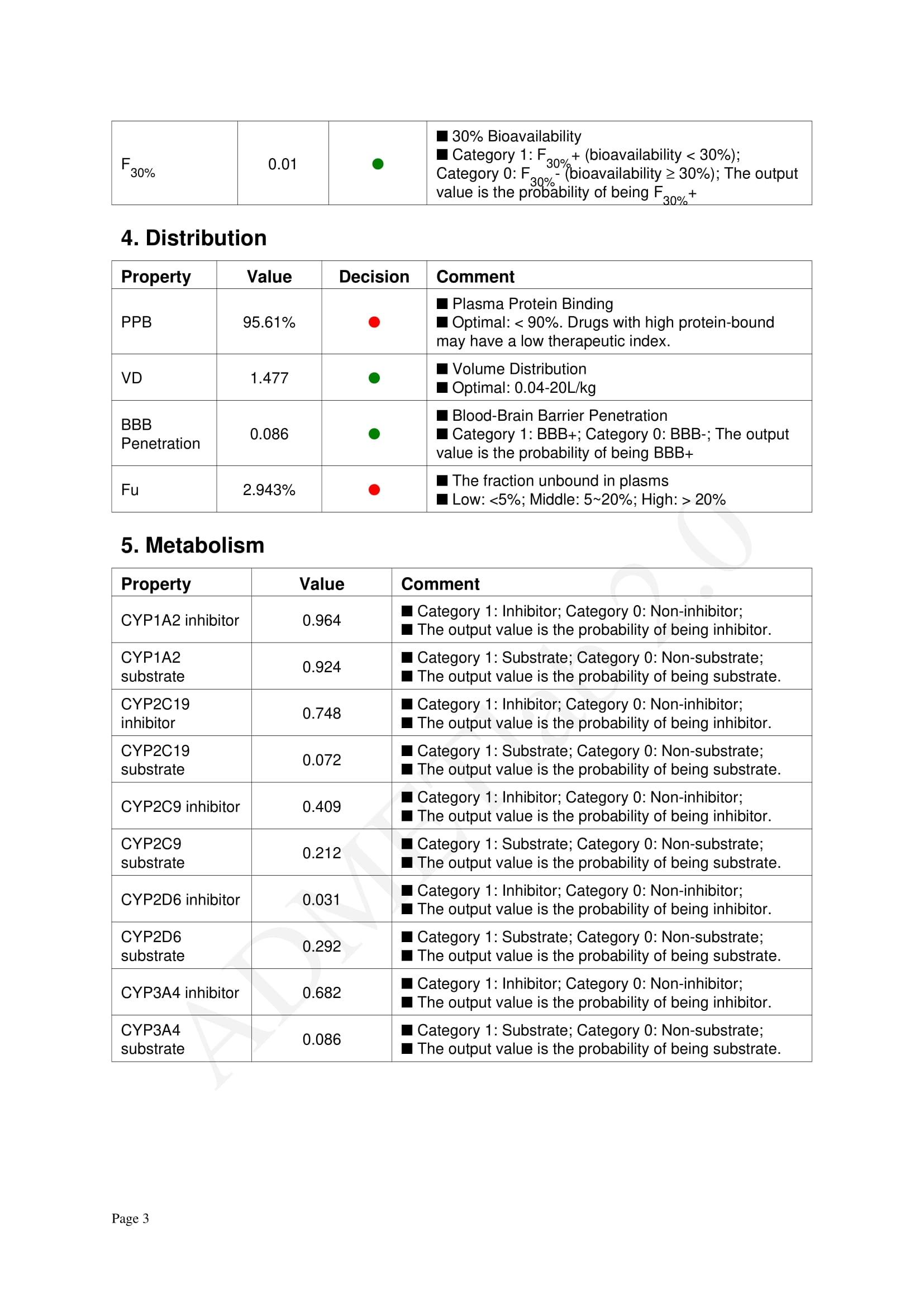

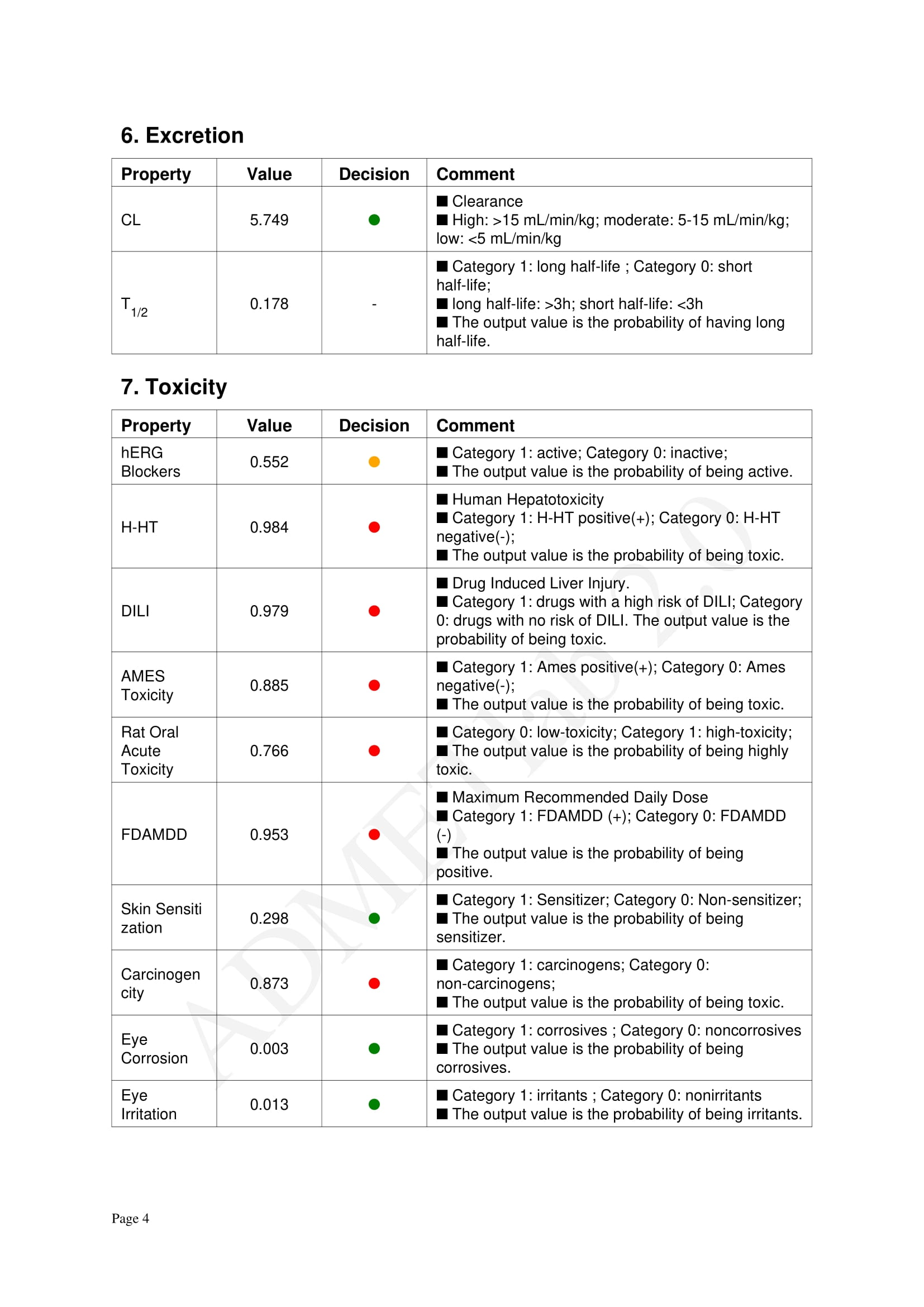

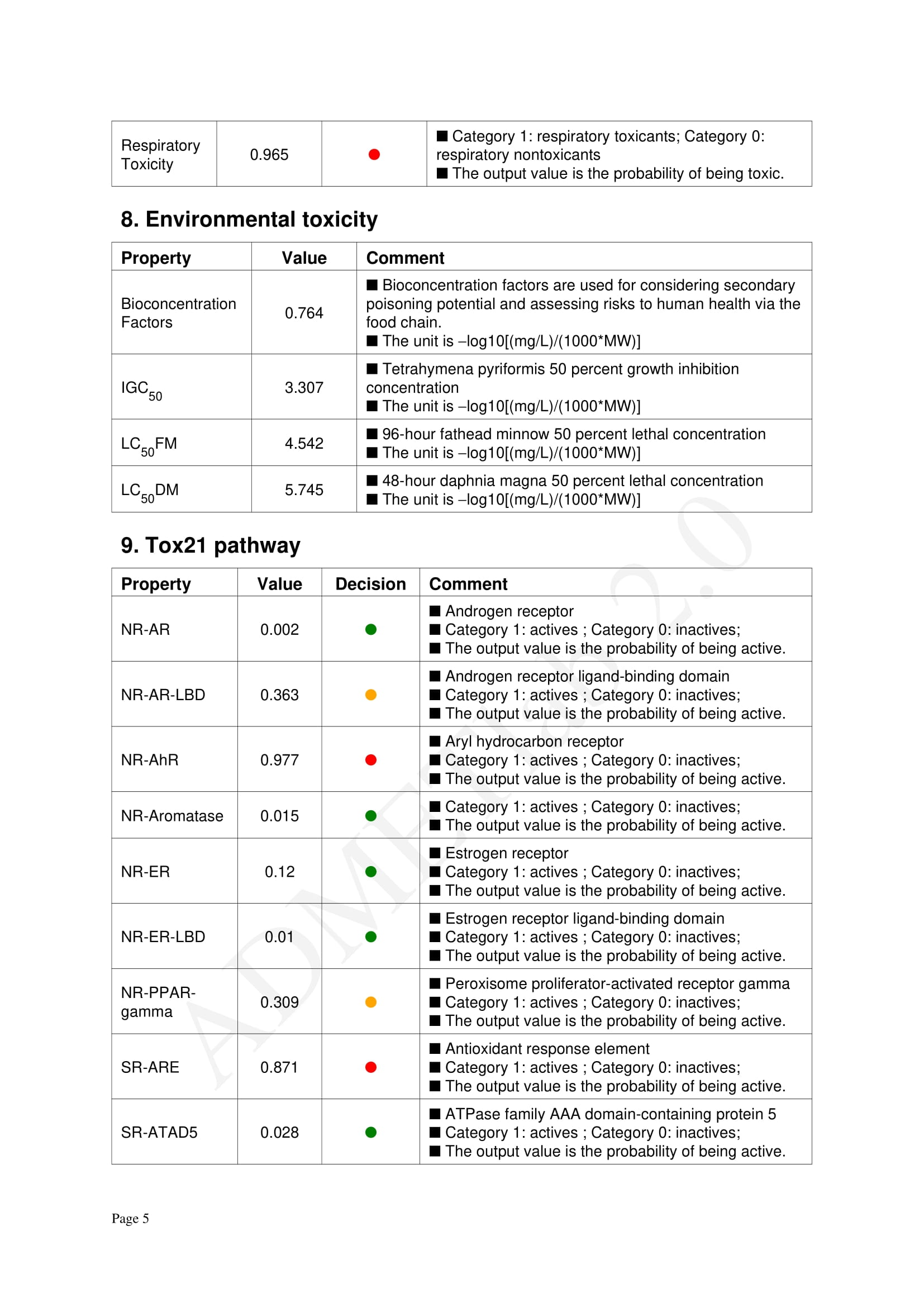

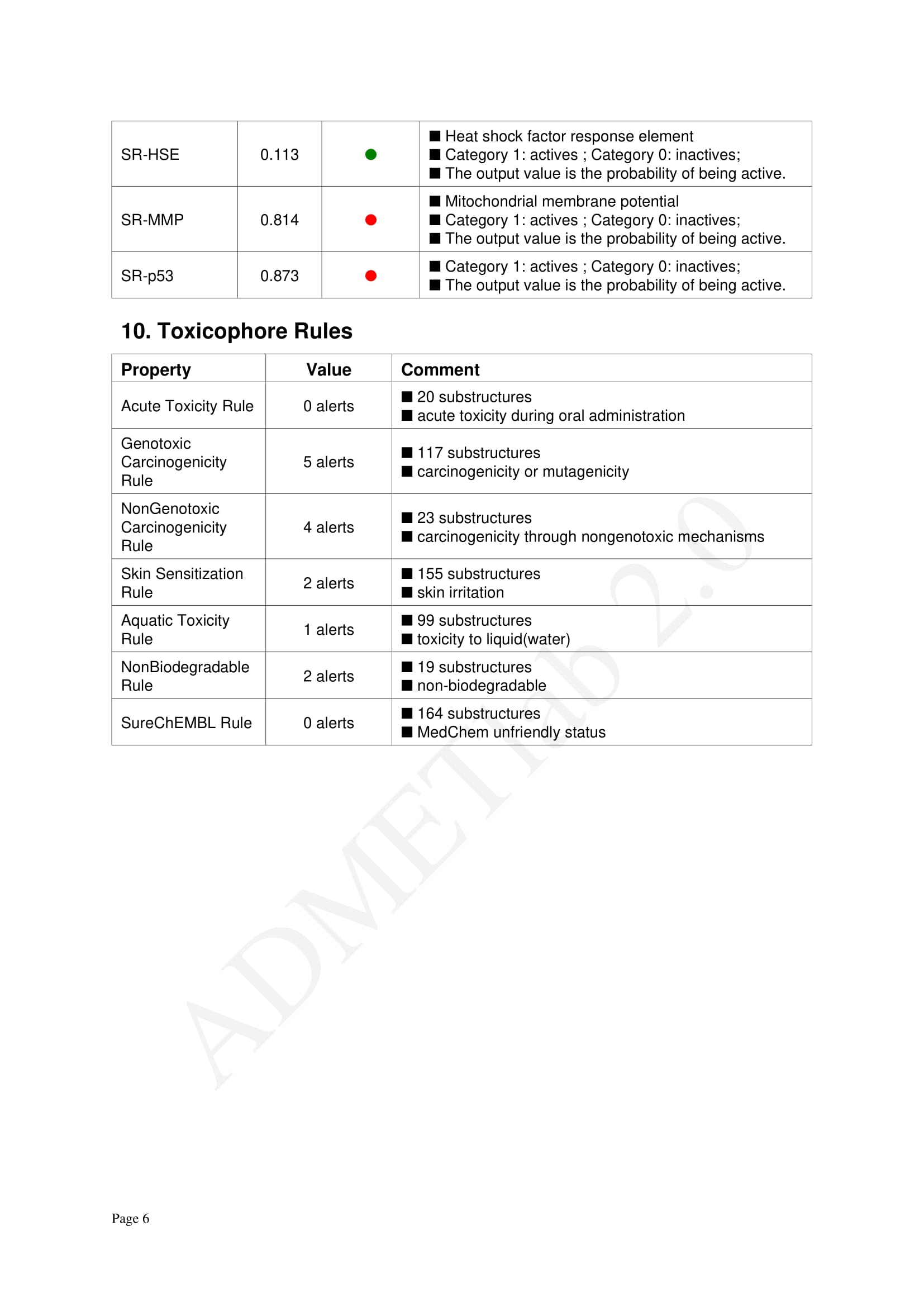

Supplement: Supplementary file 7 — Additional file 7: Data S2. Structures and ADMET (absorption, distribution, metabolism, excretion, toxicity) prediction of AZD4647, AZD7762, Danusertib, Nintedanib, OSU-03012, SNS-314, Sorafenib and Talazoparib as analyzed by the `ADMETlab 2.0’ web platform (https://admet.scbdd.com/). [file 10020_2023_636_MOESM7_ESM.docx]
